# Supplementary figures and images for: Impacts of DNA methylation on H2A.Z deposition and nucleosome stability
Source: eLife. 2026 Jul 7;15:RP109762. doi: 10.7554/eLife.109762 (PMC13341117; doi:10.7554/eLife.109762)

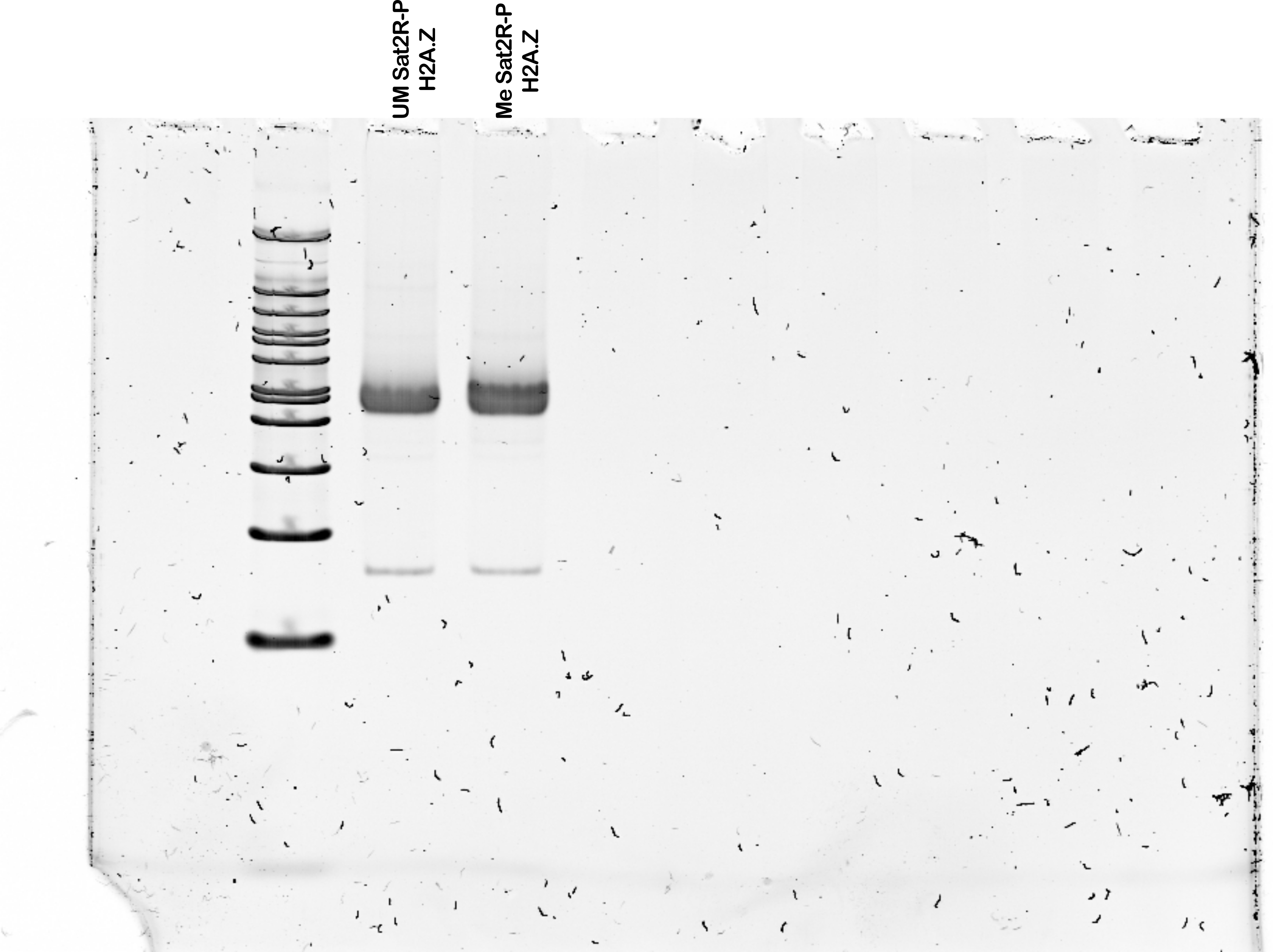

Supplement: Figure 1—figure supplement 1—source data 1. [file elife-109762-fig1-figsupp1-data1.zip › Figure 1 - figure supplement 1 - source data 1/Figure 1 - figure supplement 1 - source data 1 - 1A.png]

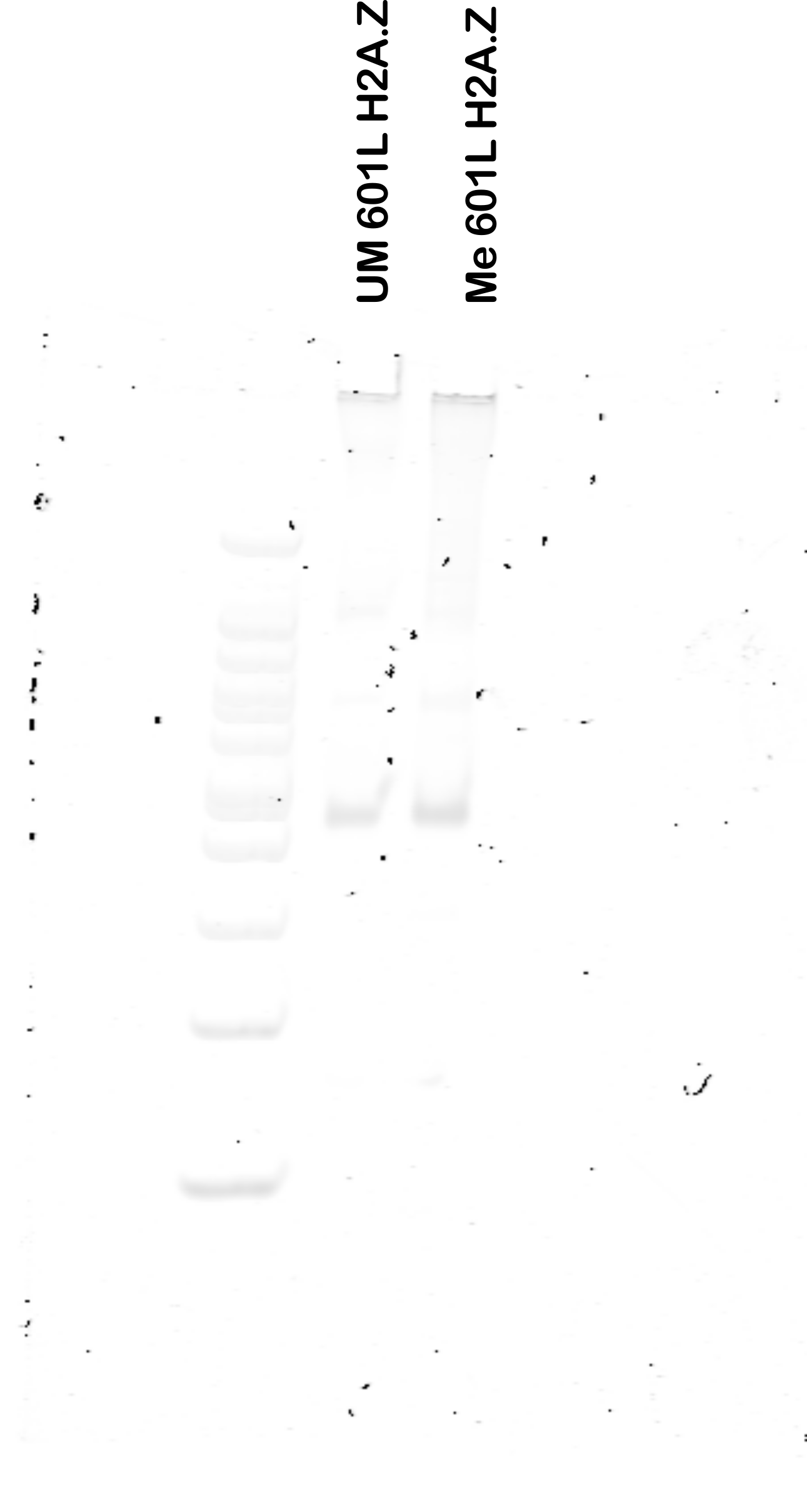

Supplement: Figure 2—figure supplement 4—source data 1. [file elife-109762-fig2-figsupp4-data1.zip › Figure 2 - figure supplement 4 - source data 1/Figure 2 - figure supplement 4 - source data 1 - 4A.png]

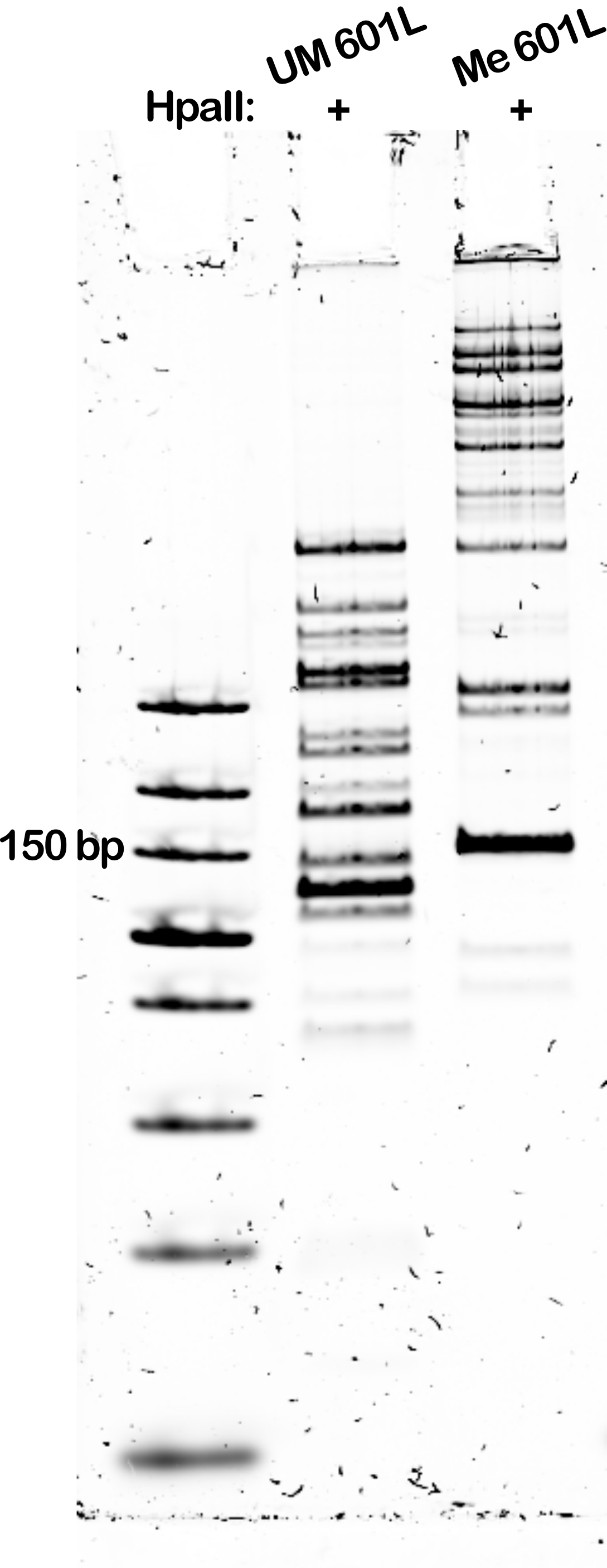

Supplement: Figure 2—figure supplement 4—source data 1. [file elife-109762-fig2-figsupp4-data1.zip › Figure 2 - figure supplement 4 - source data 1/Figure 2 - figure supplement 4 - source data 1 - 4B.png]

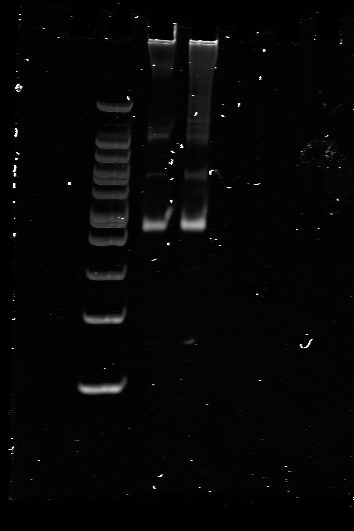

Supplement: Figure 2—figure supplement 4—source data 2. [file elife-109762-fig2-figsupp4-data2.zip › Figure 2 - figure supplement 4 - source data 2/Figure 2 - figure supplement 4 - source data 2 - 4A.TIF]

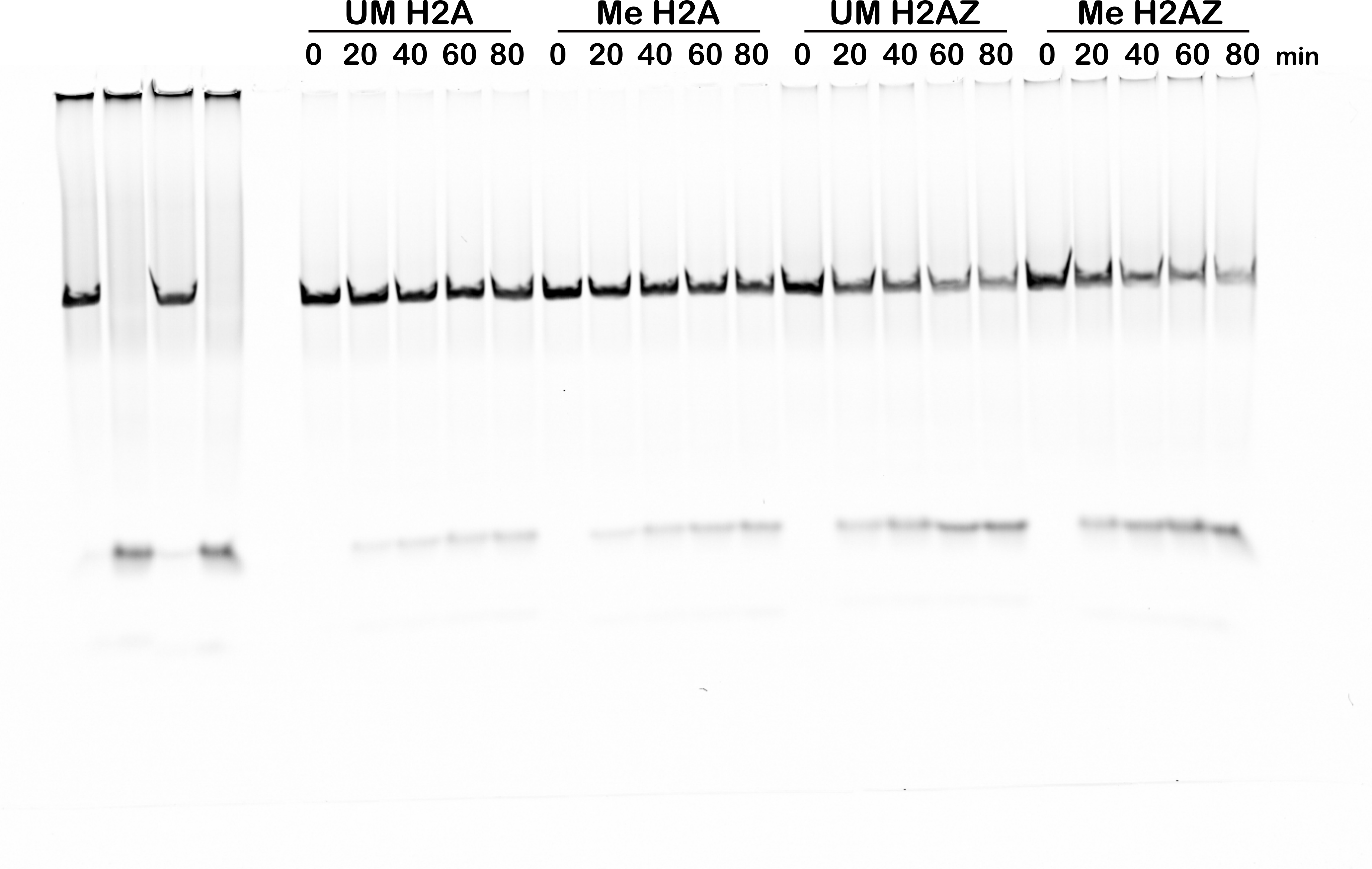

Supplement: Figure 3—source data 1. [file elife-109762-fig3-data1.zip › Figure 3 - source data 1/Figure 3 - source data 1 - cy5.png]

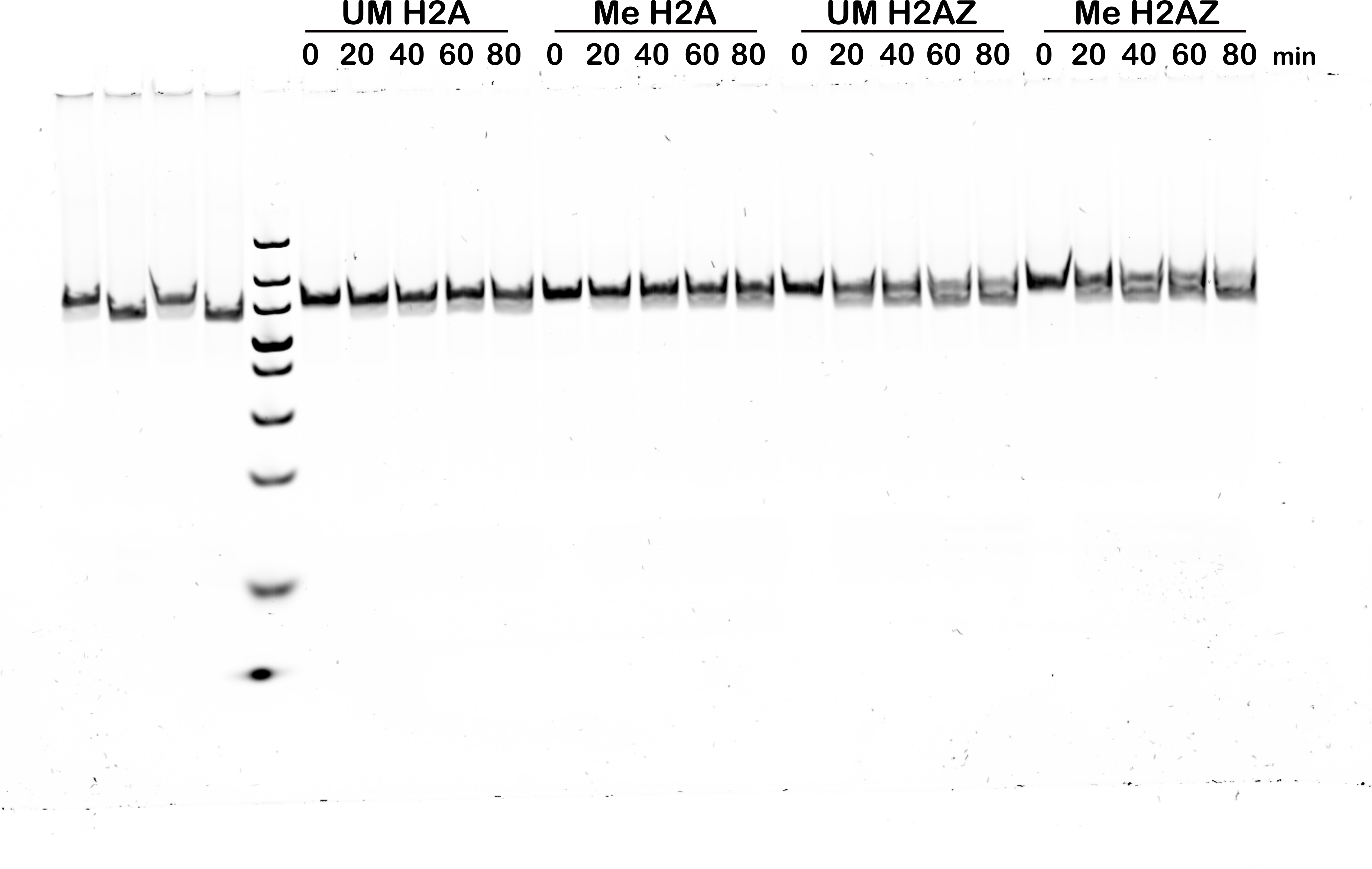

Supplement: Figure 3—source data 1. [file elife-109762-fig3-data1.zip › Figure 3 - source data 1/Figure 3 - source data 1 - sybrsafe.png]

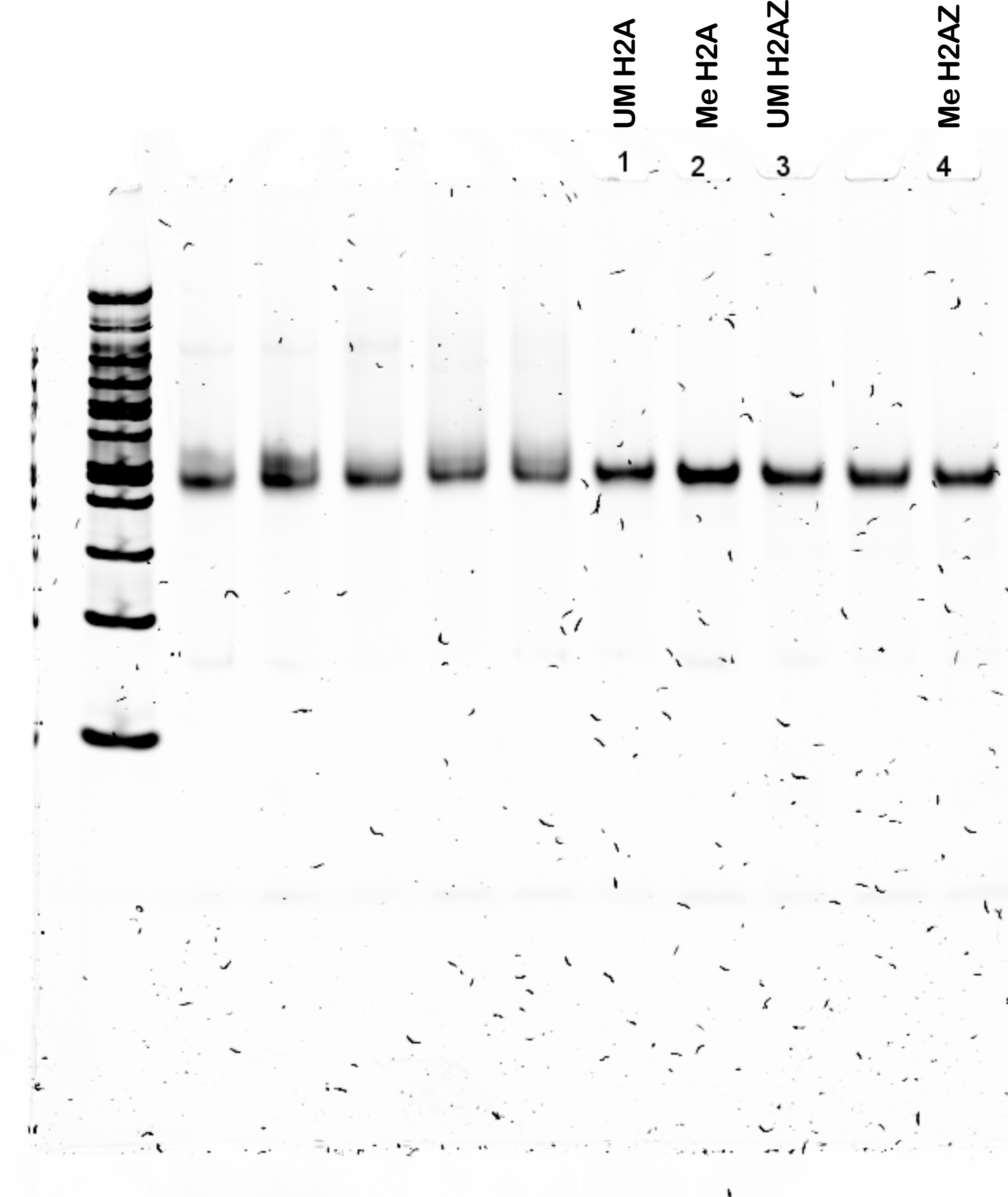

Supplement: Figure 3—figure supplement 1—source data 1. [file elife-109762-fig3-figsupp1-data1.zip › Figure 3 - figure supplement 1 - source data 1/Figure 3 - figure supplement 1 - source data 1 - 1A.png]

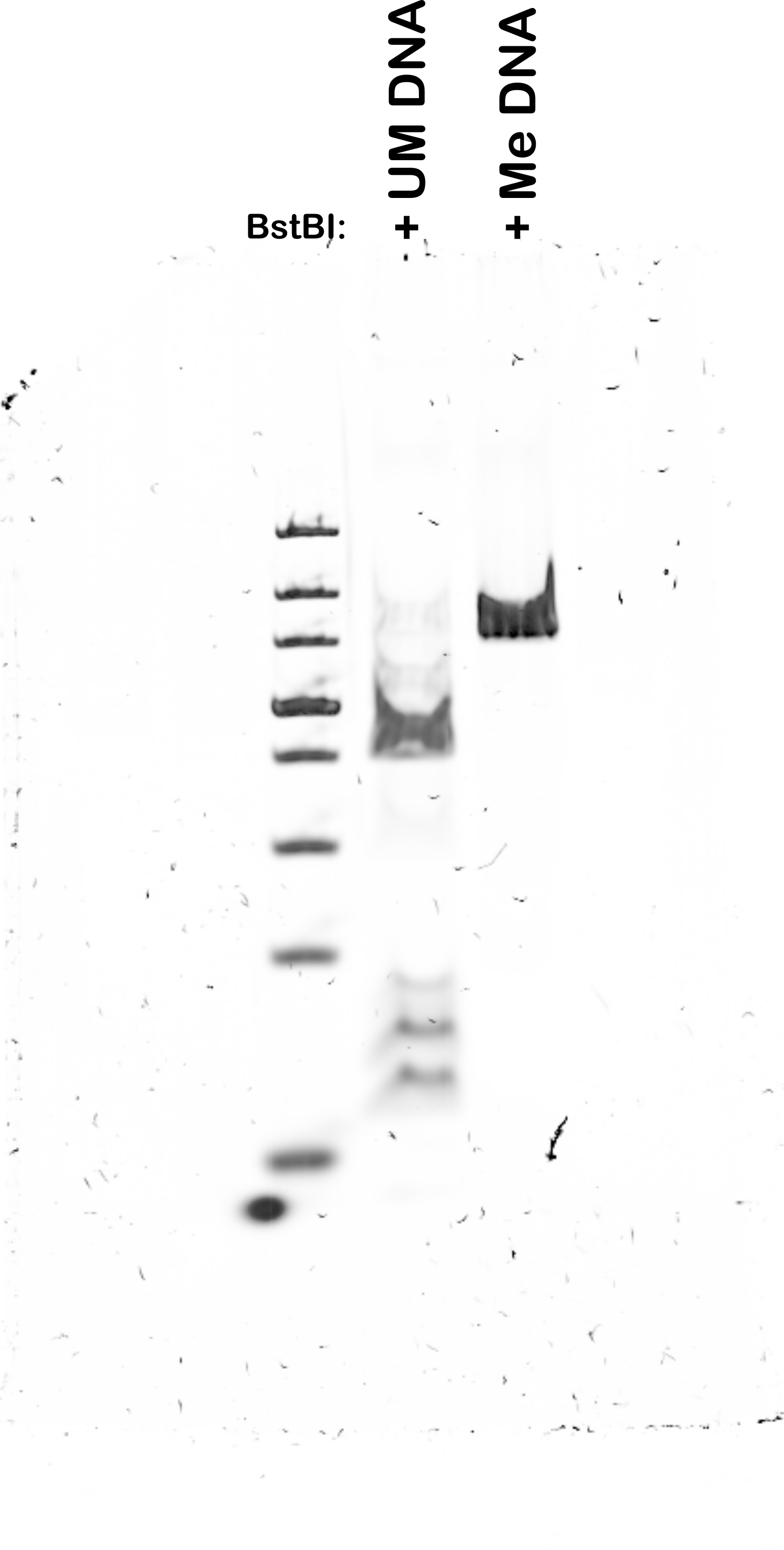

Supplement: Figure 3—figure supplement 1—source data 1. [file elife-109762-fig3-figsupp1-data1.zip › Figure 3 - figure supplement 1 - source data 1/Figure 3 - figure supplement 1 - source data 1 - 1D.png]

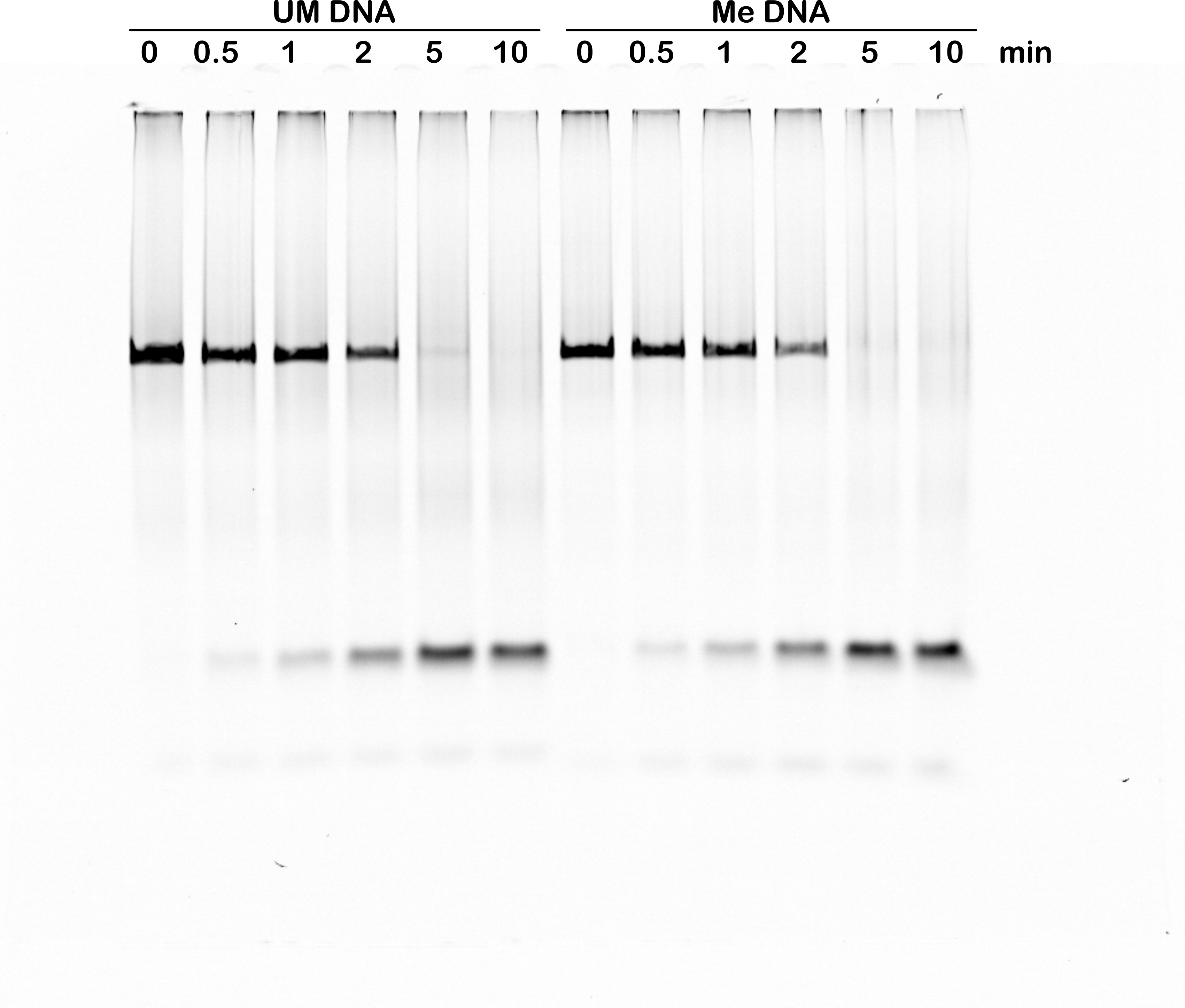

Supplement: Figure 3—figure supplement 1—source data 1. [file elife-109762-fig3-figsupp1-data1.zip › Figure 3 - figure supplement 1 - source data 1/Figure 3 - figure supplement 1 - source data 1 - 1E- cy5.png]

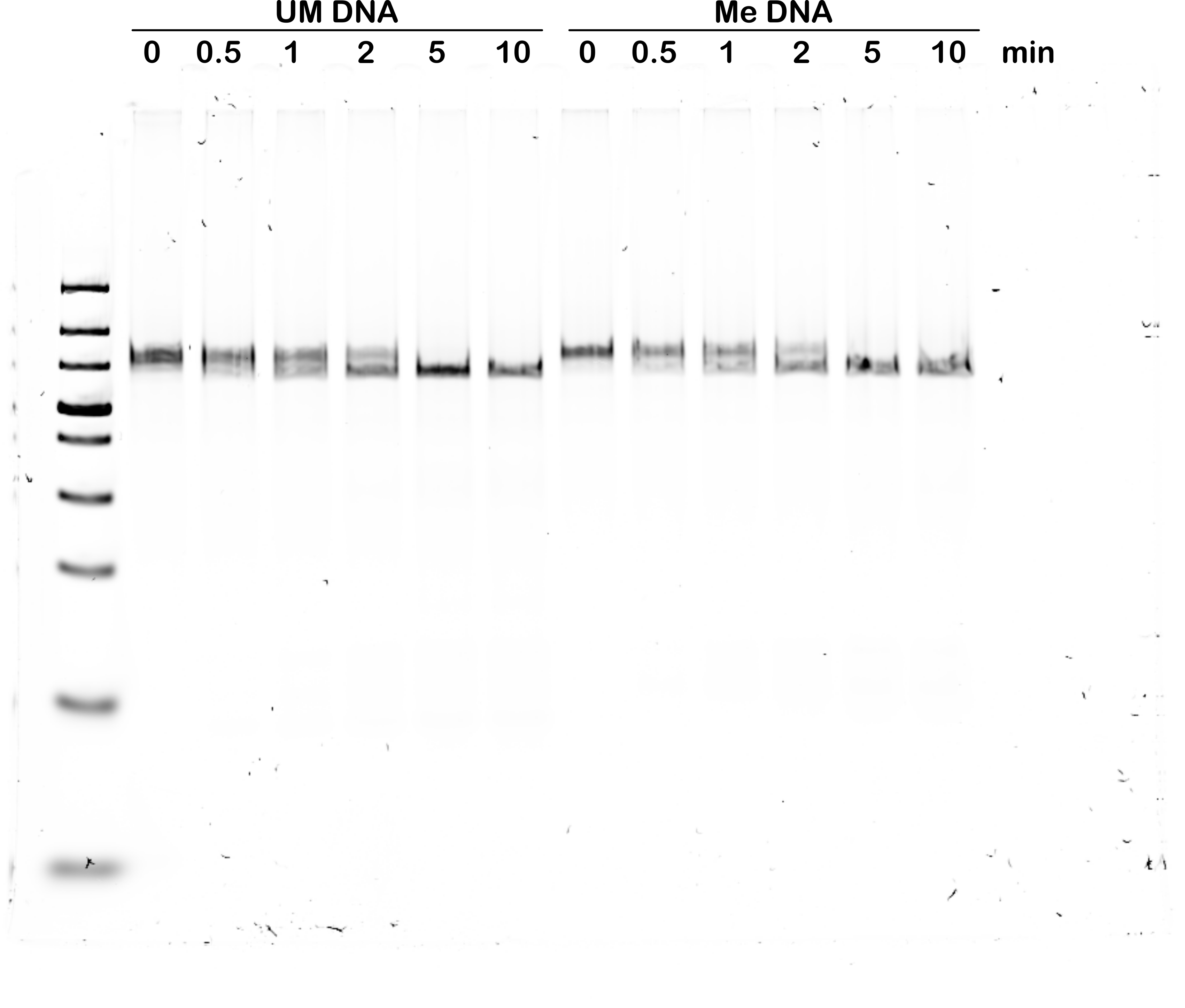

Supplement: Figure 3—figure supplement 1—source data 1. [file elife-109762-fig3-figsupp1-data1.zip › Figure 3 - figure supplement 1 - source data 1/Figure 3 - figure supplement 1 - source data 1 - 1E - sybrsafe.png]

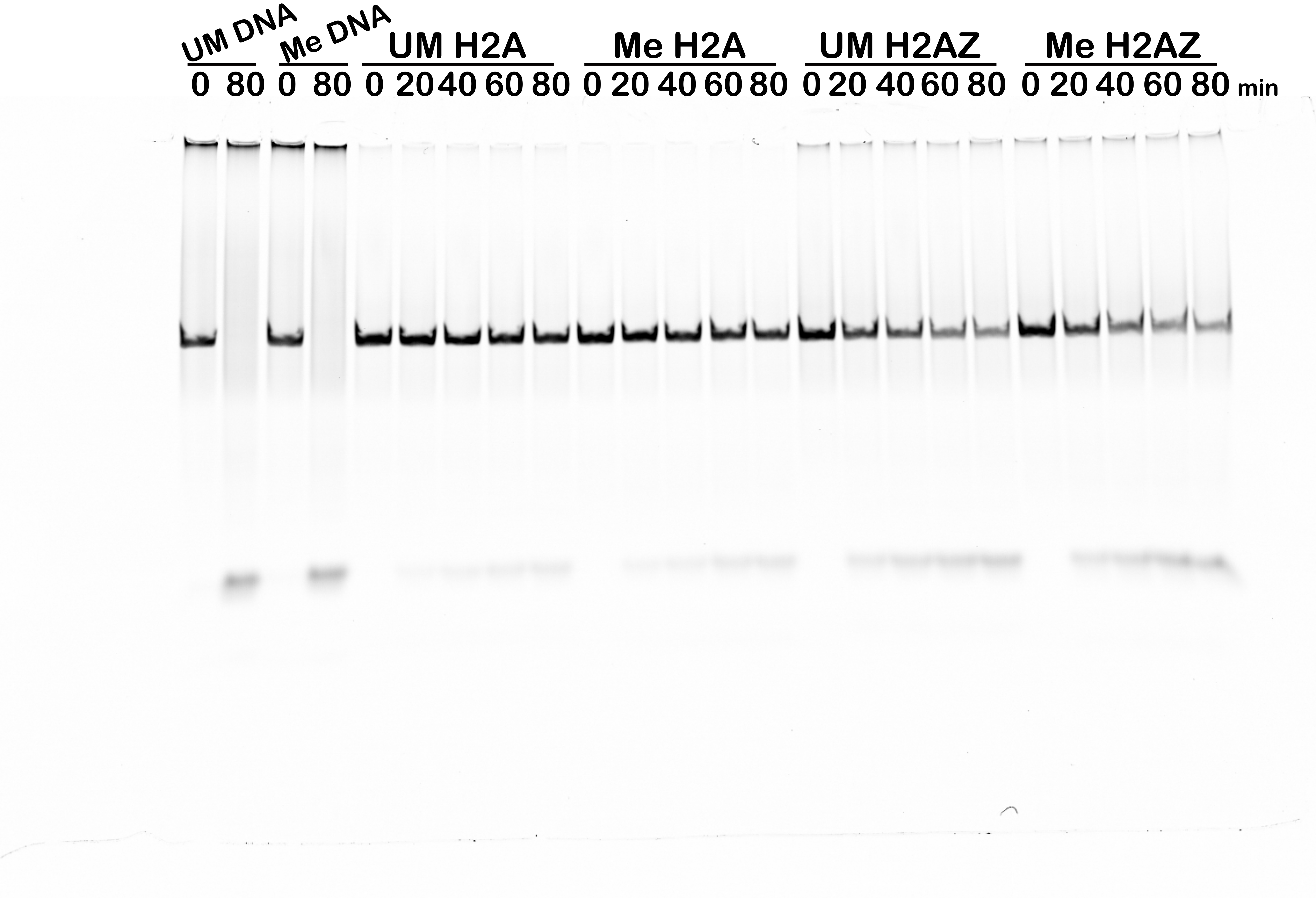

Supplement: Figure 3—figure supplement 2—source data 1. [file elife-109762-fig3-figsupp2-data1.zip › Figure 3 - figure supplement 2 - source data 1/Figure 3 - figure supplement 2 - source data 1 - Rep2 - cy5 - LABELED.png]

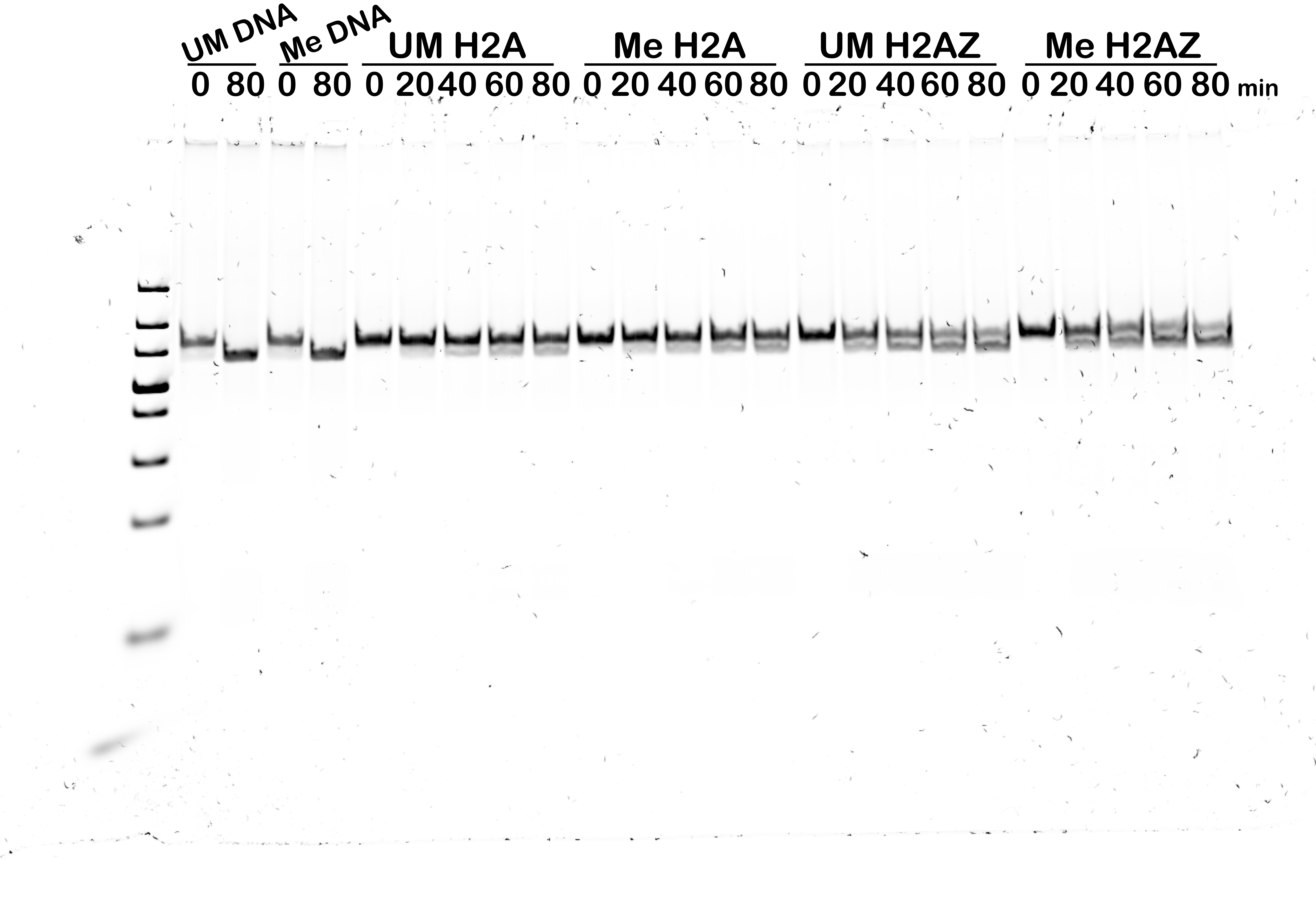

Supplement: Figure 3—figure supplement 2—source data 1. [file elife-109762-fig3-figsupp2-data1.zip › Figure 3 - figure supplement 2 - source data 1/Figure 3 - figure supplement 2 - source data 1 - Rep2 - sybrsafe - LABELED.png]

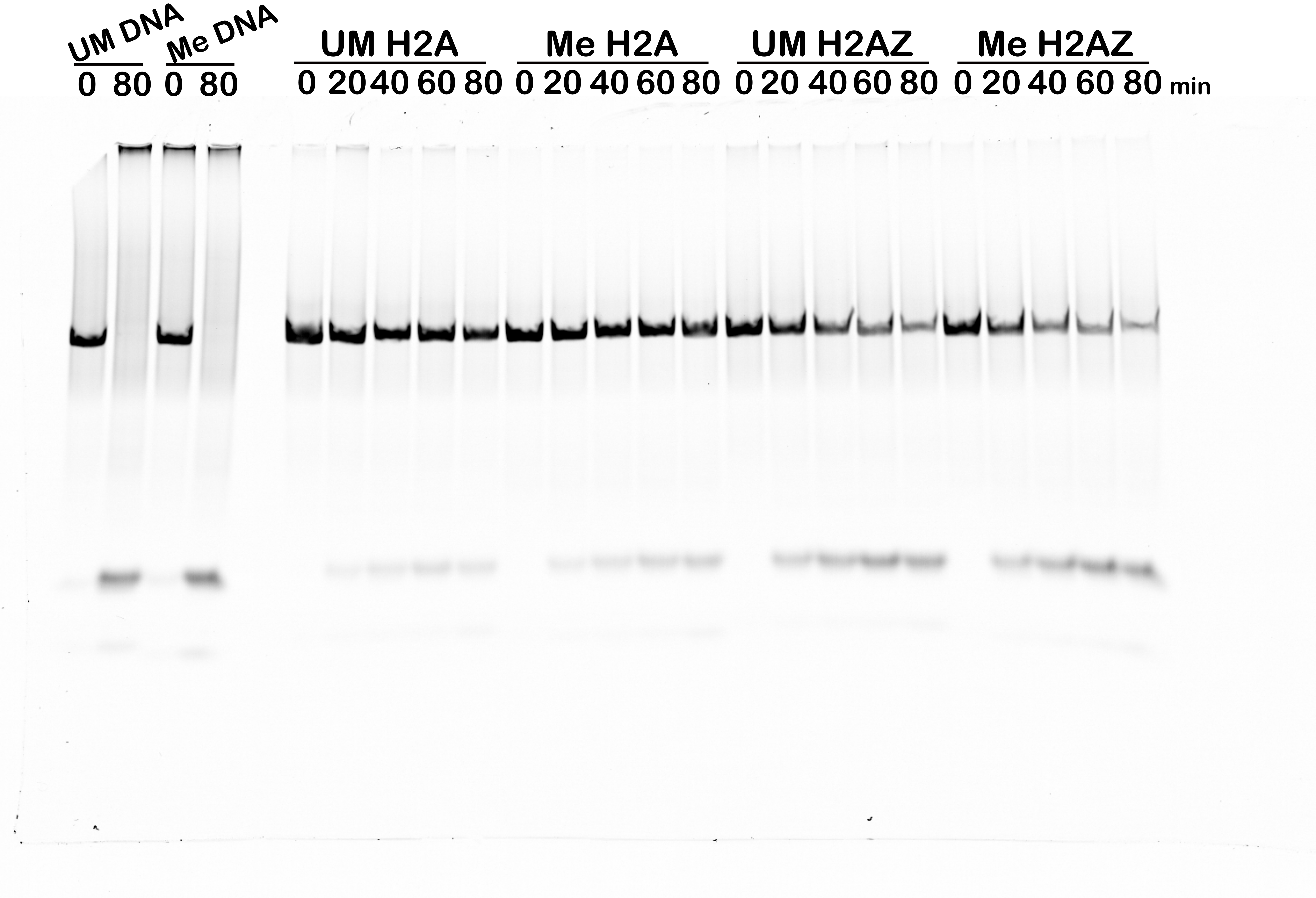

Supplement: Figure 3—figure supplement 2—source data 1. [file elife-109762-fig3-figsupp2-data1.zip › Figure 3 - figure supplement 2 - source data 1/Figure 3 - figure supplement 2 - source data 1 - Rep3 - cy5 - LABELED.png]

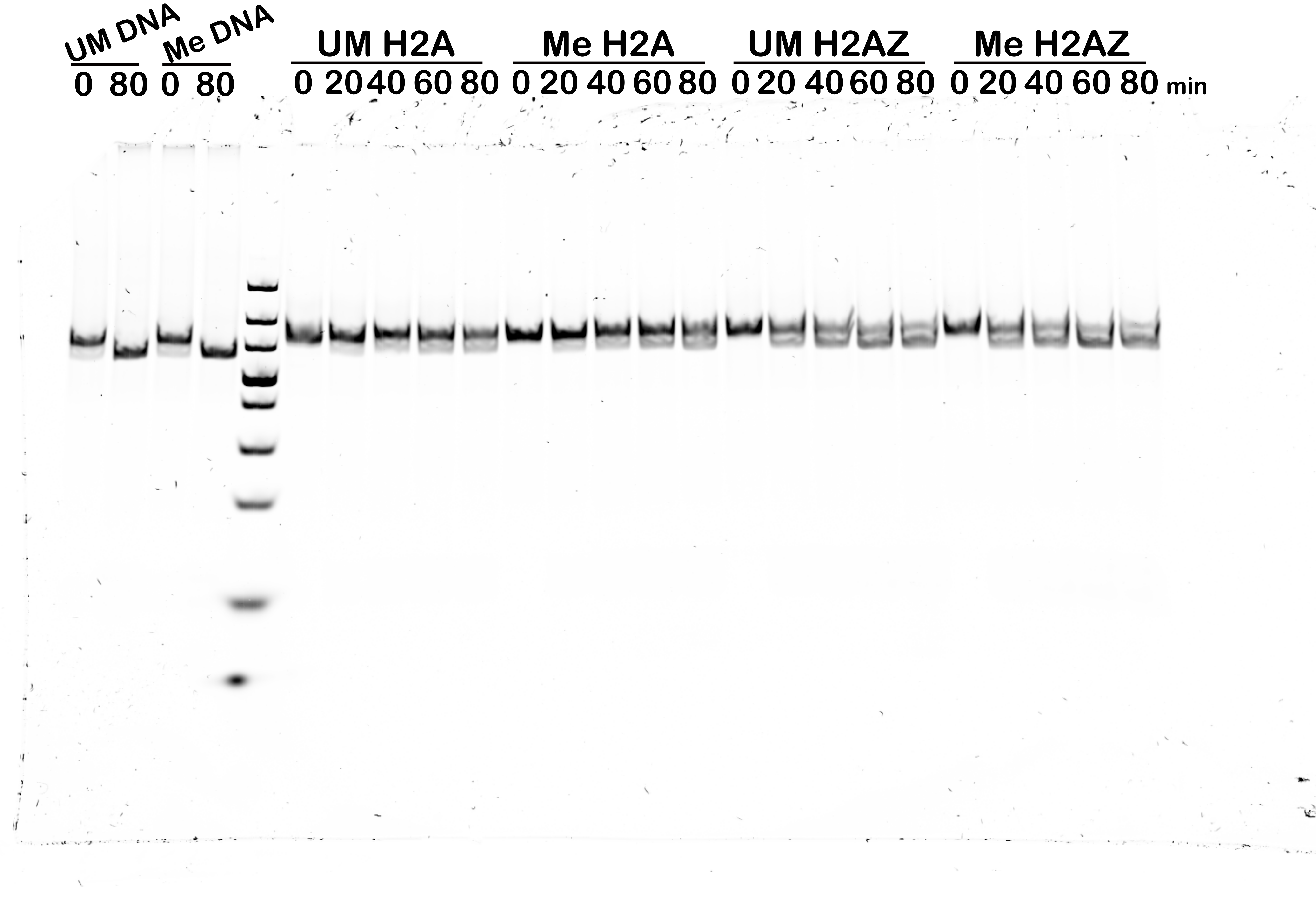

Supplement: Figure 3—figure supplement 2—source data 1. [file elife-109762-fig3-figsupp2-data1.zip › Figure 3 - figure supplement 2 - source data 1/Figure 3 - figure supplement 2 - source data 1 - Rep3 - sybrsafe - LABELED.png]

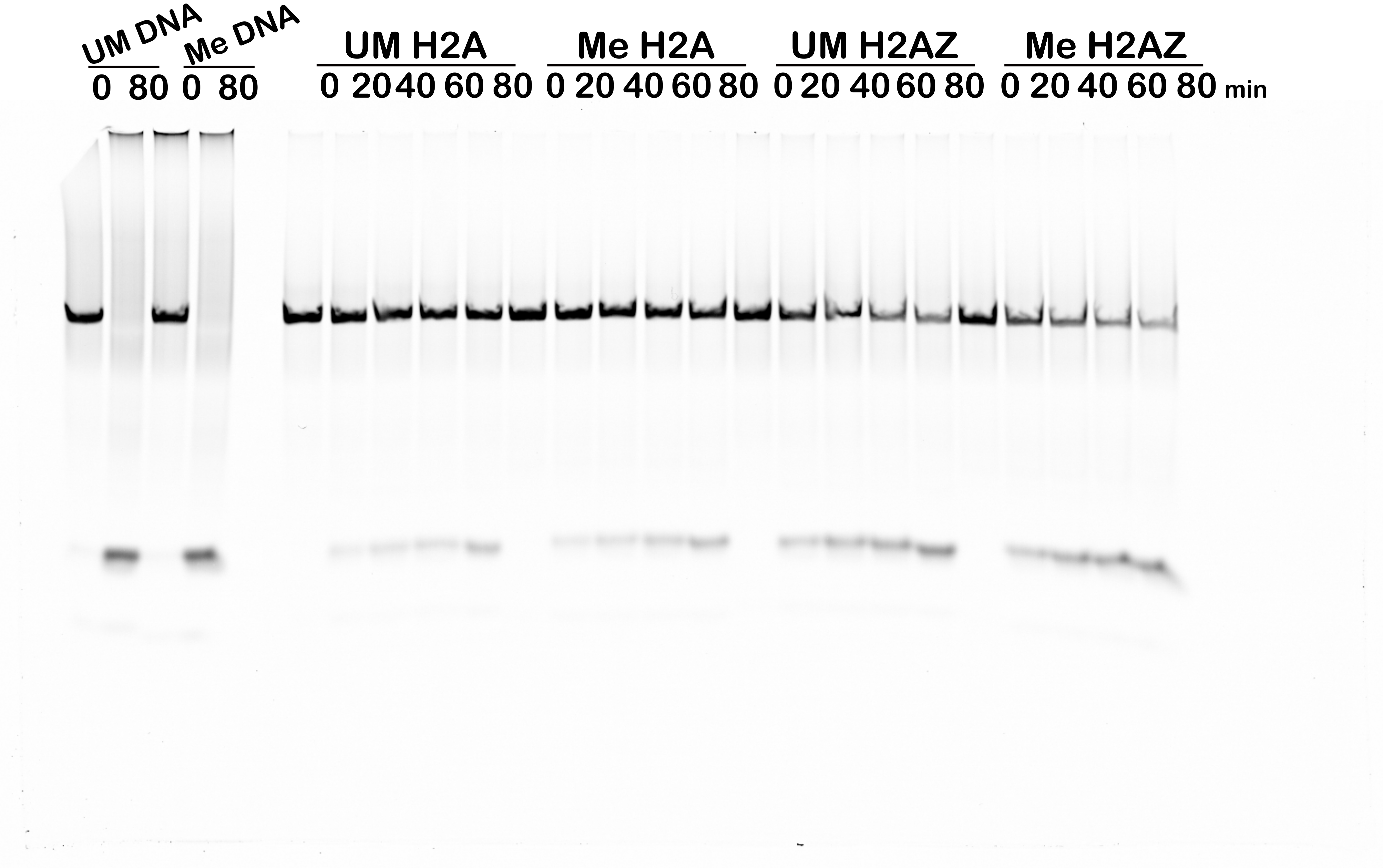

Supplement: Figure 3—figure supplement 2—source data 1. [file elife-109762-fig3-figsupp2-data1.zip › Figure 3 - figure supplement 2 - source data 1/Figure 3 - figure supplement 2 - source data 1 - Rep4 - cy5 - LABELED.png]

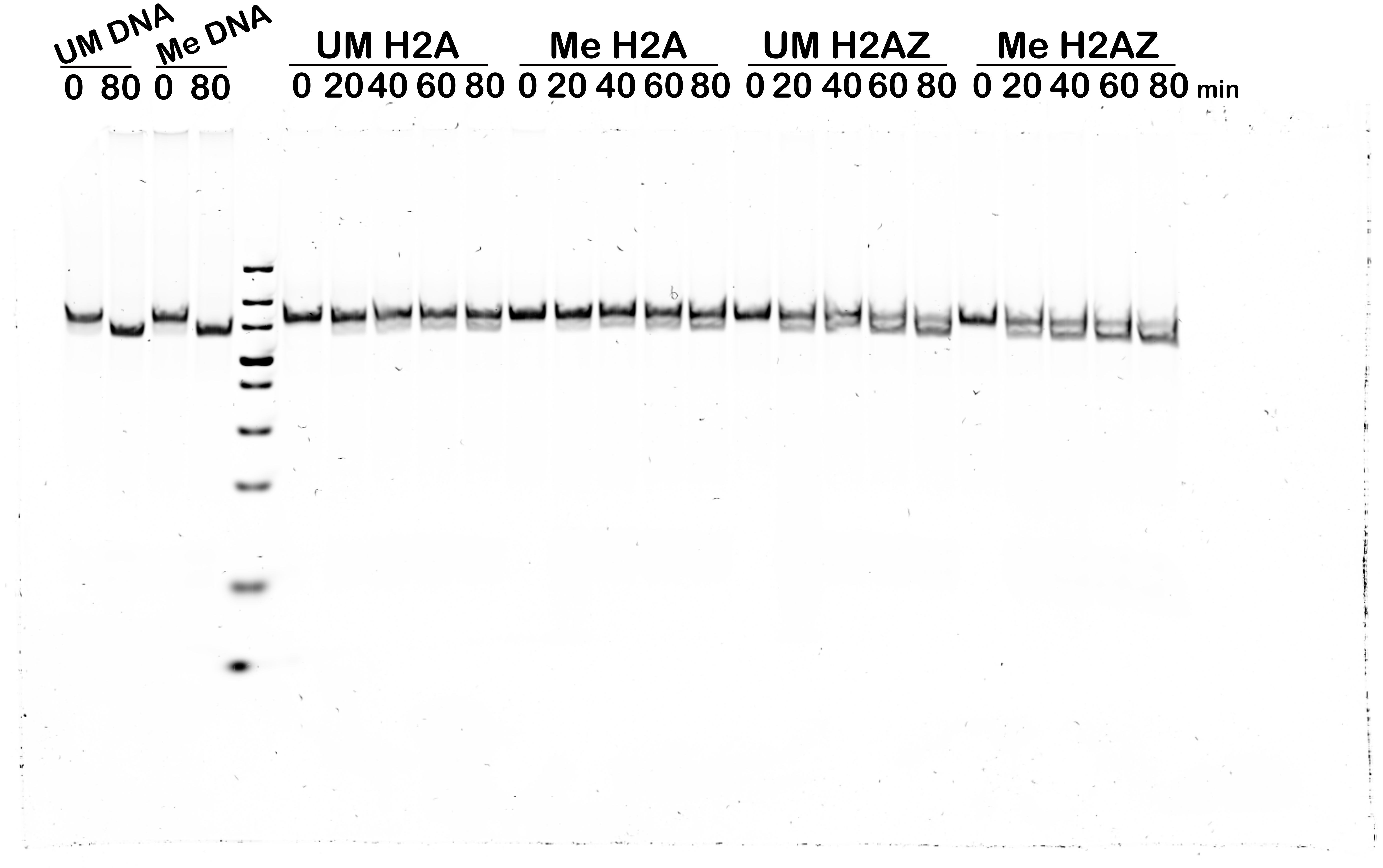

Supplement: Figure 3—figure supplement 2—source data 1. [file elife-109762-fig3-figsupp2-data1.zip › Figure 3 - figure supplement 2 - source data 1/Figure 3 - figure supplement 2 - source data 1 - Rep4 - sybrsafe - LABELED.png]

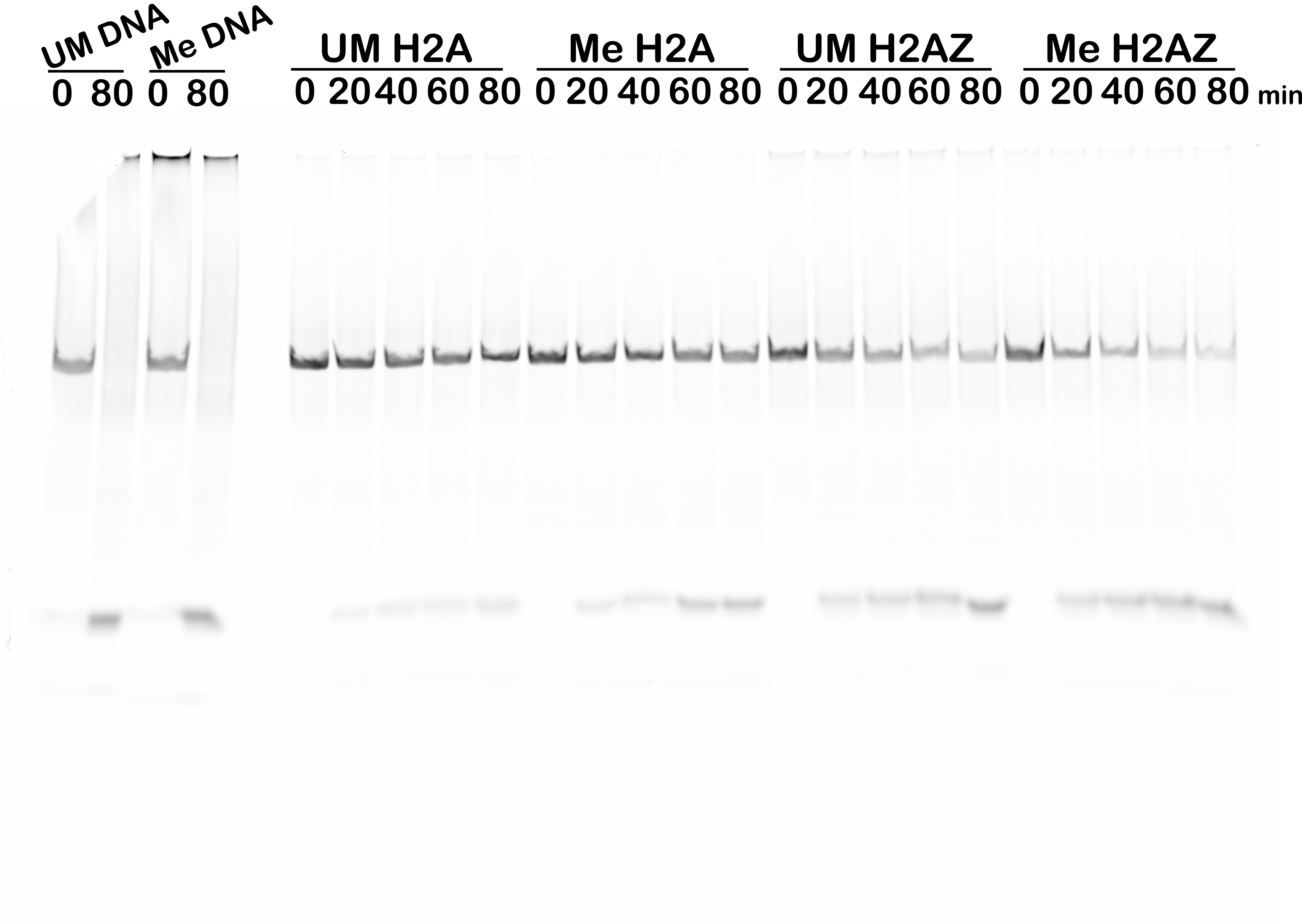

Supplement: Figure 3—figure supplement 2—source data 1. [file elife-109762-fig3-figsupp2-data1.zip › Figure 3 - figure supplement 2 - source data 1/Figure 3 - figure supplement 2 - source data 1 - Rep5 - cy5 - LABELED.png]

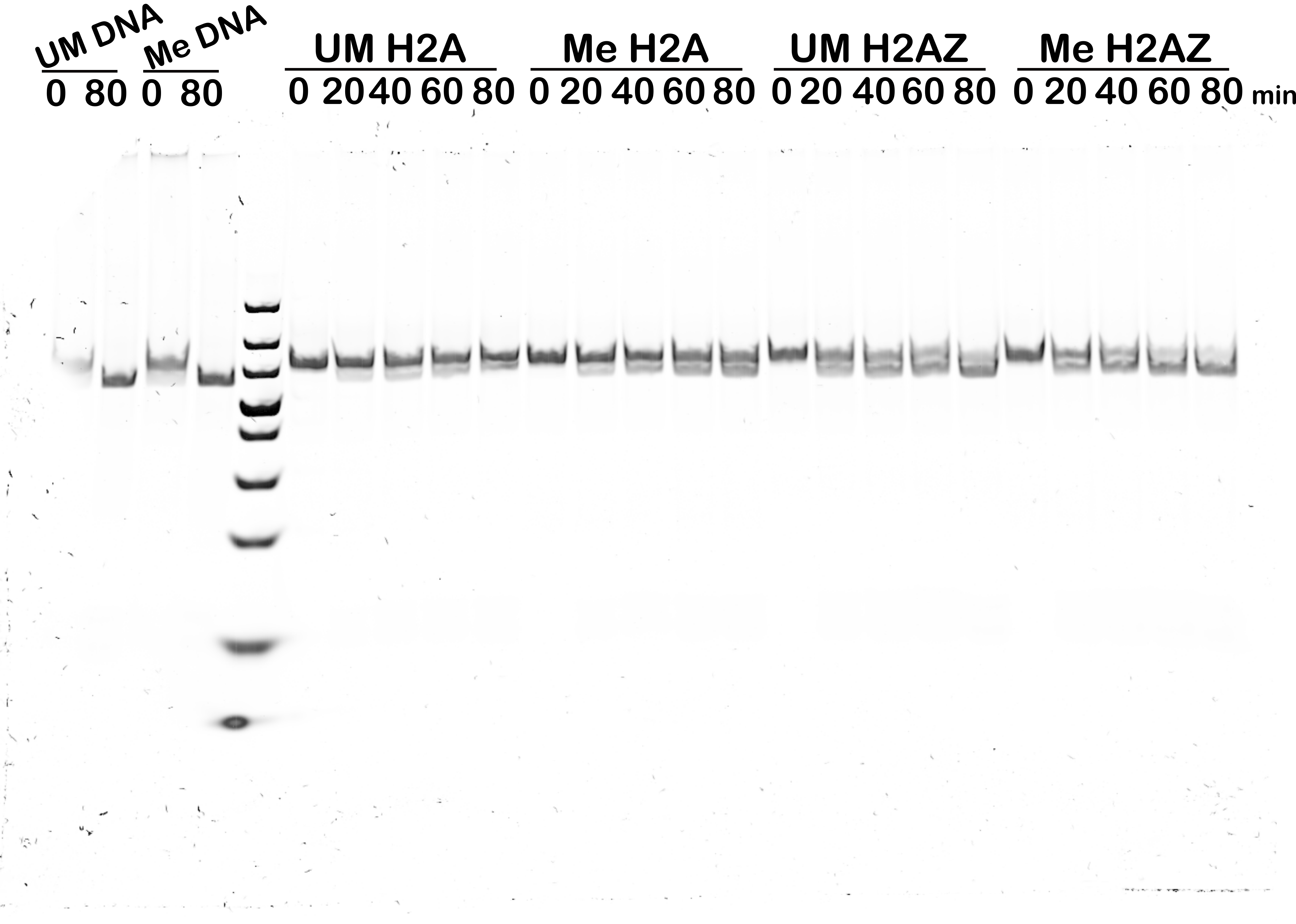

Supplement: Figure 3—figure supplement 2—source data 1. [file elife-109762-fig3-figsupp2-data1.zip › Figure 3 - figure supplement 2 - source data 1/Figure 3 - figure supplement 2 - source data 1 - Rep5 - sybrsafe - LABELED.png]

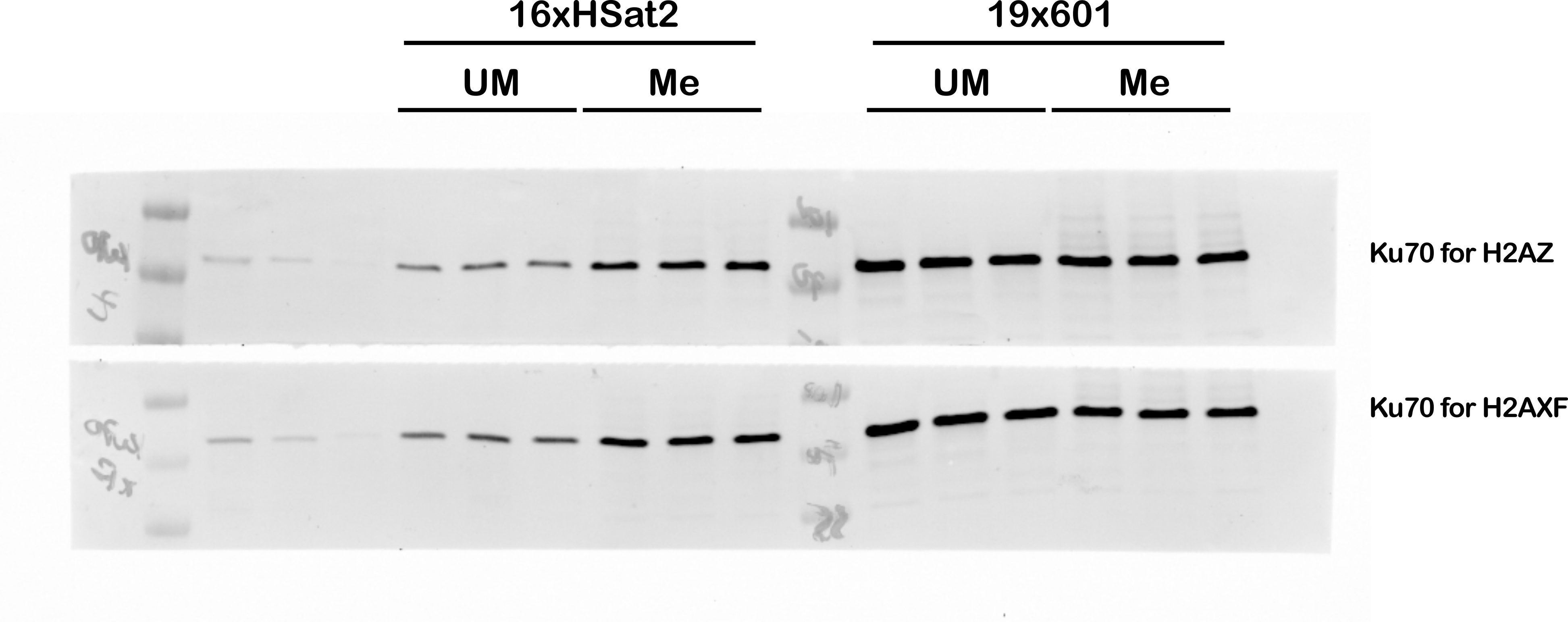

Supplement: Figure 4—source data 1. [file elife-109762-fig4-data1.zip › Figure 4 - source data 1/Figure 4 - source data 1 - 4C - Ku70 - LABELED.png]

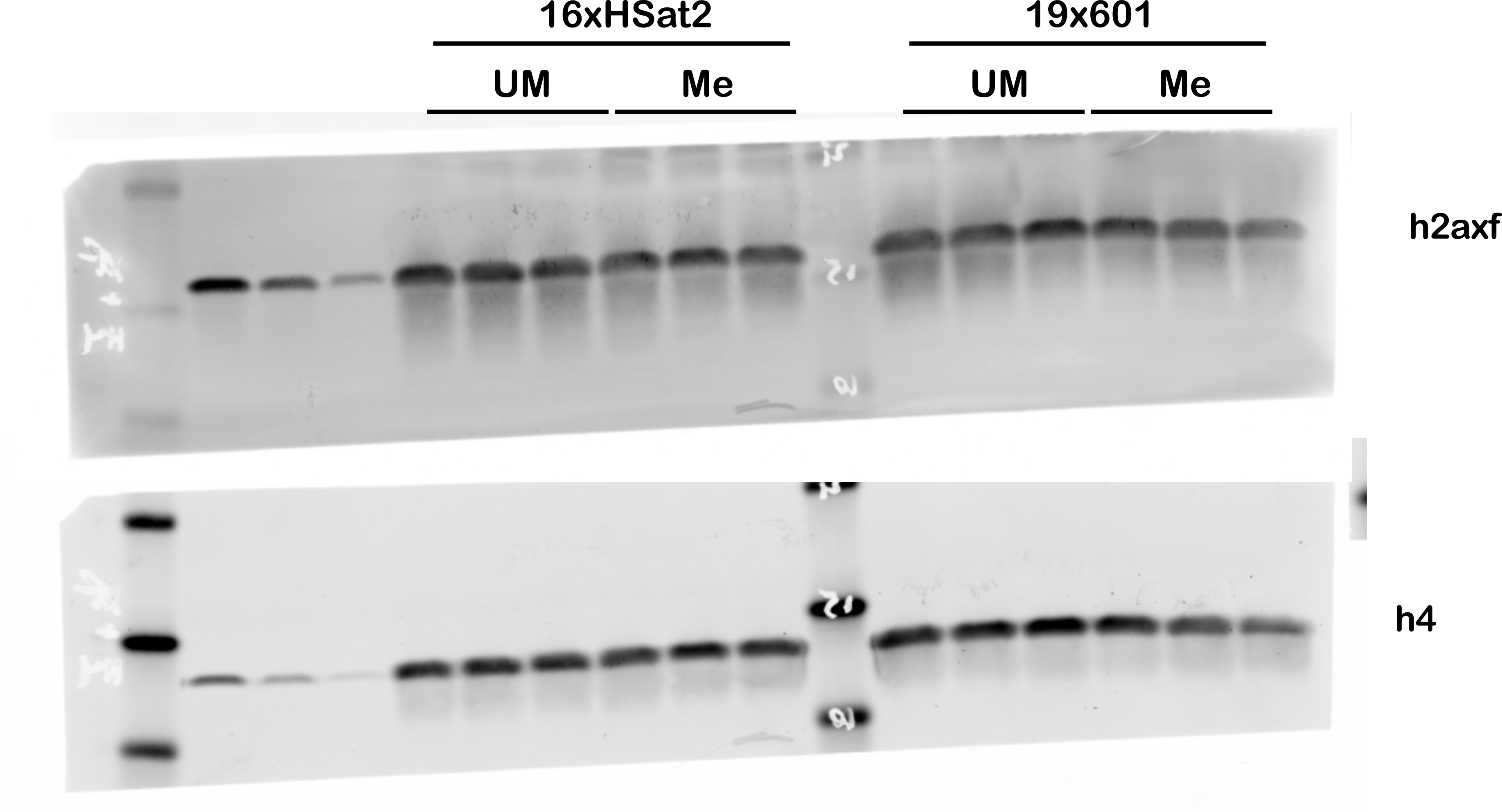

Supplement: Figure 4—source data 1. [file elife-109762-fig4-data1.zip › Figure 4 - source data 1/Figure 4 - source data 1 - 4E - LABELED.png]

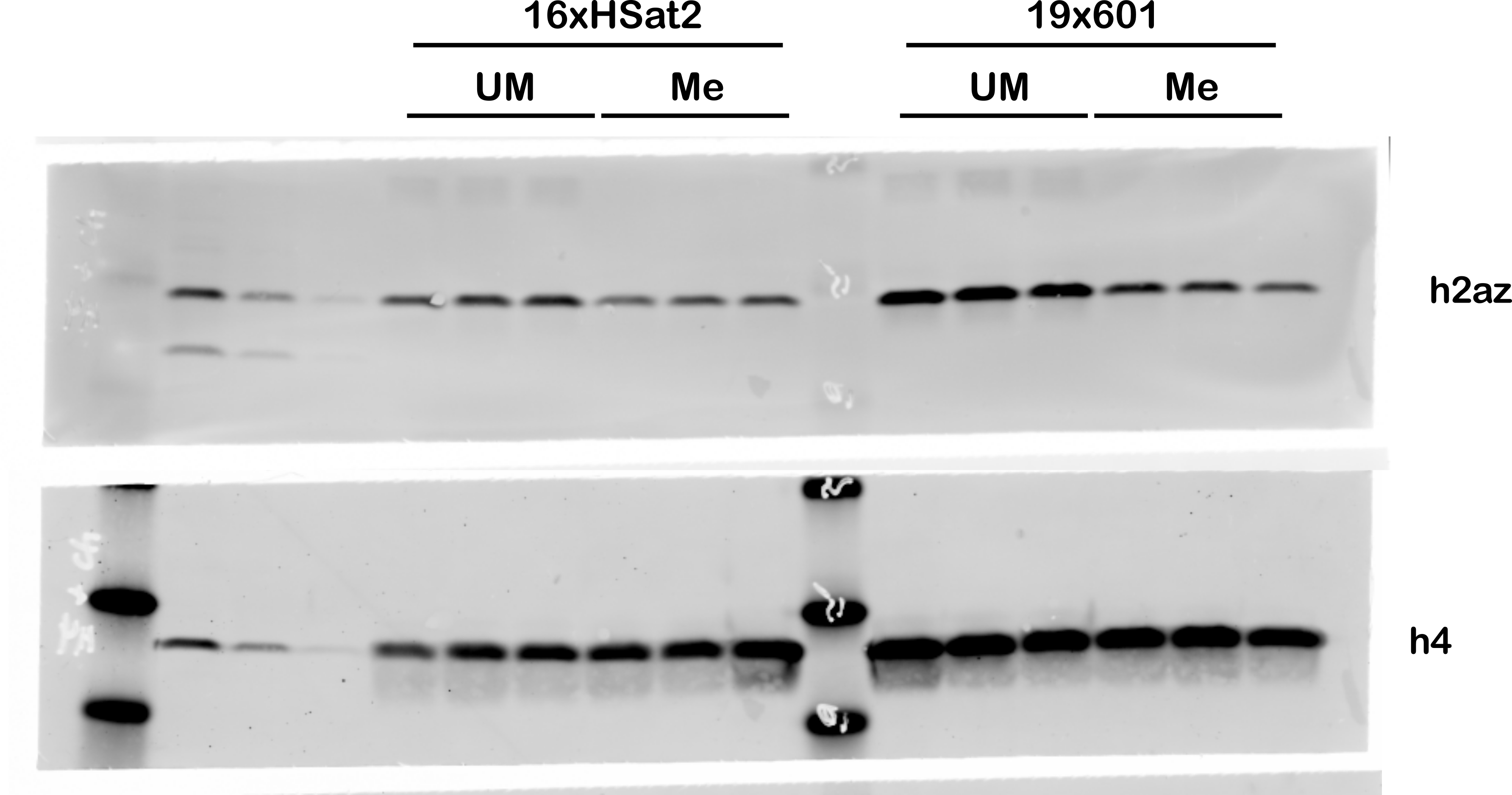

Supplement: Figure 4—source data 1. [file elife-109762-fig4-data1.zip › Figure 4 - source data 1/Figure 4 - source data 1 - 4C - H2AZ_H4 - LABELED.png]

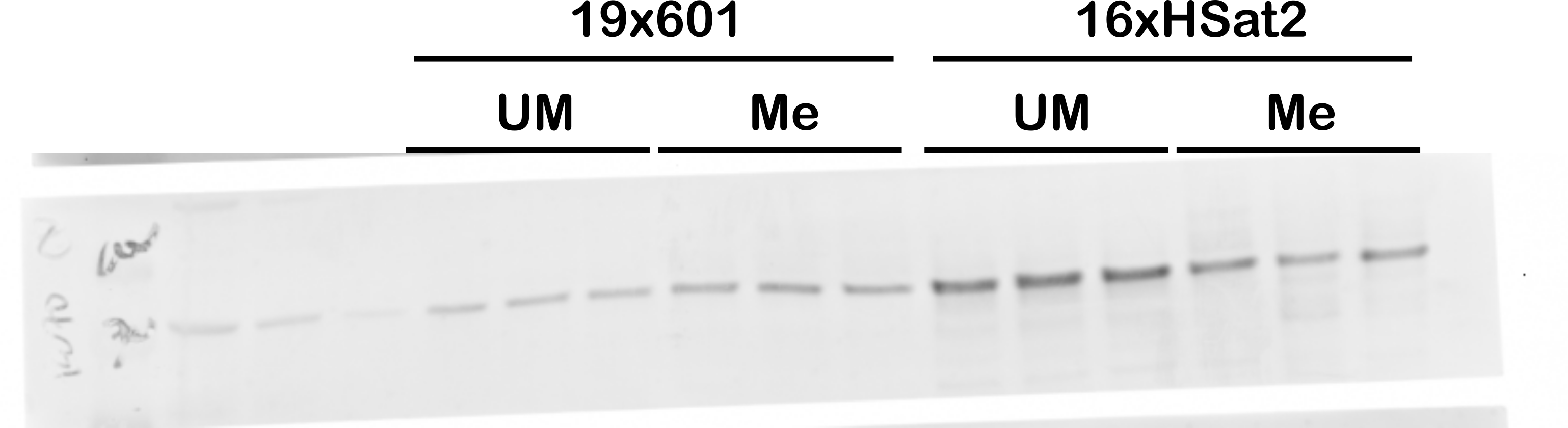

Supplement: Figure 4—figure supplement 3—source data 1. [file elife-109762-fig4-figsupp3-data1.zip › Figure 4 - figure supplement 3 - source data 1/Figure 4 - figure supplement 3 - source data 1 - 3B - Rep3 - Ku70onH2AZ - LABELED.png]

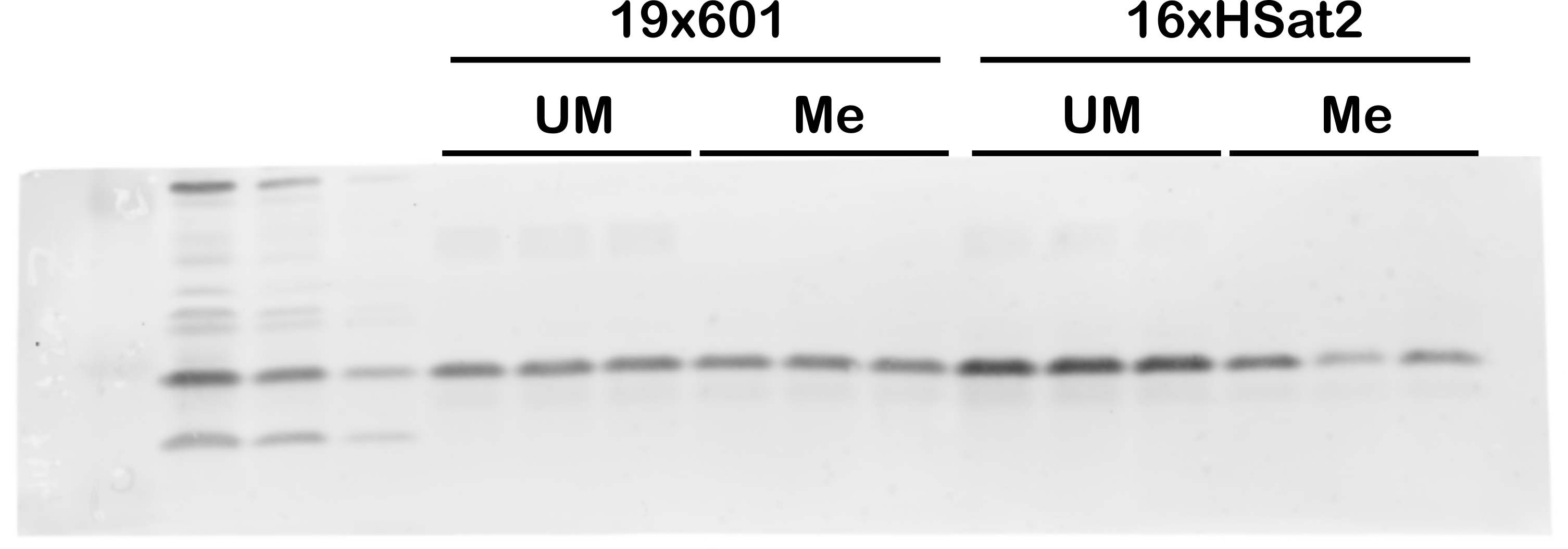

Supplement: Figure 4—figure supplement 3—source data 1. [file elife-109762-fig4-figsupp3-data1.zip › Figure 4 - figure supplement 3 - source data 1/Figure 4 - figure supplement 3 - source data 1 - 3B - Rep3 - H2AZ - LABELED.png]

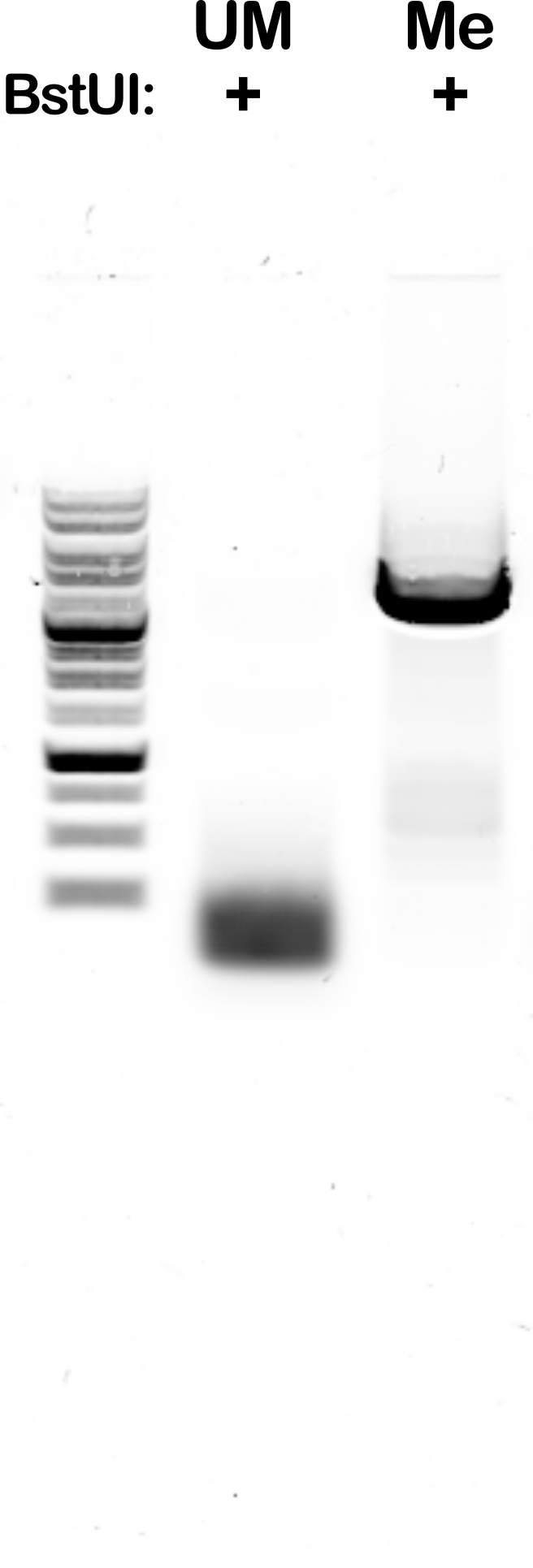

Supplement: Figure 4—figure supplement 3—source data 1. [file elife-109762-fig4-figsupp3-data1.zip › Figure 4 - figure supplement 3 - source data 1/Figure 4 - figure supplement 3 - source data 1 - 1A - LABELED.png]

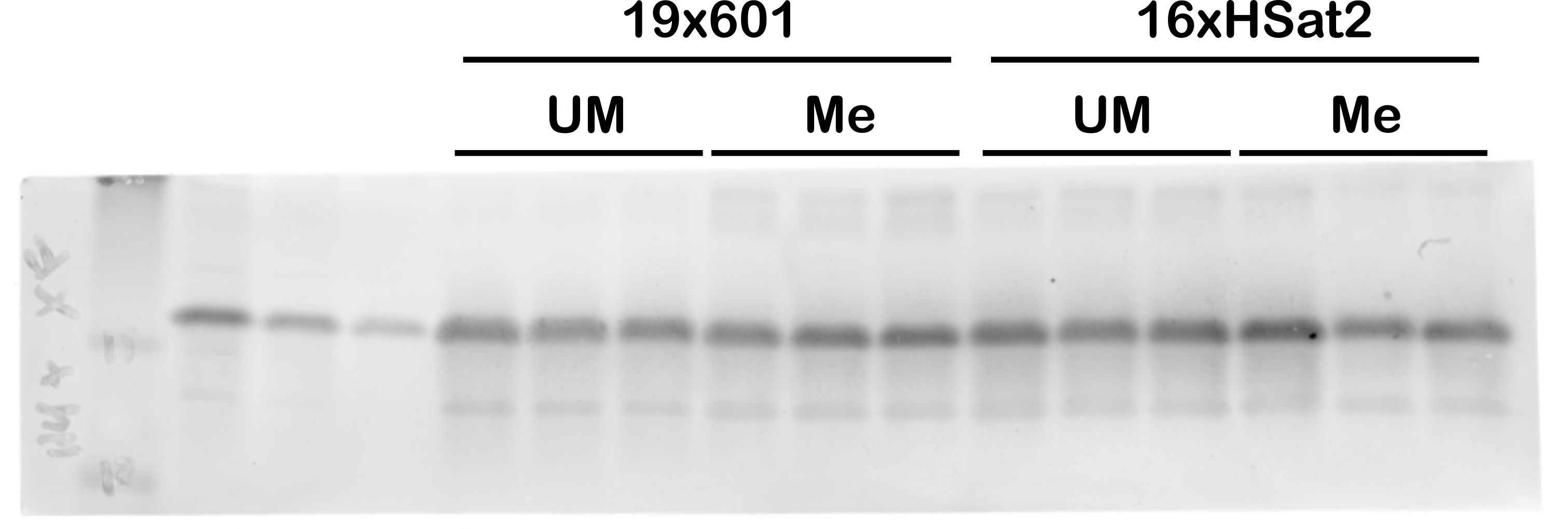

Supplement: Figure 4—figure supplement 3—source data 1. [file elife-109762-fig4-figsupp3-data1.zip › Figure 4 - figure supplement 3 - source data 1/Figure 4 - figure supplement 3 - source data 1 - 3B - Rep3 - H2AXF - LABELED.png]

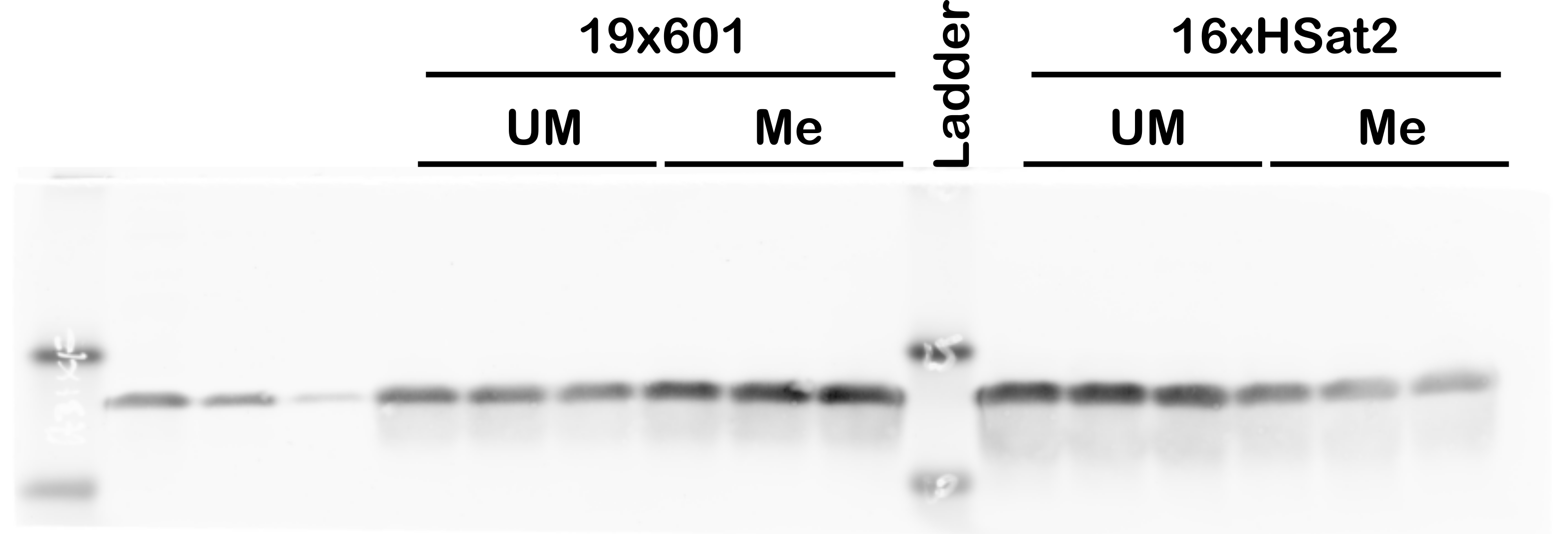

Supplement: Figure 4—figure supplement 3—source data 1. [file elife-109762-fig4-figsupp3-data1.zip › Figure 4 - figure supplement 3 - source data 1/Figure 4 - figure supplement 3 - source data 1 - 3B - Rep2 - H4onH2AZ - LABELED.png]

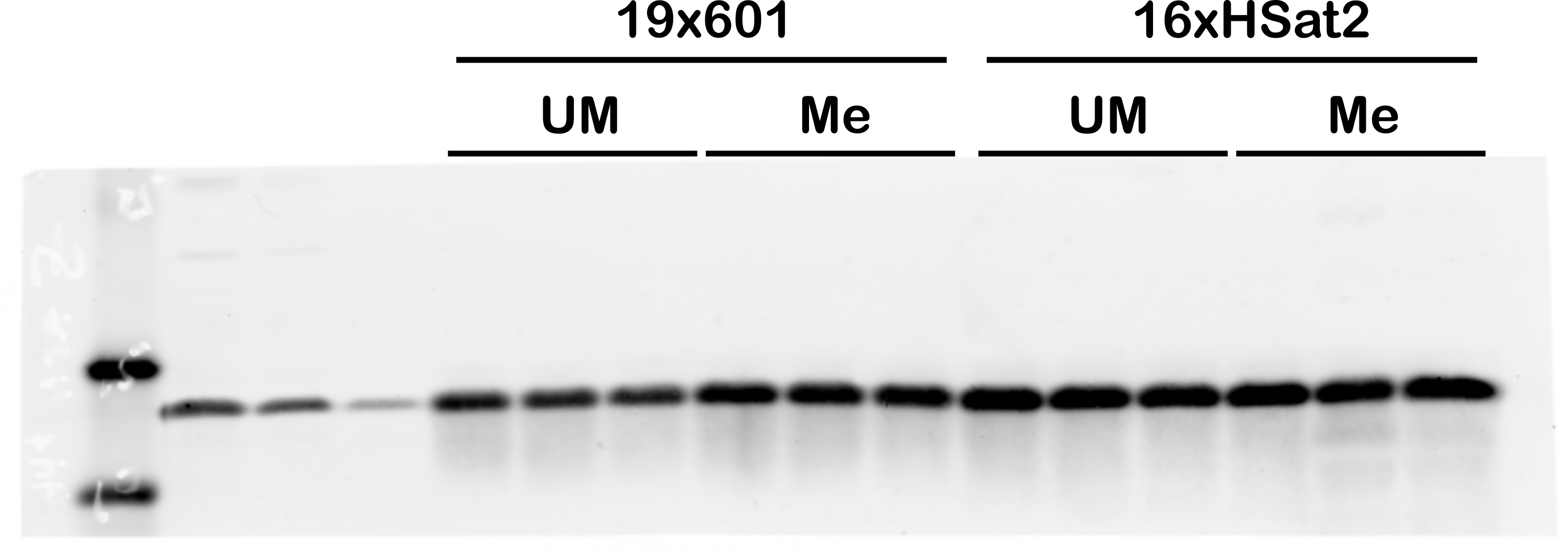

Supplement: Figure 4—figure supplement 3—source data 1. [file elife-109762-fig4-figsupp3-data1.zip › Figure 4 - figure supplement 3 - source data 1/Figure 4 - figure supplement 3 - source data 1 - 3B - Rep3 - H4onH2AZ - LABELED.png]

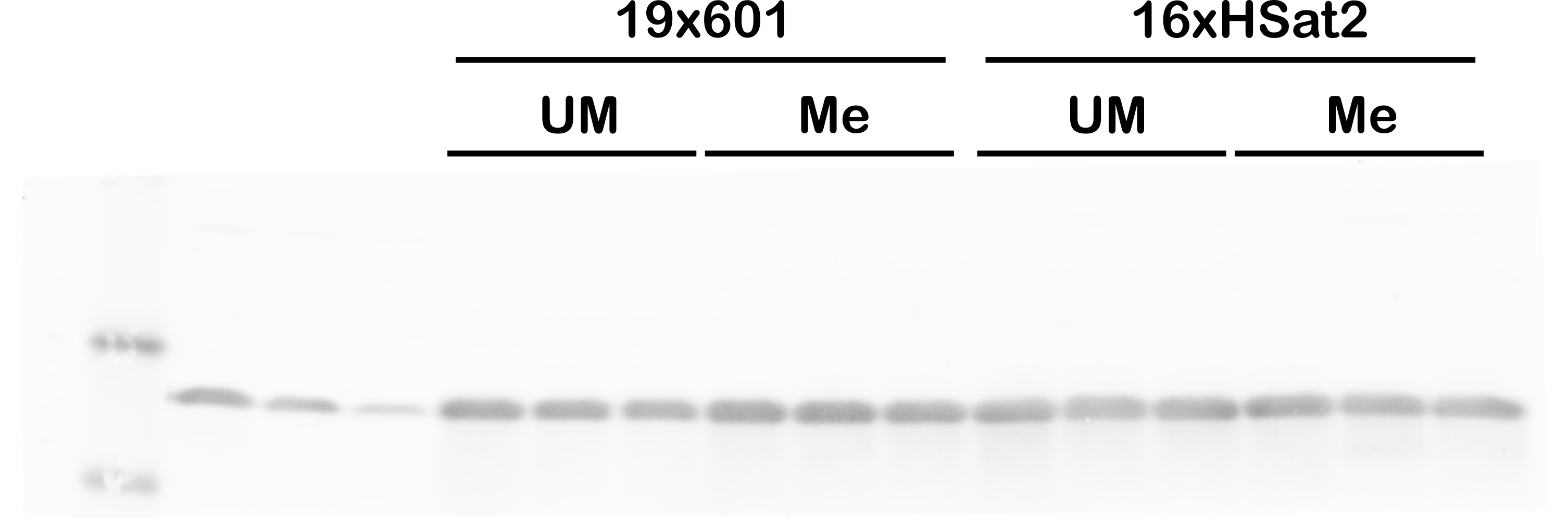

Supplement: Figure 4—figure supplement 3—source data 1. [file elife-109762-fig4-figsupp3-data1.zip › Figure 4 - figure supplement 3 - source data 1/Figure 4 - figure supplement 3 - source data 1 - 3B - Rep3 - H4onH2AXF - LABELED.png]

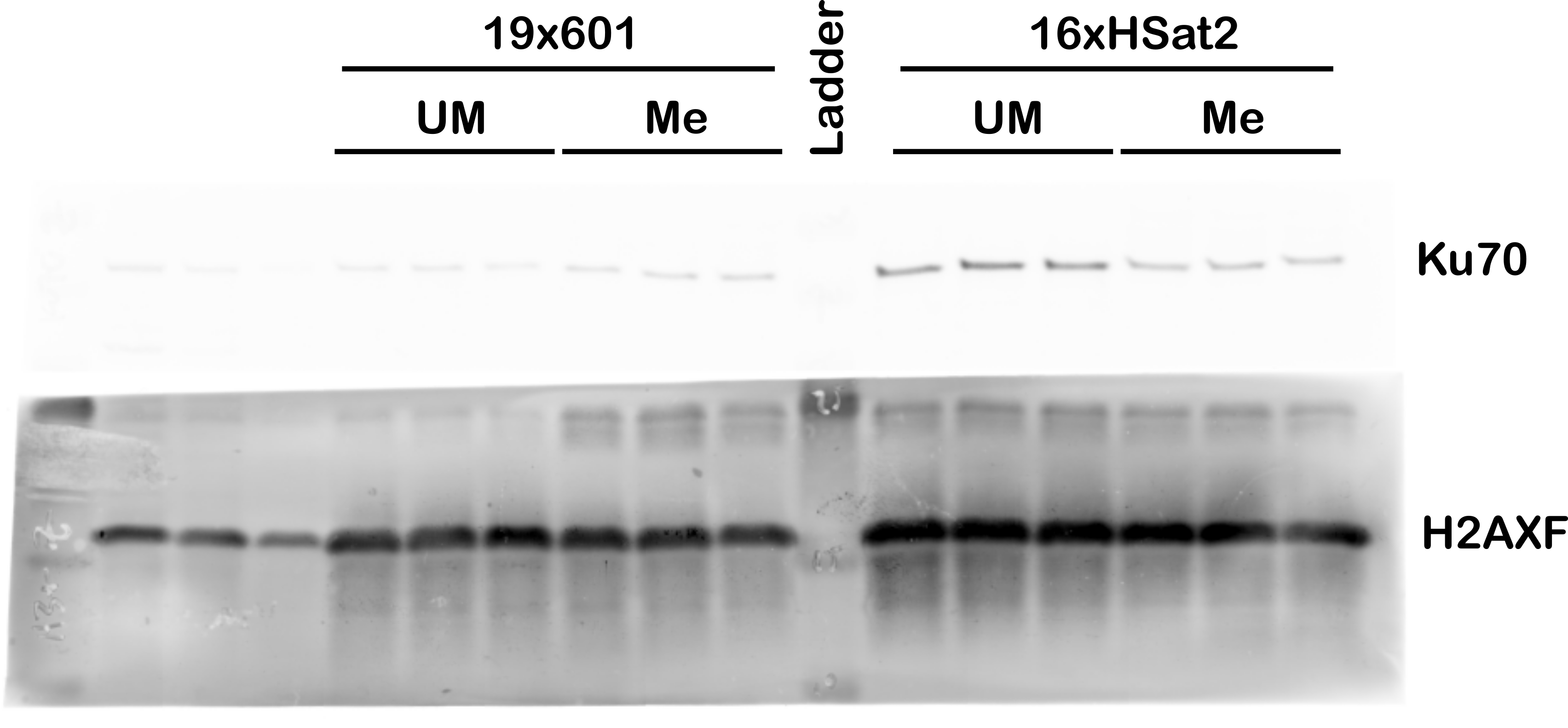

Supplement: Figure 4—figure supplement 3—source data 1. [file elife-109762-fig4-figsupp3-data1.zip › Figure 4 - figure supplement 3 - source data 1/Figure 4 - figure supplement 3 - source data 1 - 3B - Rep2 - H2AXF_Ku70 - LABELED.png]

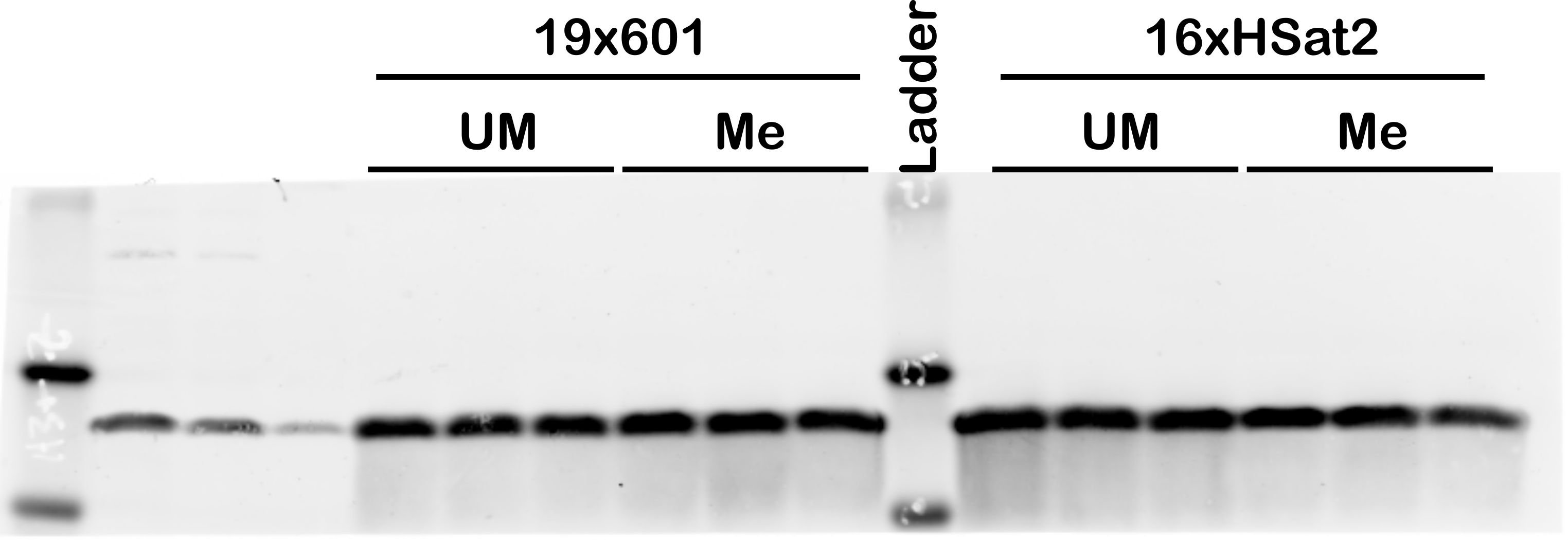

Supplement: Figure 4—figure supplement 3—source data 1. [file elife-109762-fig4-figsupp3-data1.zip › Figure 4 - figure supplement 3 - source data 1/Figure 4 - figure supplement 3 - source data 1 - 3B - Rep2 - H4onH2AXF - LABELED.png]

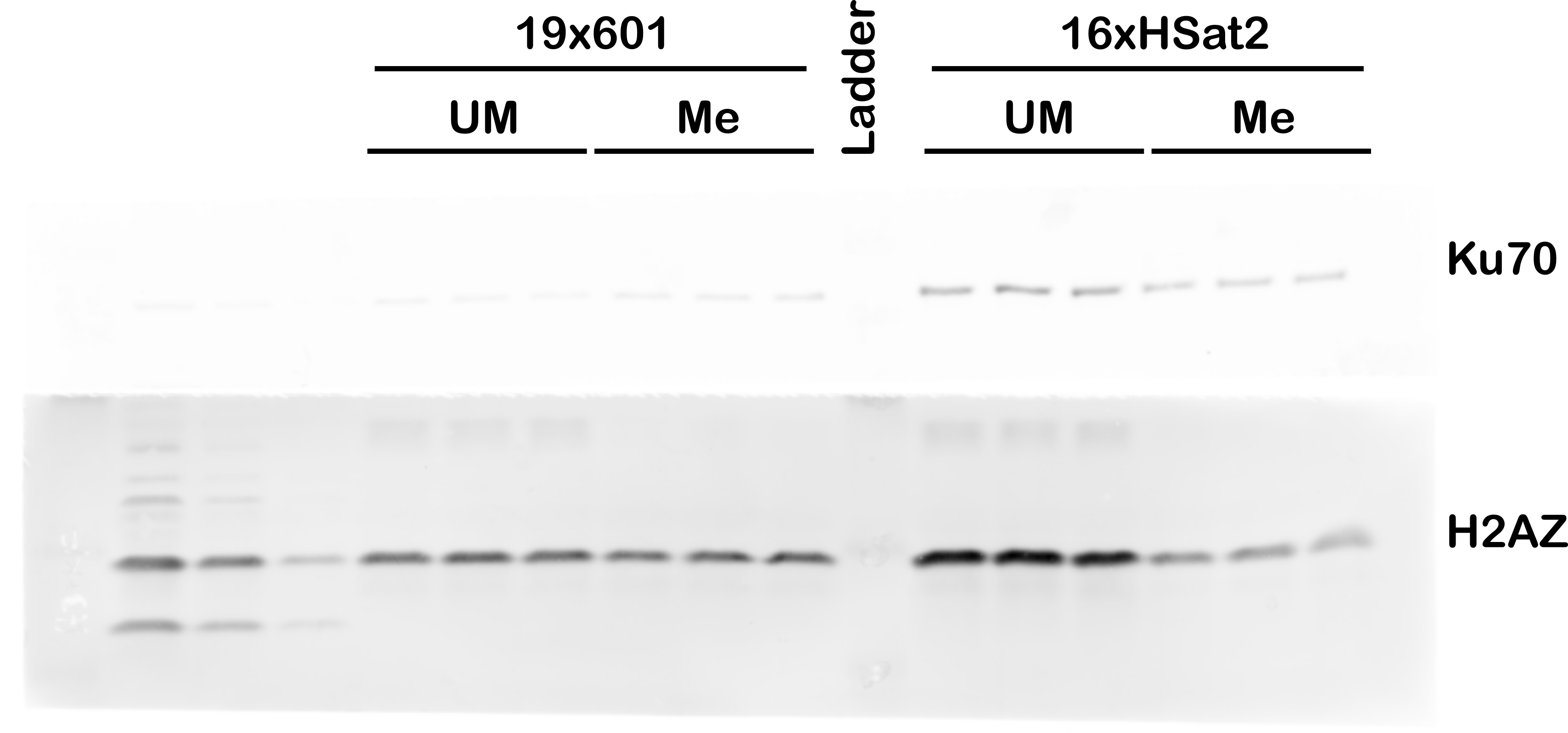

Supplement: Figure 4—figure supplement 3—source data 1. [file elife-109762-fig4-figsupp3-data1.zip › Figure 4 - figure supplement 3 - source data 1/Figure 4 - figure supplement 3 - source data 1 - 3B - Rep2 - H2AZ_Ku70 - LABELED.png]

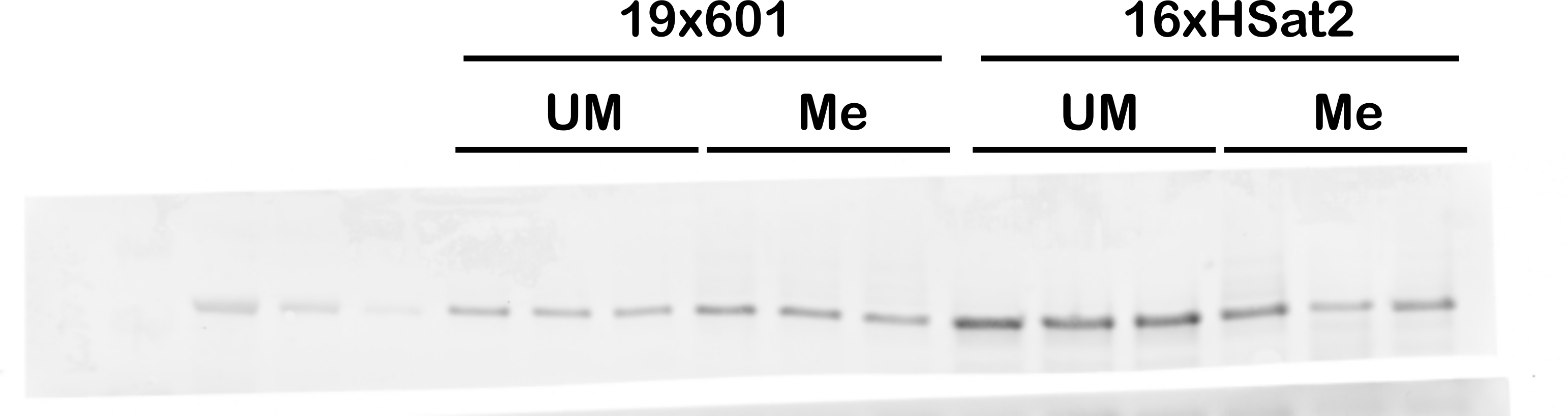

Supplement: Figure 4—figure supplement 3—source data 1. [file elife-109762-fig4-figsupp3-data1.zip › Figure 4 - figure supplement 3 - source data 1/Figure 4 - figure supplement 3 - source data 1 - 3B - Rep3 - Ku70onH2AXF - LABELED.png]

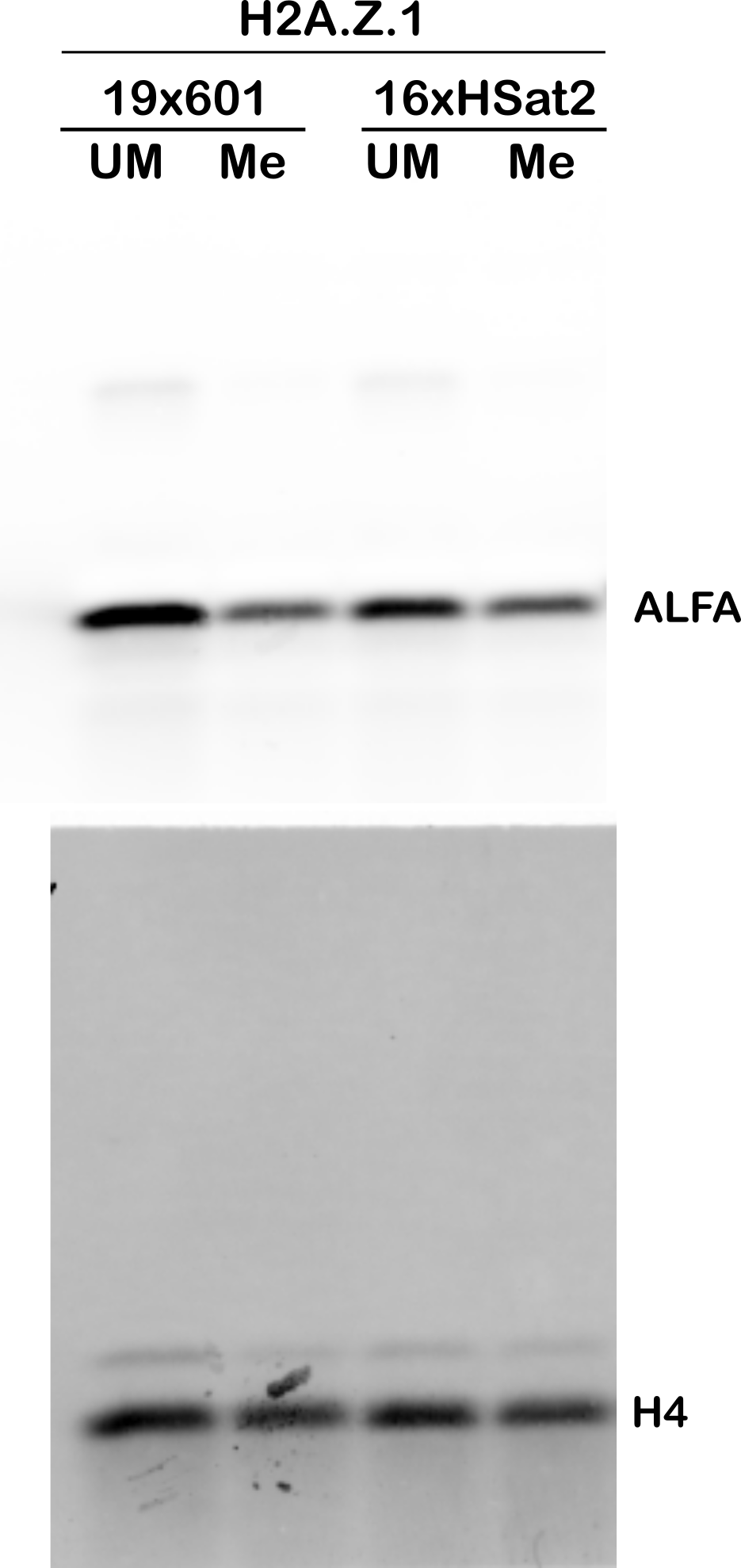

Supplement: Figure 4—figure supplement 4—source data 1. [file elife-109762-fig4-figsupp4-data1.zip › Figure 4 - figure supplement 4 - source data 1/Figure 4 - figure supplement 4 - source data 1 - 4B - LABELED.png]

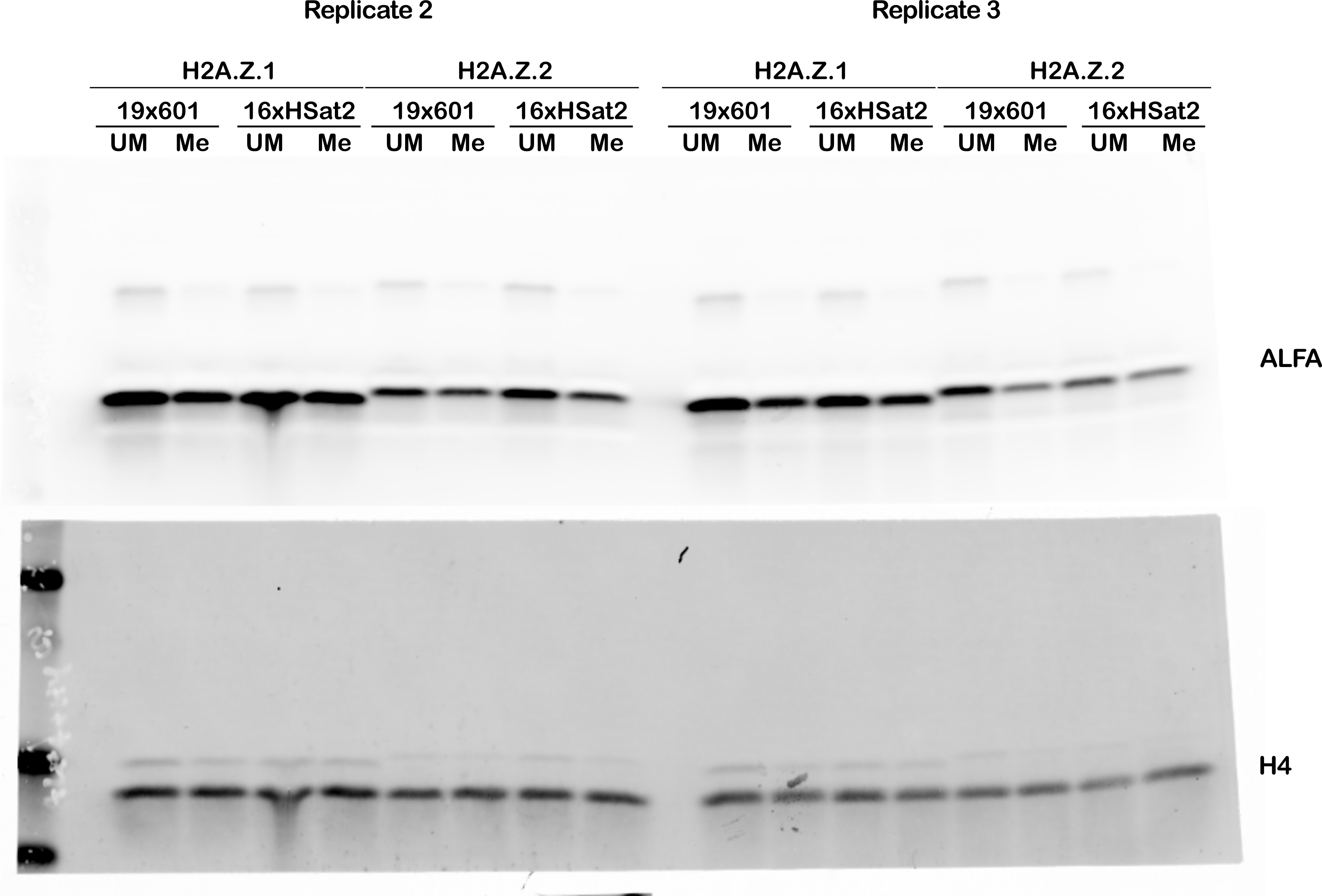

Supplement: Figure 4—figure supplement 4—source data 1. [file elife-109762-fig4-figsupp4-data1.zip › Figure 4 - figure supplement 4 - source data 1/Figure 4 - figure supplement 4 - source data 1 - 4E - LABELED.png]

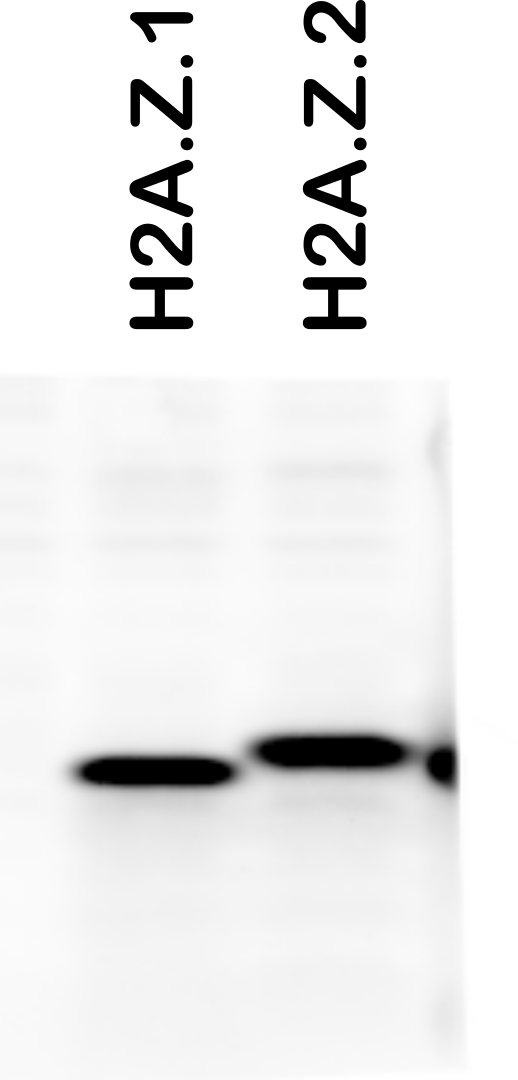

Supplement: Figure 4—figure supplement 4—source data 1. [file elife-109762-fig4-figsupp4-data1.zip › Figure 4 - figure supplement 4 - source data 1/Figure 4 - figure supplement 4 - source data 1 - 4A - LABELED.png]

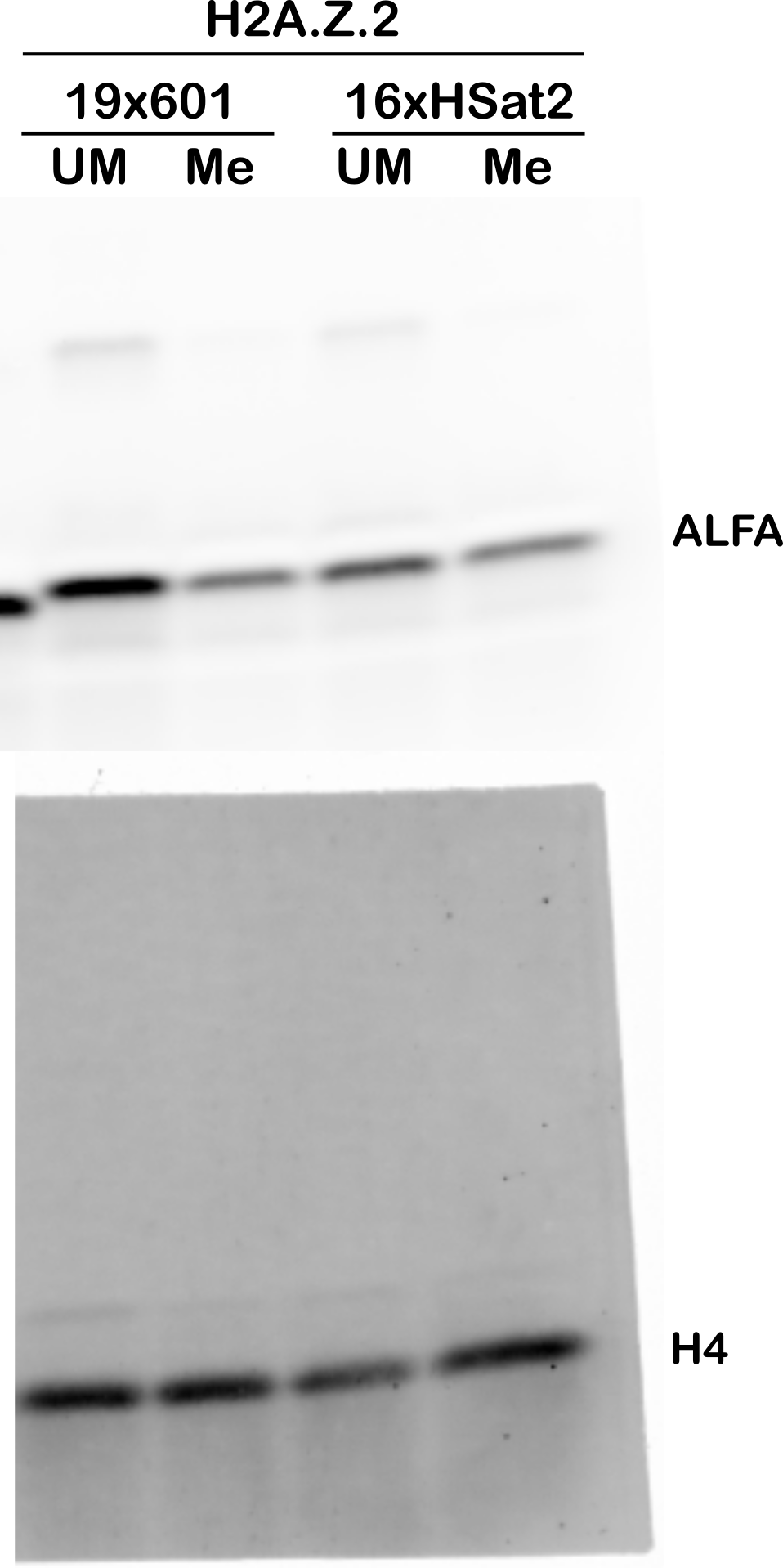

Supplement: Figure 4—figure supplement 4—source data 1. [file elife-109762-fig4-figsupp4-data1.zip › Figure 4 - figure supplement 4 - source data 1/Figure 4 - figure supplement 4 - source data 1 - 4C - LABELED.png]

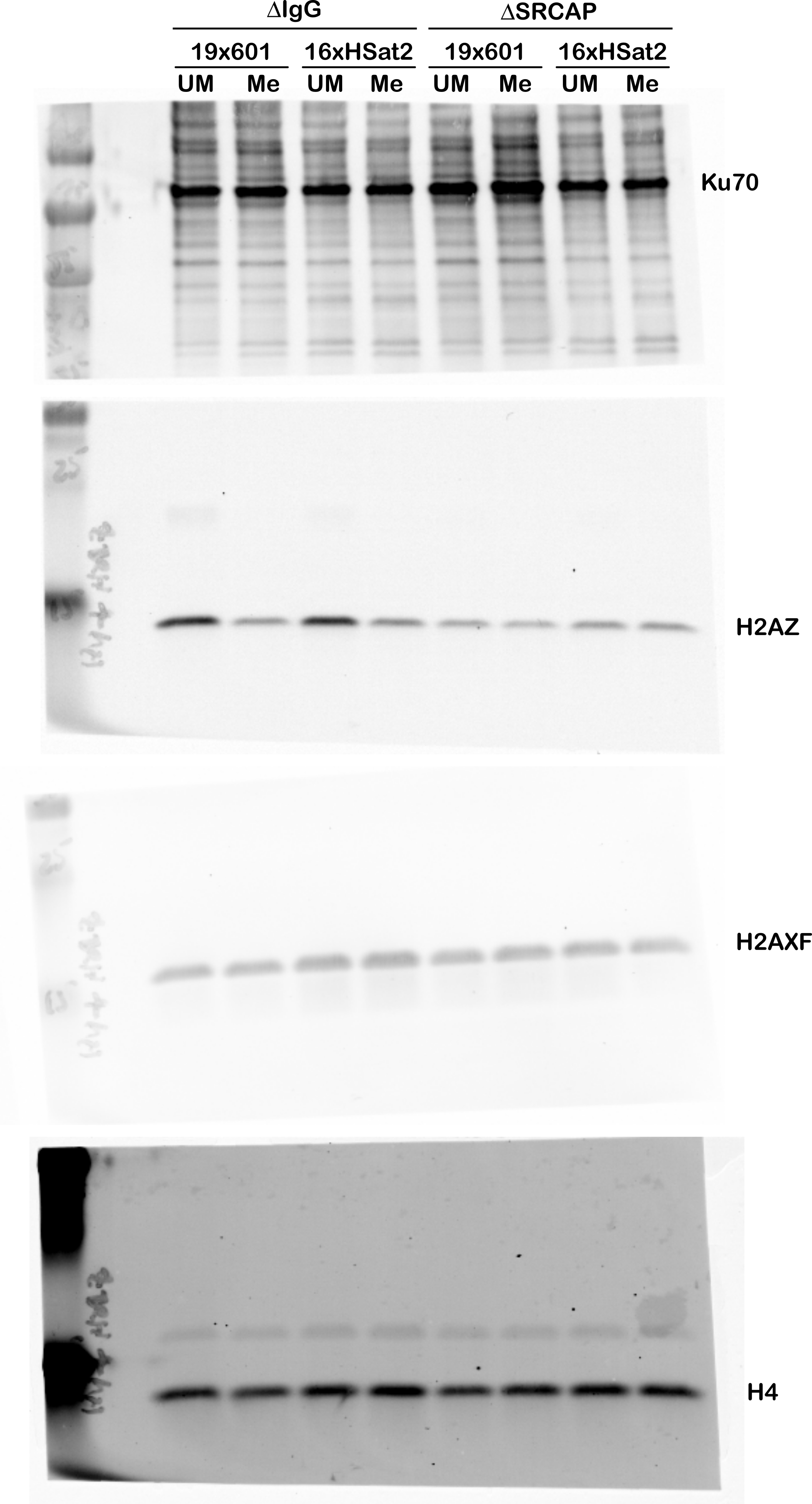

Supplement: Figure 5—source data 1. [file elife-109762-fig5-data1.zip › Figure 5 - source data 1/Figure 5 - source data 1 - 5B - LABELED.png]

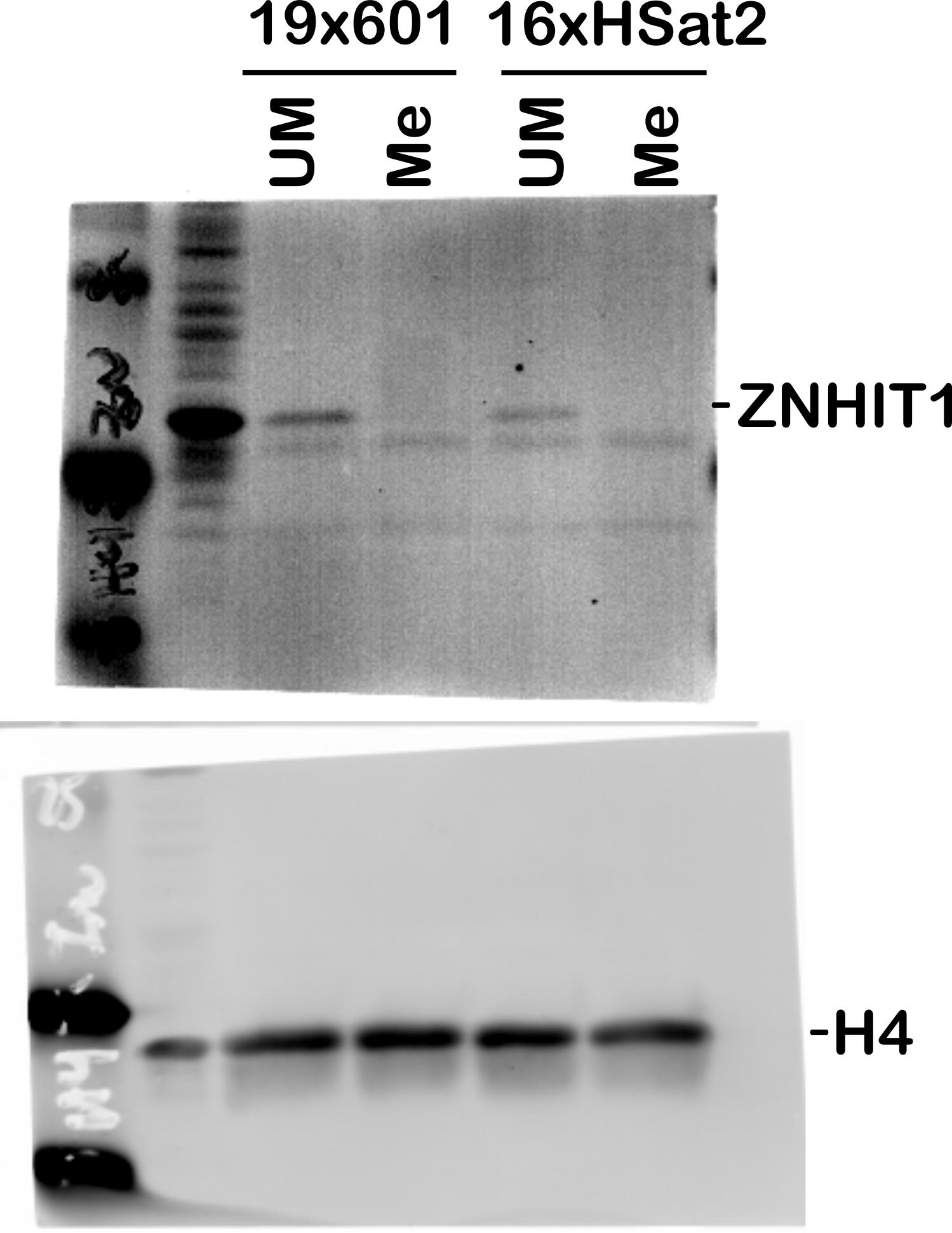

Supplement: Figure 5—source data 1. [file elife-109762-fig5-data1.zip › Figure 5 - source data 1/Figure 5 - source data 1 - 5F - LABELED.png]

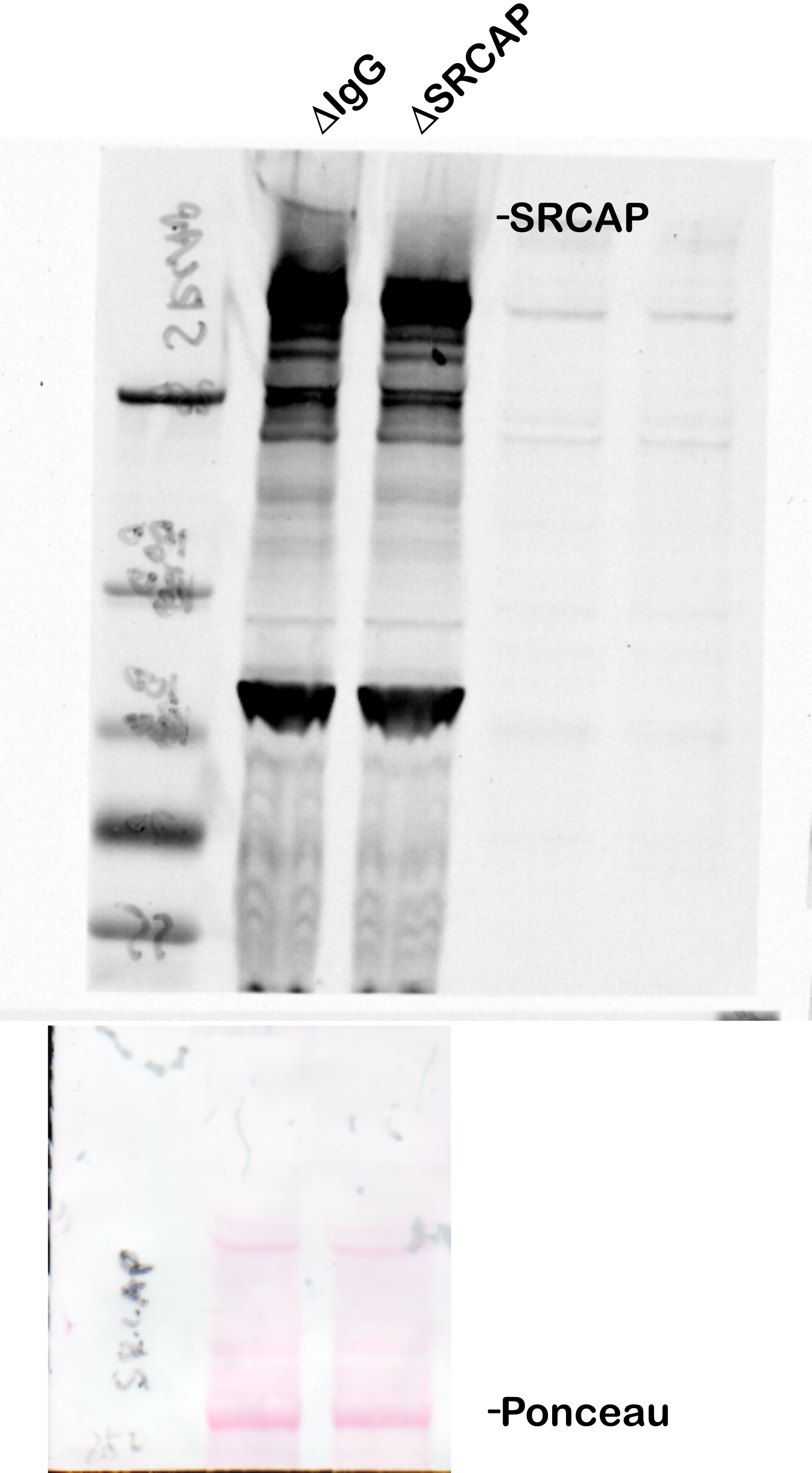

Supplement: Figure 5—source data 1. [file elife-109762-fig5-data1.zip › Figure 5 - source data 1/Figure 5 - source data 1 - 5A - LABELED.png]

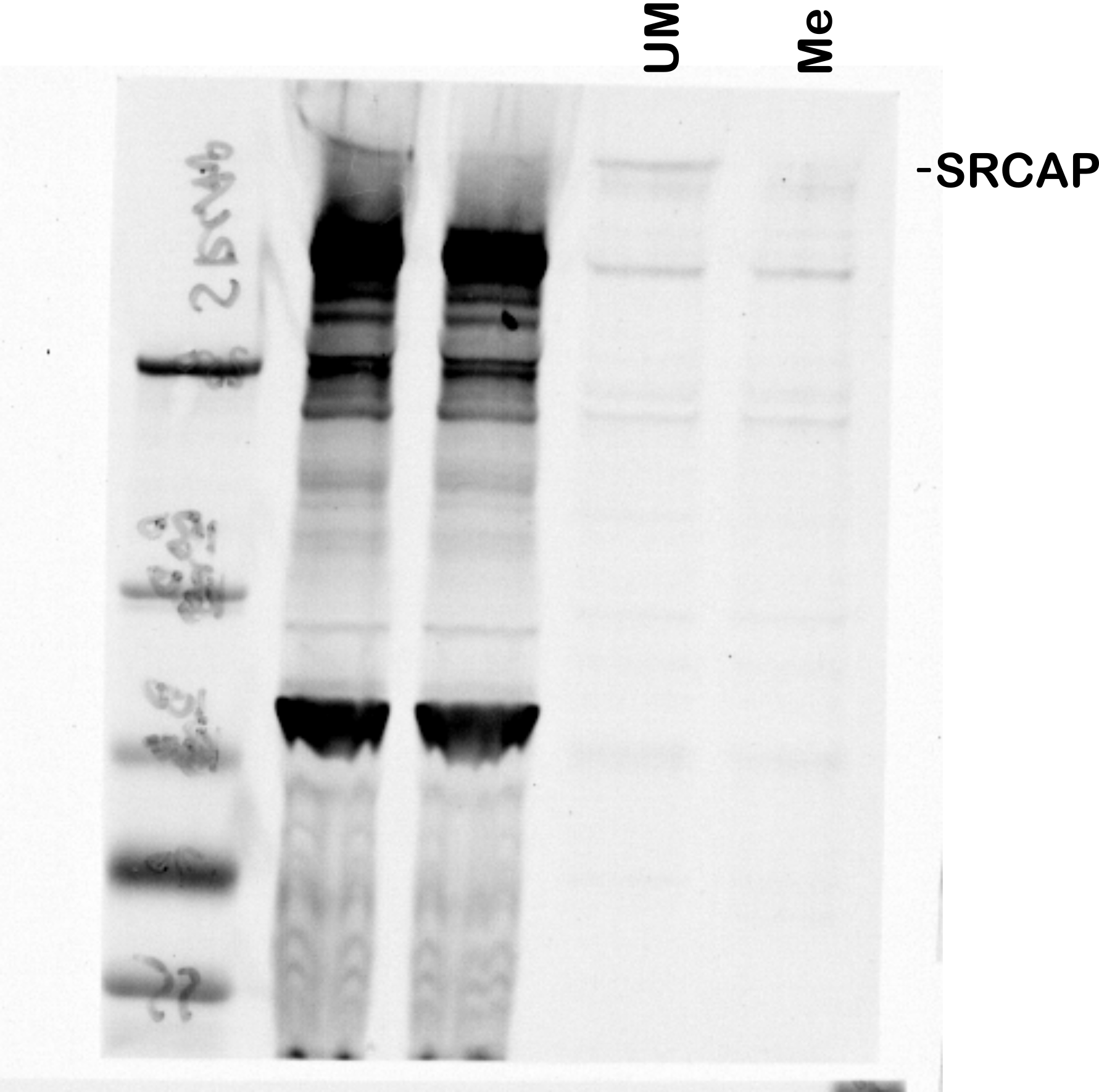

Supplement: Figure 5—source data 1. [file elife-109762-fig5-data1.zip › Figure 5 - source data 1/Figure 5 - source data 1 - 5E - LABELED.png]

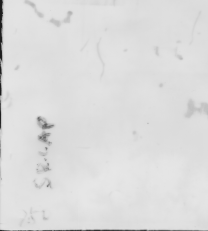

Supplement: Figure 5—source data 2. [file elife-109762-fig5-data2.zip › Figure 5 - source data 2/Figure 5 - source data 2 - 5A - ponceau - RAW.tif]

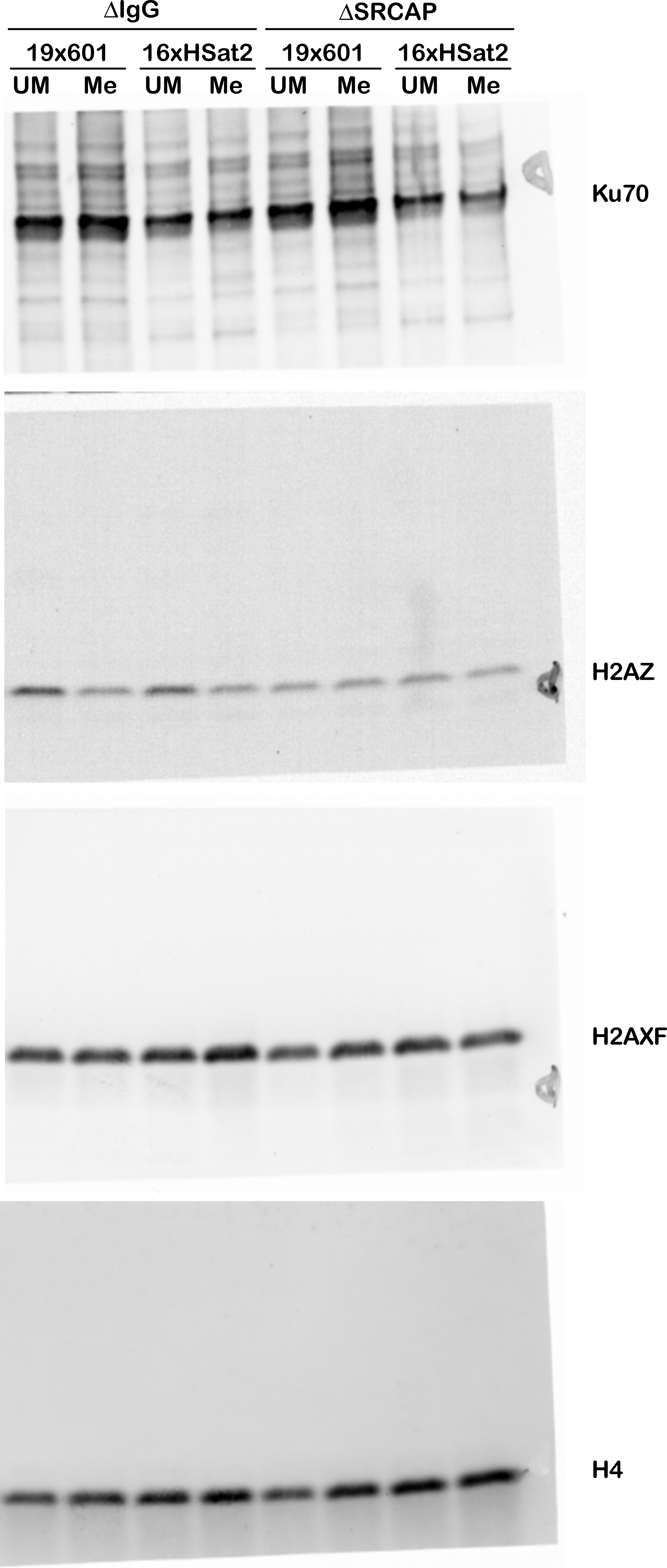

Supplement: Figure 5—figure supplement 1—source data 1. [file elife-109762-fig5-figsupp1-data1.zip › Figure 5 - figure supplement 1 - source data 1/Figure 5 - figure supplement 1 - source data 1 - 1C - Rep3 - LABELED.png]

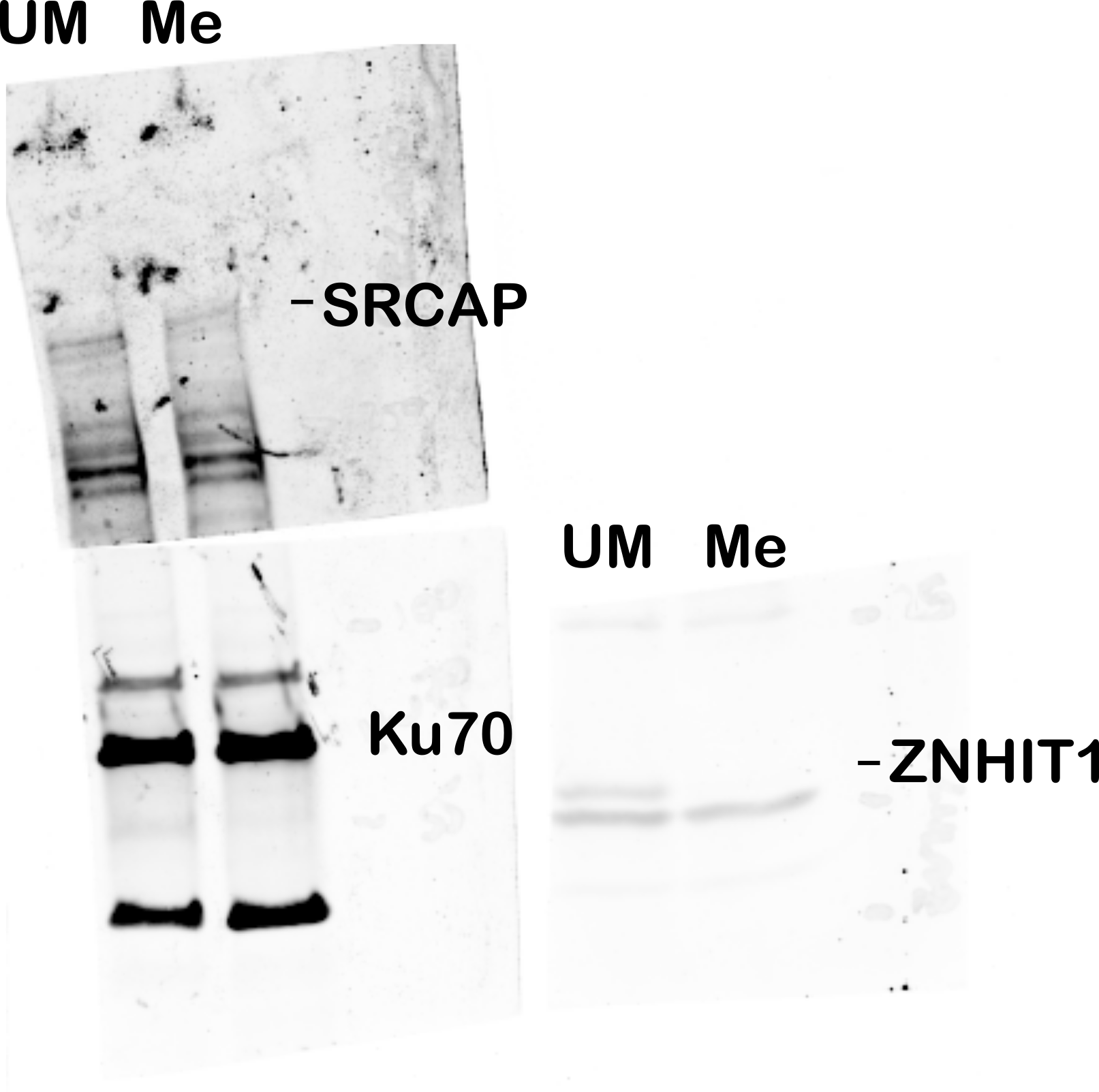

Supplement: Figure 5—figure supplement 1—source data 1. [file elife-109762-fig5-figsupp1-data1.zip › Figure 5 - figure supplement 1 - source data 1/Figure 5 - figure supplement 1 - source data 1 - 1D - LABELED.png]

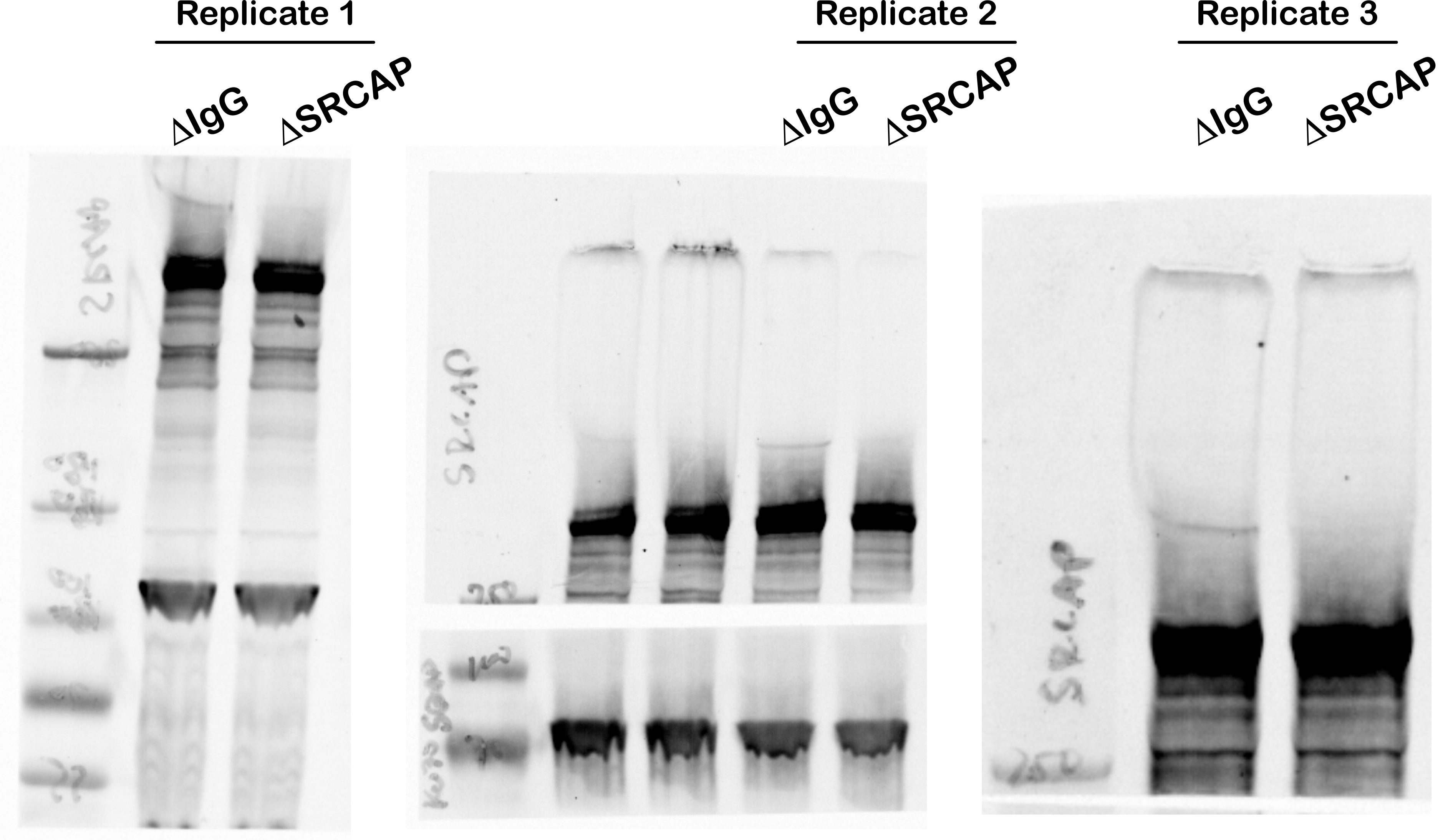

Supplement: Figure 5—figure supplement 1—source data 1. [file elife-109762-fig5-figsupp1-data1.zip › Figure 5 - figure supplement 1 - source data 1/Figure 5 - figure supplement 1 - source data 1 - 1B - LABELED.png]

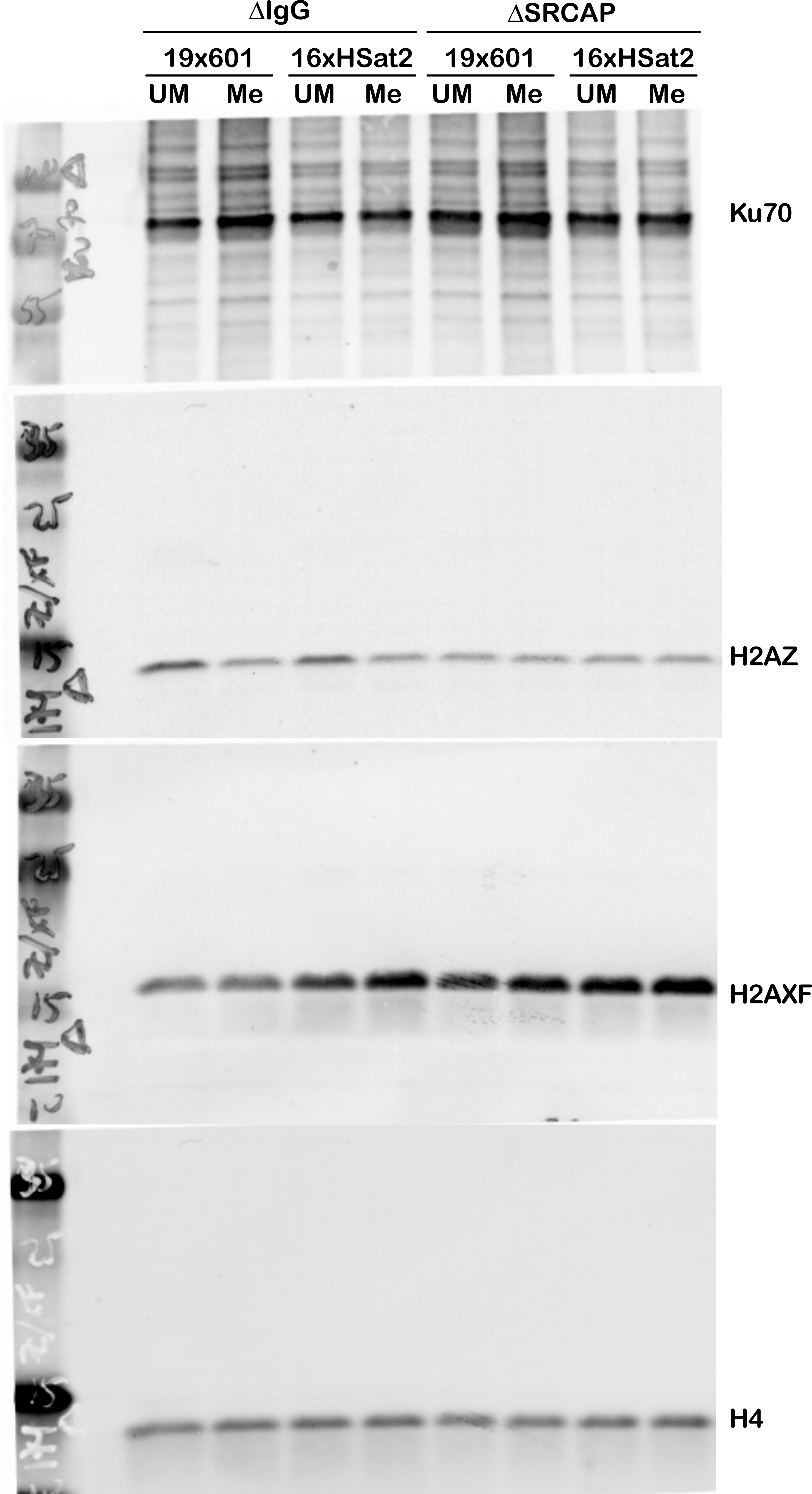

Supplement: Figure 5—figure supplement 1—source data 1. [file elife-109762-fig5-figsupp1-data1.zip › Figure 5 - figure supplement 1 - source data 1/Figure 5 - figure supplement 1 - source data 1 - 1C - Rep2 - LABELED.png]

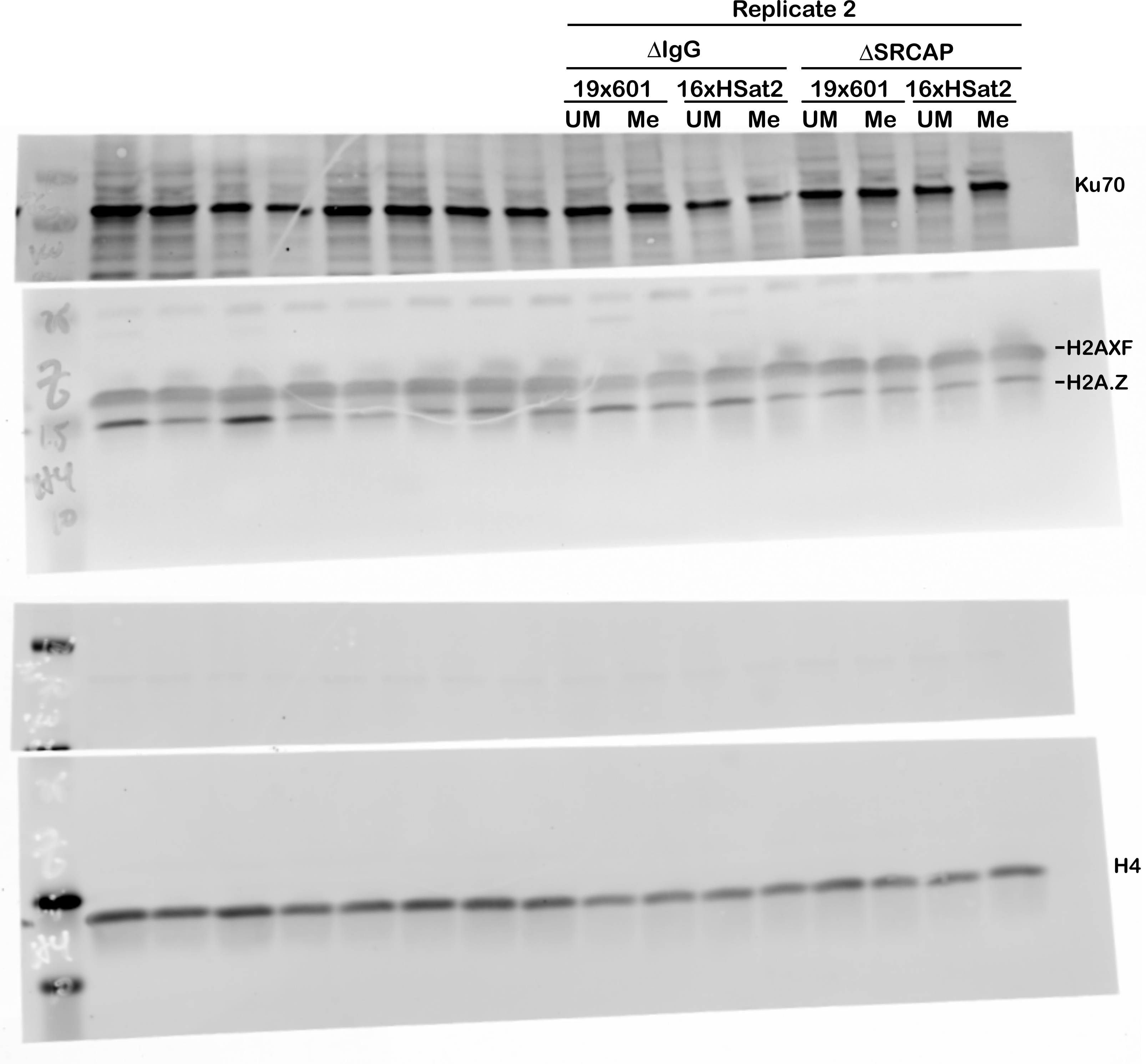

Supplement: Figure 5—figure supplement 2—source data 1. [file elife-109762-fig5-figsupp2-data1.zip › Figure 5 - figure supplement 2 - source data 1/Figure 5 - figure supplement 2 - source data 1 - 2E - Rep2 - LABELED.png]

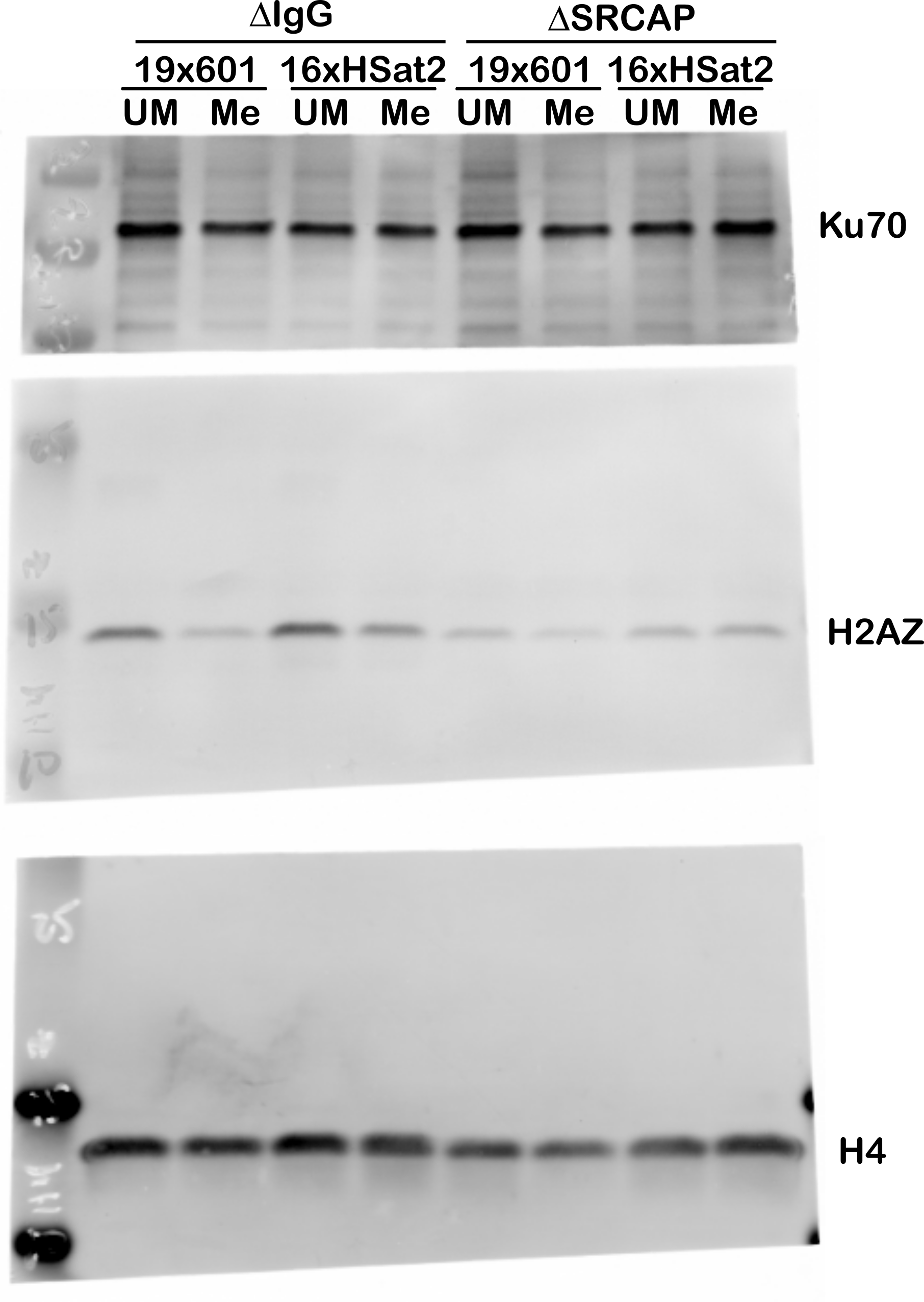

Supplement: Figure 5—figure supplement 2—source data 1. [file elife-109762-fig5-figsupp2-data1.zip › Figure 5 - figure supplement 2 - source data 1/Figure 5 - figure supplement 2 - source data 1 - 2A - LABELED.png]

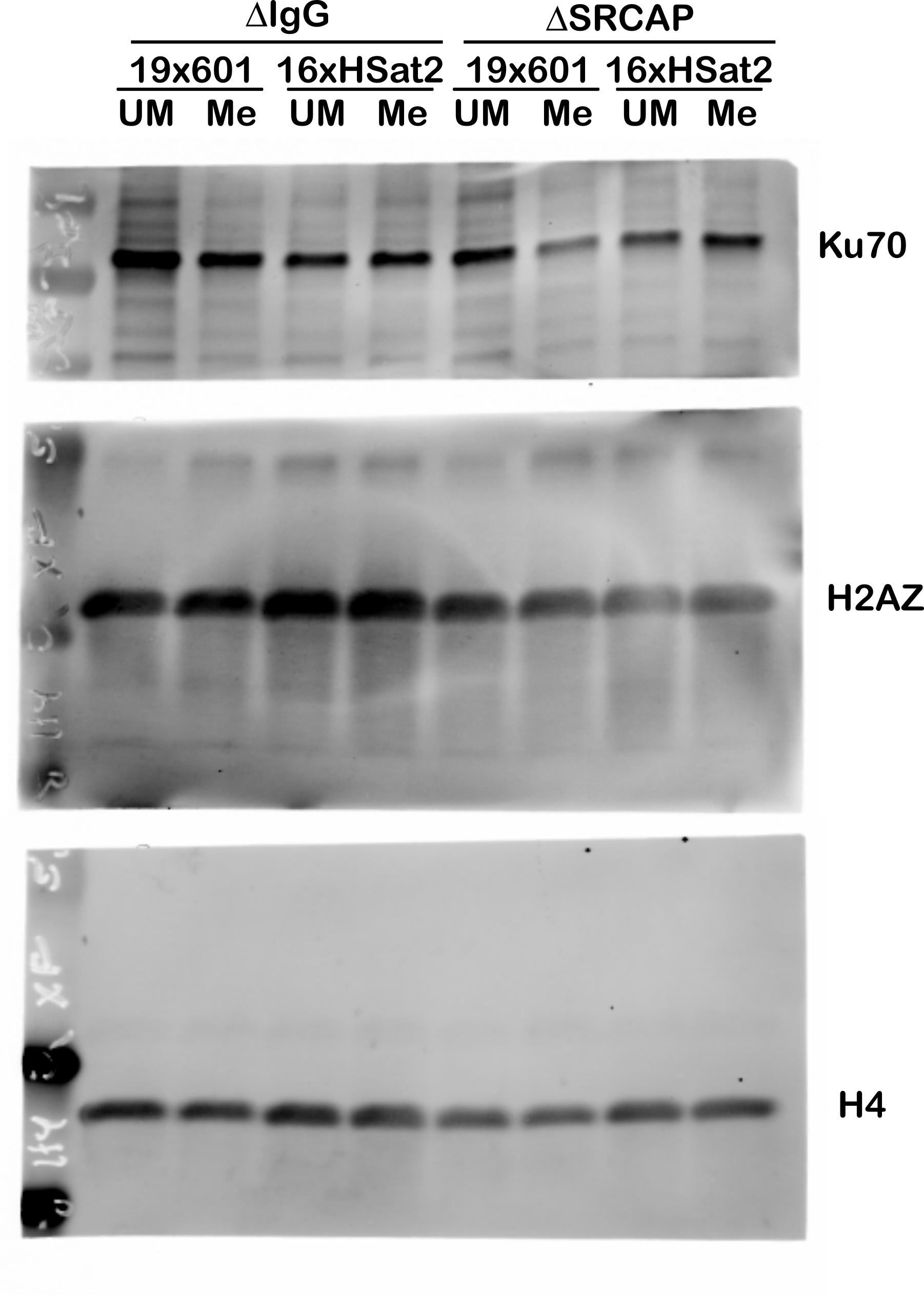

Supplement: Figure 5—figure supplement 2—source data 1. [file elife-109762-fig5-figsupp2-data1.zip › Figure 5 - figure supplement 2 - source data 1/Figure 5 - figure supplement 2 - source data 1 - 2C - LABELED.png]

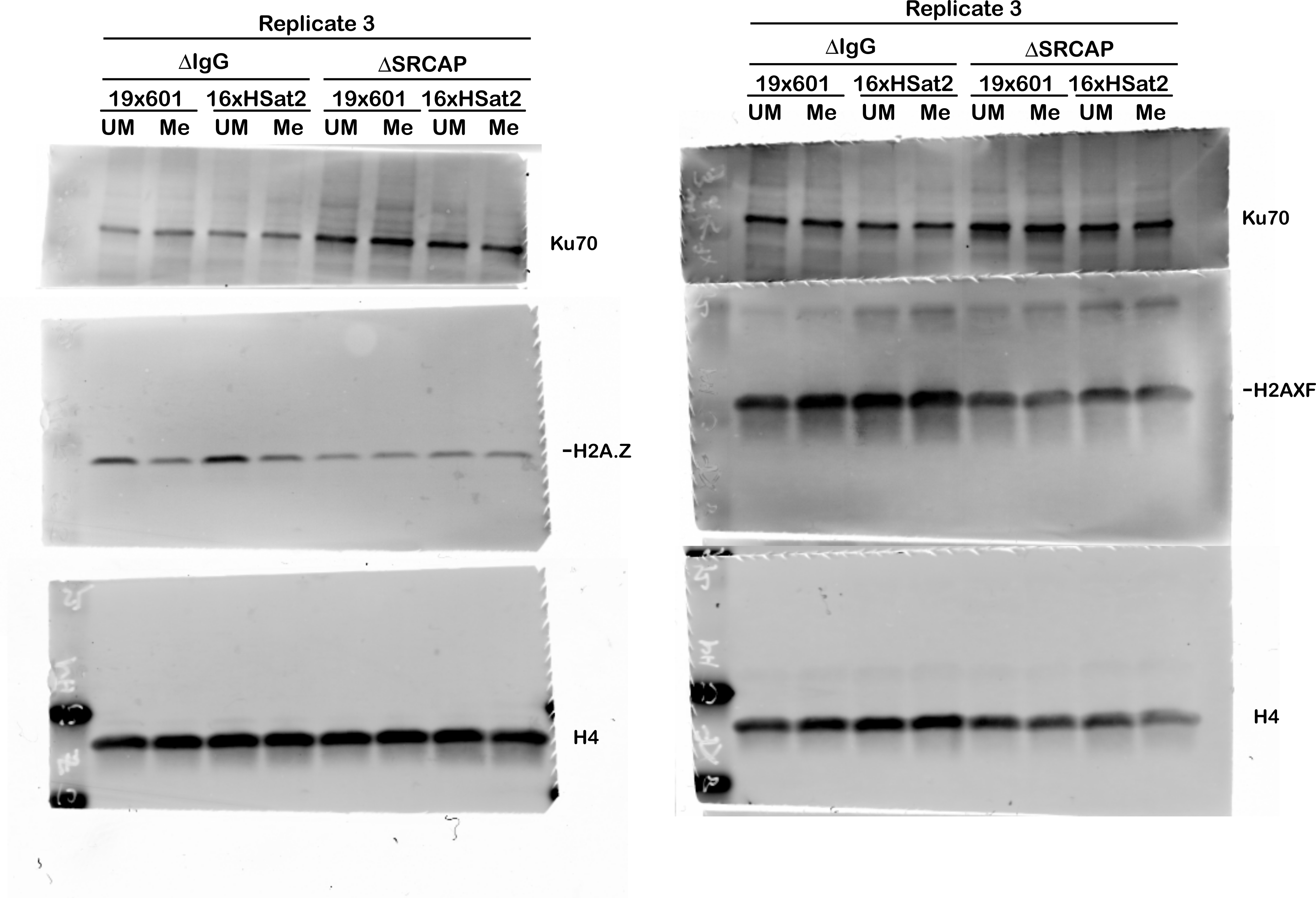

Supplement: Figure 5—figure supplement 2—source data 1. [file elife-109762-fig5-figsupp2-data1.zip › Figure 5 - figure supplement 2 - source data 1/Figure 5 - figure supplement 2 - source data 1 - 2E - Rep3 - LABELED.png]

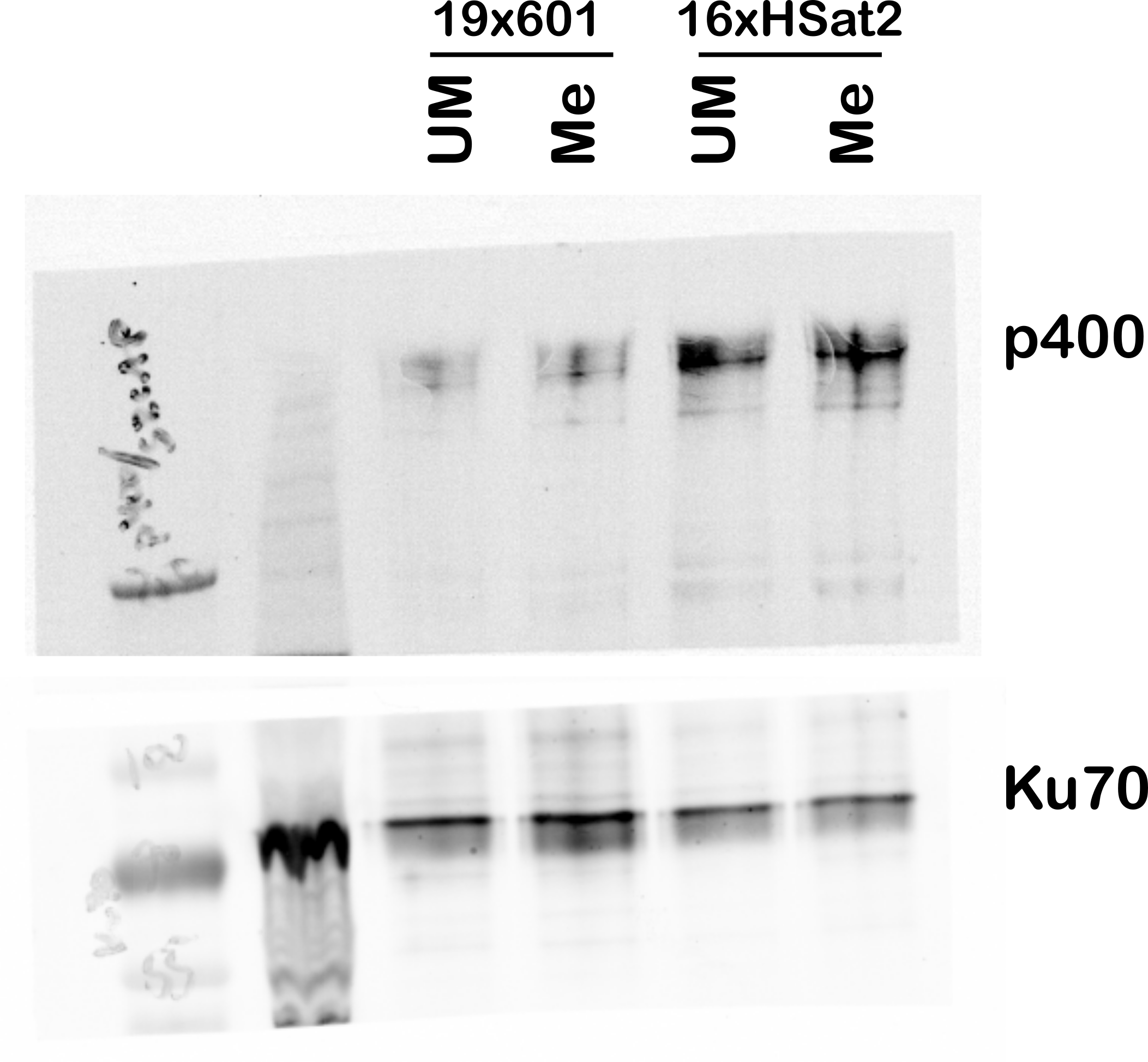

Supplement: Figure 5—figure supplement 3—source data 1. [file elife-109762-fig5-figsupp3-data1.zip › Figure 5 - figure supplement 3 - source data 1/Figure 5 - figure supplement 3 - source data 1 - LABELED.png]

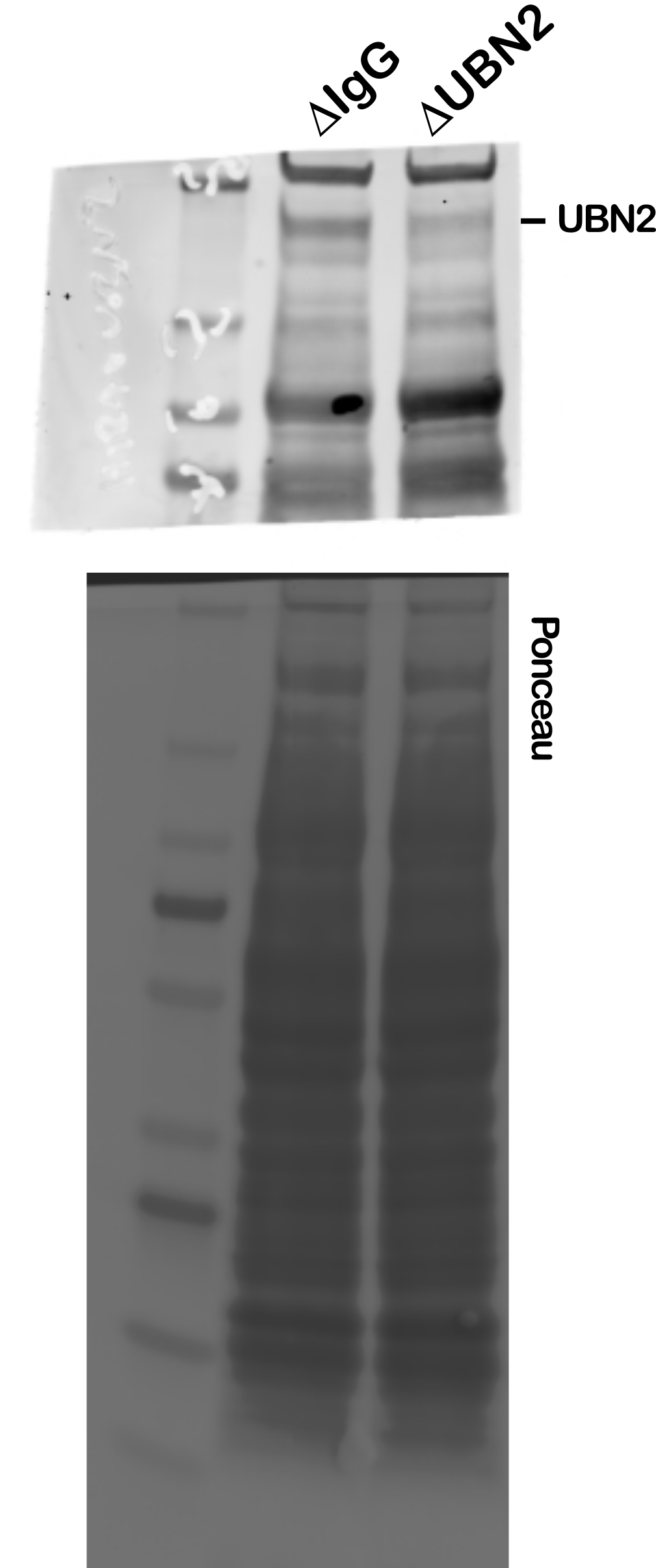

Supplement: Figure 5—figure supplement 4—source data 1. [file elife-109762-fig5-figsupp4-data1.zip › Figure 5 - figure supplement 4 - source data 1/Figure 5 - figure supplement 4 - source data 1 - 4A - LABELED.png]

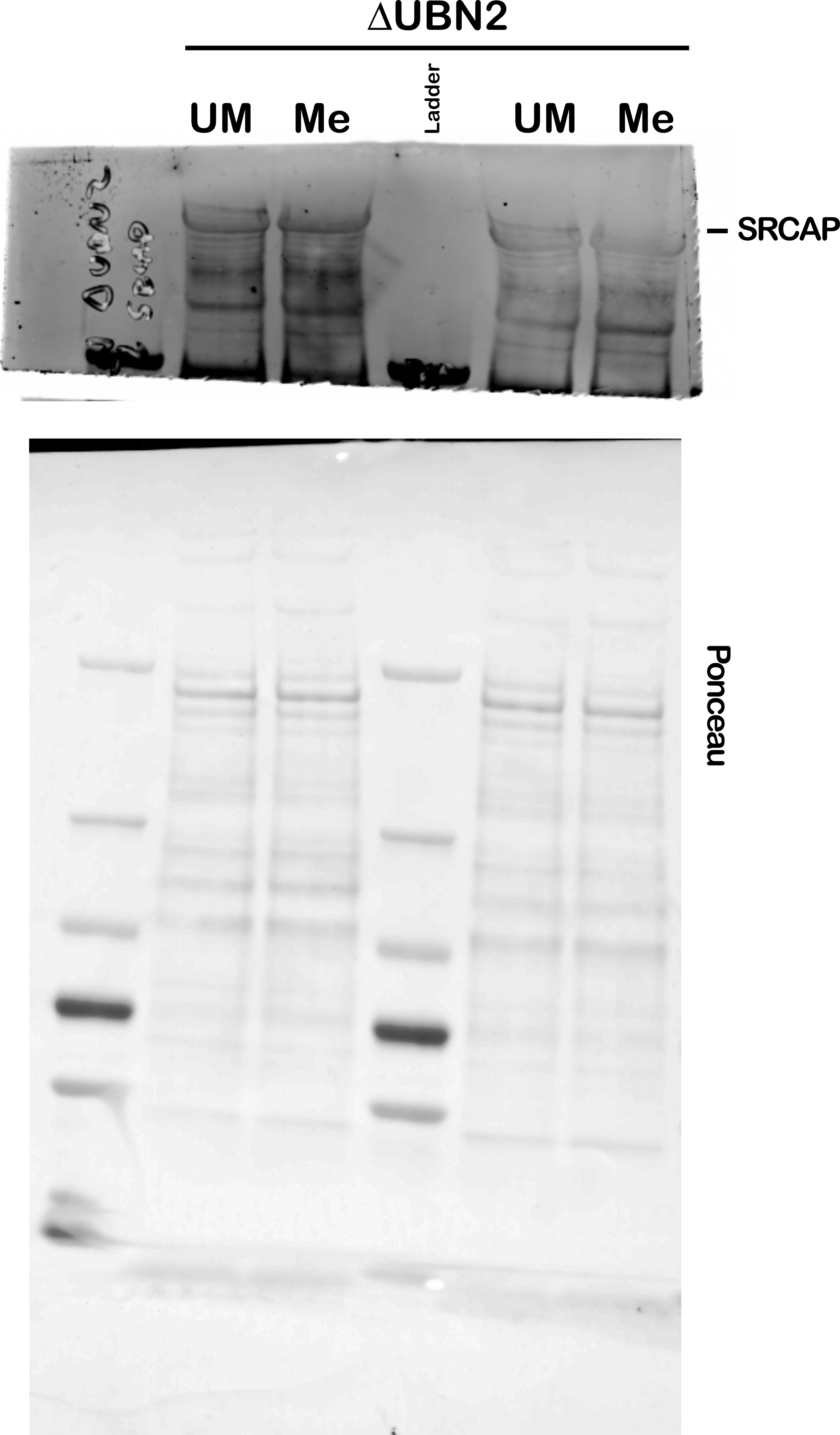

Supplement: Figure 5—figure supplement 4—source data 1. [file elife-109762-fig5-figsupp4-data1.zip › Figure 5 - figure supplement 4 - source data 1/Figure 5 - figure supplement 4 - source data 1 - 4B - LABELED.png]

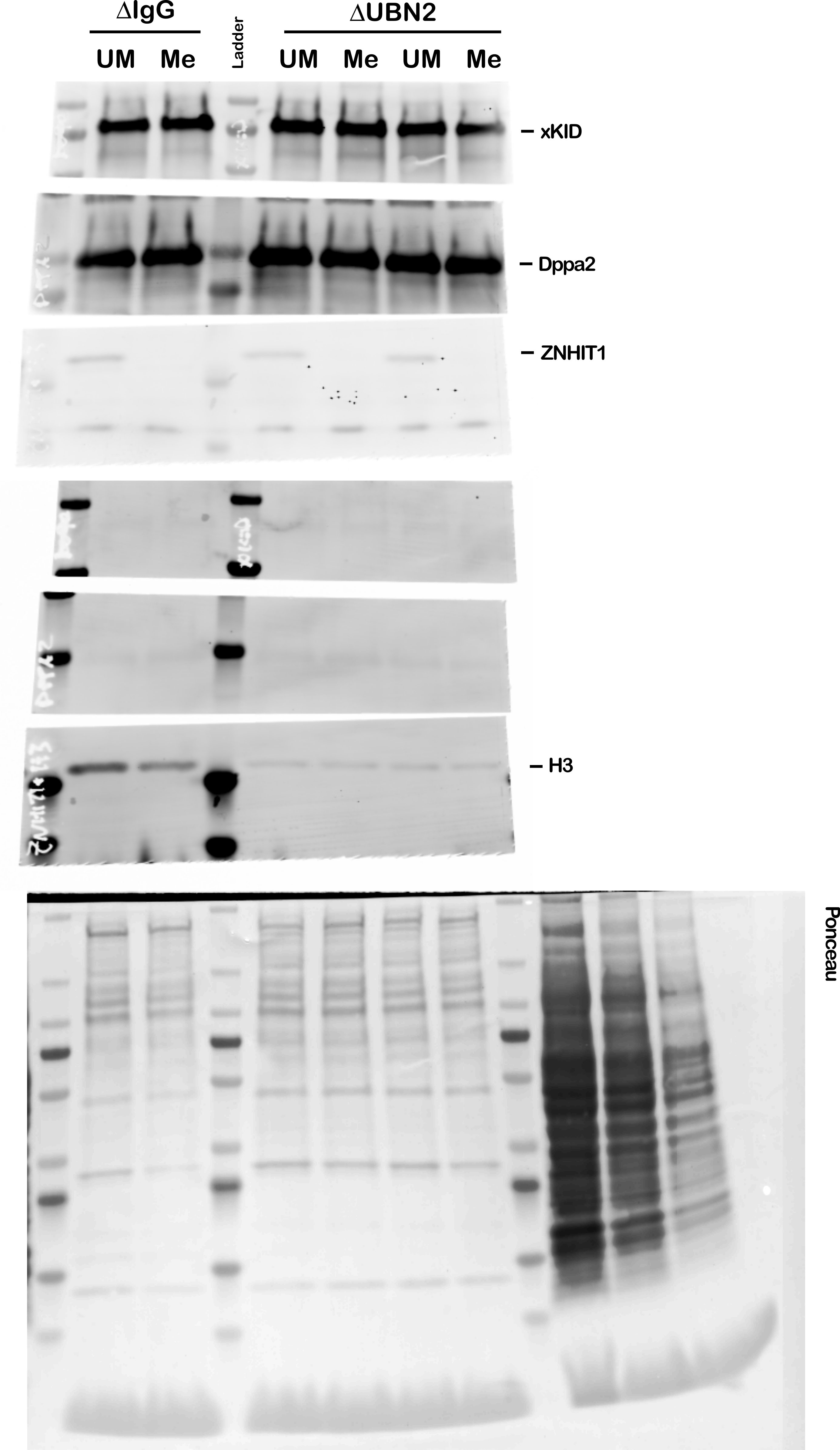

Supplement: Figure 5—figure supplement 4—source data 1. [file elife-109762-fig5-figsupp4-data1.zip › Figure 5 - figure supplement 4 - source data 1/Figure 5 - figure supplement 4 - source data 1 - 4C - LABELED.png]

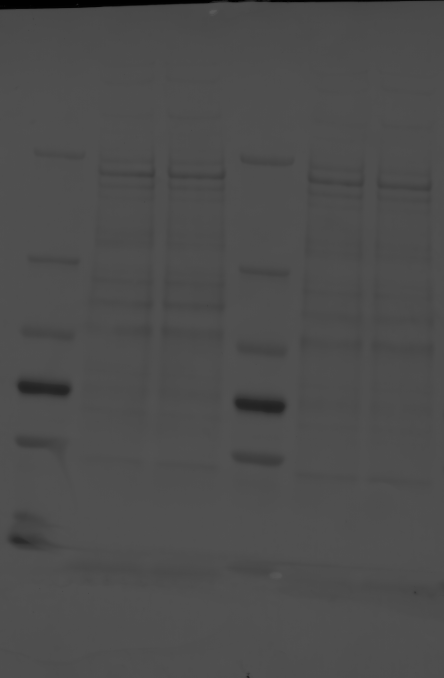

Supplement: Figure 5—figure supplement 4—source data 2. [file elife-109762-fig5-figsupp4-data2.zip › Figure 5 - figure supplement 4 - source data 2/Figure 5 - figure supplement 4 - source data 2 - 4B - ponceau - RAW.tif]

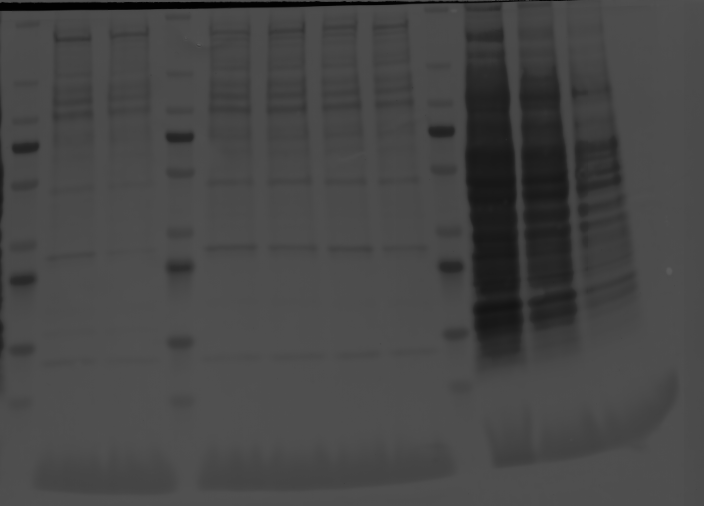

Supplement: Figure 5—figure supplement 4—source data 2. [file elife-109762-fig5-figsupp4-data2.zip › Figure 5 - figure supplement 4 - source data 2/Figure 5 - figure supplement 4 - source data 2 - 4C - ponceau - RAW.tif]

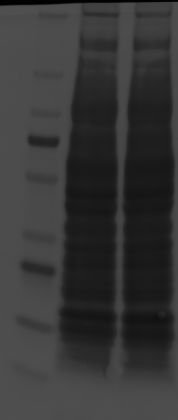

Supplement: Figure 5—figure supplement 4—source data 2. [file elife-109762-fig5-figsupp4-data2.zip › Figure 5 - figure supplement 4 - source data 2/Figure 5 - figure supplement 4 - source data 2 - 4A - ponceau - RAW.tif]
