# Supplementary material for: Impacts of DNA methylation on H2A.Z deposition and nucleosome stability
Source: eLife. 2026 Jul 7;15:RP109762. doi: 10.7554/eLife.109762 (PMC13341117; doi:10.7554/eLife.109762)
Supplement: Supplementary file 2. — XTC reads were downsampled to 30 million reads to match sperm samples, then all raw reads were trimmed and deduplicated using fastp v0.24.0 and aligned to the Xenopus laevis genome (Xenla 10.1) or lambda genome (to assess for bisulfite conversion efficiency) using Bismark v0.24.2. Methylated CpG percentages for H2A.Z samples were determined for the top 2.5% of H2A.Z peaks by AUC (obtained using SEACR v1.3) while methylated percentages for H3 associated CpGs were conducted on regions outside of determined H2A.Z peaks. All CpGs met a cutoff of at least five reads to be counted. Methylated lambda CpG statistics were calculated from all mapped CpGs with no filtering. [file elife-109762-supp2.docx]

| **Supplementary File 2. Alignment statistics of CnT-BS sequencing libraries.** XTC reads were downsampled to 30 million reads to match sperm samples, then all raw reads were trimmed and deduplicated using fastp v0.24.0 and aligned to the *Xenopus* laevis genome (Xenla 10.1) or lambda genome (to assess for bisulfite conversion efficiency) using Bismark v0.24.2. Methylated CpG percentages for H2A.Z samples were determined for the top 2.5 % of H2A.Z peaks by AUC (obtained using SEACR v1.3) while methylated percentages for H3 associated CpGs was conducted on regions outside of determined H2A.Z peaks. All CpGs met a cutoff of at least 5 reads to be counted. Methylated lambda CpG statistics were calculated from all mapped CpGs with no filtering. | | | | | | |
| --- | --- | --- | --- | --- | --- | --- |
|  |  | *Alignment to Xenopus laevis* | | | *Alignment to lambda* | |
|  |  | **Aligned %** | **Total Aligned Reads (No.)** | **Methylated CpG %** | **Aligned %** | **Methylated CpG %** |
| **Sperm Pronuclei** | |  |  |  |  |  |
| **H3** | *Rep 1* | 71.7 | 24141516 | **85.6** | 0.8 | **0.6** |
|  | *Rep 2* | 71.0 | 24185147 | **85** | 1.0 | **0.6** |
| **H2AZ** | *Rep 1* | 64.7 | 25362485 | **42.5** | 4.0 | **0.6** |
|  | *Rep 2* | 69.0 | 21963635 | **43.5** | 2.5 | **0.6** |
| **XTC-2** | |  |  |  |  |  |
| **H3** | *Rep 1* | 68.2 | 19539297 | **53.2** | 7.2 | **0.4** |
|  | *Rep 2* | 62.4 | 17758471 | **53.8** | 15.9 | **0.3** |
| **H2AZ** | *Rep 1* | 69.1 | 19409396 | **3.2** | 12.4 | **0.3** |
|  | *Rep 2* | 70.9 | 20109208 | **2.8** | 9.8 | **0.3** |
