# Supplementary material for: Impacts of DNA methylation on H2A.Z deposition and nucleosome stability
Source: eLife. 2026 Jul 7;15:RP109762. doi: 10.7554/eLife.109762 (PMC13341117; doi:10.7554/eLife.109762)
Supplement: Supplementary file 3. — A count matrix was generated from processed bam files over 1000 bp bins of the Xenopus laevis genome. Mitochondrial reads and bins containing fewer than 17 reads across less than 2samples were removed to filter out low signal areas. Replicates for each sample were averaged, and the resulting count matrix was used for genomic annotation analysis. [file elife-109762-supp3.docx]

| **Supplementary File 3. Fragment statistics of filtered genomic bins used for sequencing analysis.** A count matrix was generated from processed bam files over 1,000 bp bins of the *Xenopus laevis* genome. Mitochondrial reads and bins containing fewer than 17 reads across less than 2 samples were removed to filter out low signal areas. Replicates for each sample were averaged and resulting count matrix used for genomic annotation analysis. | | | |
| --- | --- | --- | --- |
|  |  | **Fragments in Bins (No.)** | **Fragments in Bins (%)** |
| **Sperm Pronuclei** | |  |  |
| **H3** | *Rep 1* | 9062125 | 34.04 |
|  | *Rep 2* | 9265292 | 34.68 |
| **H2AZ** | *Rep 1* | 11482371 | 43.04 |
|  | *Rep 2* | 9213698 | 39.58 |
| **XTC-2** | |  |  |
| **H3** | *Rep 1* | 7789646 | 36.43 |
|  | *Rep 2* | 8145900 | 41.79 |
| **H2AZ** | *Rep 1* | 14276029 | 66.62 |
|  | *Rep 2* | 15187573 | 69.13 |
| **Total Bins (No.)** | | 506009 | |
