# Supplementary material for: Impacts of DNA methylation on H2A.Z deposition and nucleosome stability
Source: eLife. 2026 Jul 7;15:RP109762. doi: 10.7554/eLife.109762 (PMC13341117; doi:10.7554/eLife.109762)
Supplement: Peer review history

# Editor report: eLife Assessment

**DOI:** 10.7554/eLife.109762.3.sa0

**Authors:** Daniel Zilberman

**Affiliations:** Institue of Science and Technology Austria

**Keywords:** Solid, Valuable

This study provides **valuable** mechanistic insight into the mutually exclusive distributions of the histone variant H2A.Z and DNA methylation by testing two hypotheses: (i) that DNA methylation suppresses H2A.Z deposition by ATP-dependent chromatin remodelling complexes, and (ii) that DNA methylation destabilizes H2A.Z nucleosomes, thereby preventing H2A.Z retention. Through a series of well-designed and carefully executed experiments, **solid** support is presented for the first hypothesis. The evidence supporting the second hypothesis is less complete, and the extent to which either mechanism is responsible for H2A.Z exclusion from methylated DNA remains not entirely clear. This work will be of broad interest to researchers in chromatin biology and epigenetics.

# Referee report: Reviewer #1 (Public review)

**DOI:** 10.7554/eLife.109762.3.sa1

Summary:

The authors considered the mechanism underlying previous observations that H2A.Z is preferentially excluded from methylated DNA regions. They considered two non-mutually exclusive mechanisms. First, they tested the hypothesis that nucleosomes containing both methylated DNA and H2A.Z might be intrinsically unstable due to their structural features. Second, they explored the possibility that DNA methylation might impede SRCAP-C from efficiently depositing H2A.Z onto these DNA methylated regions.

Their structural analyses revealed subtle differences between H2A.Z-containing nucleosomes assembled on methylated versus unmethylated DNA. To test the second hypothesis, the authors allowed H2A.Z assembly on sperm chromatin in Xenopus egg extracts and mapped both H2A.Z localization and DNA methylation in this transcriptionally inactive system. They compared these data with corresponding maps from a transcriptionally active Xenopus fibroblast cell line. This comparison confirmed the preferential deposition or enrichment of H2A.Z on unmethylated DNA regions, an effect that was much more pronounced in the fibroblast genome than in sperm chromatin. Furthermore, nucleosome assembly on methylated versus unmethylated DNA, along with SRCAP-C depletion from Xenopus egg extracts, provided a means to test whether SRCAP-C contributes to the preferential loading of H2A.Z onto unmethylated DNA.

Strengths:

The strength and originality of this work lie in its focused attempt to dissect the unexplained observation that H2A.Z is excluded from methylated genomic regions.

Weaknesses:

The study has two weaknesses. First, although the authors identify specific structural effects of DNA methylation on H2A.Z-containing nucleosomes, they do not provide evidence demonstrating that these structural differences lead to altered histone dynamics or nucleosome instability. Second, building on the elegant work of Berta and colleagues (cited in the manuscript), the authors implicate SRCAP-C in the selective deposition of H2A.Z at unmethylated regions. Yet the role of SRCAP-C appears only partial, and the study does not address how the structural or molecular consequences of DNA methylation prevent efficient H2A.Z deposition. Finally, additional plausible mechanisms beyond the two scenarios the authors considered are not investigated or discussed in the manuscript.

Comments on revisions:

The authors have addressed all previously raised concerns and propose a revised version of the manuscript. Notably, the abstract and discussion sections have been improved, and new experimental data have been incorporated. Collectively, these revisions enhance the rigor and clarity of the data interpretation and discussion.

Given these improvements, this reviewer believes that the manuscript could be published, particularly if this publication is accompanied by the critical points discussed in the rebuttal letter.

# Referee report: Reviewer #2 (Public review)

**DOI:** 10.7554/eLife.109762.3.sa2

This manuscript aims to elucidate the mechanistic basis for the long-standing observation that DNA methylation and the histone variant H2A.Z occupy mutually exclusive genomic regions. The authors test two hypotheses: (i) that DNA methylation intrinsically destabilizes H2A.Z nucleosomes, thereby preventing H2A.Z retention, and (ii) that DNA methylation suppresses H2A.Z deposition by ATP-dependent chromatin-remodelling complexes. The revised manuscript addresses a number of previous concerns, and the manuscript has therefore improved accordingly. However, several limitations remain.

Comments on revisions:

The authors have addressed a number of my previous concerns, and the manuscript has improved accordingly. However, several limitations remain that, in my view, constrain the strength of the conclusions. In particular, the absence of a direct comparison with a canonical nucleosome assembled on the same DNA template. This control is essential to determine whether the observed effects are specific to H2A.Z or reflect more general properties of methylated DNA-nucleosome interactions. Notably, even within the authors' own data, there is a trend suggesting that methylated canonical H2A nucleosomes may also exhibit increased accessibility. Although this does not reach statistical significance, the authors themselves argue that subtle differences can be biologically meaningful; it is therefore plausible that extended digestion conditions (e.g., longer HinfI exposure) could reveal a significant effect. Unless a direct structural comparison with a canonical nucleosome is performed, the possibility that the reported phenomenon is not specific to H2A.Z remains. This is compounded by the reliance on a single restriction enzyme-based assay, which represents a limited experimental approach. Such an approach is insufficient to unequivocally support the central claim that DNA methylation increases accessibility of H2A.Z-containing nucleosomes. Additional orthogonal assays would be required to substantiate this conclusion. With respect to the cryo-EM analysis of methylated and unmethylated 601L H2A.Z nucleosomes, and in general, the authors still do not adequately consider the positional context of CpG methylation. Extensive literature demonstrates that the effects of DNA methylation on canonical nucleosome structure and stability are highly position-dependent. Without accounting for the location of methylated CpGs relative to key DNA-histone contact sites, the structural data remain difficult to interpret mechanistically. Overall, while the manuscript has improved, it remains a relatively limited study that draws broad mechanistic conclusions from a minimal experimental data.

# Referee report: Reviewer #3 (Public review)

**DOI:** 10.7554/eLife.109762.3.sa3

Summary:

Histone variant H2A.Z is evolutionarily conserved among various species. The selective incorporation and removal of histone variants on the genome play crucial roles in regulating nuclear events, including transcription. Shih et al. aimed to address antagonistic mechanisms between histone variant H2A.Z deposition and DNA methylation. To this end, the authors reconstituted H2A.Z nucleosomes in vitro using methylated or unmethylated human satellite II DNA sequence and examined how DNA methylation affects H2A.Z nucleosome structure and dynamics. The cryo-EM analysis revealed that DNA methylation induces a more open conformation in H2A.Z nucleosomes. Consistent with this, their biochemical assays showed that DNA methylation subtly increases restriction enzyme accessibility in H2A.Z nucleosomes compared with canonical H2A nucleosomes. The authors identified genome-wide profiles of H2A.Z and DNA methylation using genomic assays and found their unique distribution between Xenopus sperm pronuclei and fibroblast cells. Using Xenopus egg extract systems, the authors showed SRCAP complex, the chromatin remodelers for H2A.Z deposition, preferentially bind to unmethylated DNA to deposit H2A.Z.

Strengths:

The experiments are rigorously performed, and interpretations are clear. The study presents a high-resolution cryo-EM structure of human H2A.Z nucleosome with methylated DNA. Although the effect of DNA methylation on the physical stability of the H2A.Z nucleosome is subtle, this would be important finding that warrants further functional investigation. The discovery that the SRCAP complex senses DNA methylation is novel and provides important mechanistic insight into the antagonism between H2A.Z and DNA methylation.

Weaknesses:

The authors have satisfactorily addressed my concerns.

# Author comment: Author response

**DOI:** 10.7554/eLife.109762.3.sa4

**Authors:** Rochelle M Shih, Yasuhiro Arimura, Hide A Konishi, Hironori Funabiki

**Affiliations:** Rockefeller University New York United States; Fred Hutch Cancer Center Seattle United States; Rockefeller University New York United States; Rockefeller University New York United States

The following is the authors’ response to the current reviews.

Reviewer #1.

We appreciate the constructive comments, which greatly improved this manuscript.

Reviewer #2.

We appreciate Reviewer #2's thorough analysis of our manuscript. However, we are concerned that the reviewer criticized a conclusion different from the one we claim in the manuscript. Although Reviewer #2's public comment stated, "Such an approach is insufficient to unequivocally support the central claim that DNA methylation increases accessibility of H2A.Z-containing nucleosomes", we did not draw such a bold conclusion. In the Abstract, we cautiously described that the impact of DNA methylation we observed was subtle and based on satellite II-derived DNA sequences. We made a nuanced proposal regarding this observation, stating, "Altogether, we propose that SRCAP drives the biased association of H2A.Z to unmethylated DNA, while additional mechanisms, potentially taking advantage of the subtle DNA methylation-induced physical effects, further assist the exclusion of H2A.Z from methylated DNA". We believe our analysis will contribute valuable insights into the mechanistic basis behind the antagonism between DNA methylation and H2A.Z.

Reviewer #3.

We appreciate the constructive comments, which greatly improved this manuscript.

The following is the authors’ response to the original reviews.

**eLife Assessment**

This study provides valuable mechanistic insight into the mutually exclusive distributions of the histone variant H2A.Z and DNA methylation by testing two hypotheses: (i) that DNA methylation destabilizes H2A.Z nucleosomes, thereby preventing H2A.Z retention, and (ii) that DNA methylation suppresses H2A.Z deposition by ATP-dependent chromatin remodeling complexes. Through a series of well-designed and carefully executed experiments, findings are presented in support of both hypotheses. However, the evidence in support of either hypothesis is incomplete, so that the proposed mechanisms underlying the enrichment of H2A.Z on unmethylated DNA remain somewhat speculative.

We would like to thank the editor and reviewers for their critical assessments of our manuscript. While we do acknowledge the limitations of our work, we believe that our results provide important mechanistic insights into the long-standing question of how H2A.Z is preferentially enriched in hypomethylated genomic DNA regions. First, our structural and biochemical data suggest that DNA methylation increases the openness and physical accessibility of H2A.Z, albeit the effect is relatively subtle and is sequence-dependent. Second, using *Xenopus* egg extracts and synthetic DNA templates, we provide the first clear and direct evidence that DNA methylation-sensitive H2A.Z deposition is due to the H2A.Z chaperone SRCAP-C, corroborated by our discovery that SRCAP-C binding to DNA is suppressed by DNA methylation. Although the molecular details by which DNA methylation inhibits binding of SRCAP-C is an important area of future study, in our current manuscript, we do provide evidence that directly links the presence of SRCAP-C to the establishment of the DNA methylation/H2A.Z antagonism in a physiological system. Thanks to criticisms by the reviewers, we realized that we did not clearly state in our Abstract that the impact of DNA methylation on intrinsic H2A.Z nucleosome stability is relatively subtle, although we did explain these observations and limitations in the main text. In our revised manuscript, we are willing to edit the text to better clarify the criticisms raised by the reviewers.

**Public Reviews:**

**Reviewer #1 (Public review):**

Summary:

The authors considered the mechanism underlying previous observations that H2A.Z is preferentially excluded from methylated DNA regions. They considered two non-mutually exclusive mechanisms. First, they tested the hypothesis that nucleosomes containing both methylated DNA and H2A.Z might be intrinsically unstable due to their structural features. Second, they explored the possibility that DNA methylation might impede SRCAP-C from efficiently depositing H2A.Z onto these DNA methylated regions.

Their structural analyses revealed subtle differences between H2A.Z-containing nucleosomes assembled on methylated versus unmethylated DNA. To test the second hypothesis, the authors allowed H2A.Z assembly on sperm chromatin in Xenopus egg extracts and mapped both H2A.Z localization and DNA methylation in this transcriptionally inactive system. They compared these data with corresponding maps from a transcriptionally active Xenopus fibroblast cell line. This comparison confirmed the preferential deposition or enrichment of H2A.Z on unmethylated DNA regions, an effect that was much more pronounced in the fibroblast genome than in sperm chromatin. Furthermore, nucleosome assembly on methylated versus unmethylated DNA, along with SRCAP-C depletion from Xenopus egg extracts, provided a means to test whether SRCAP-C contributes to the preferential loading of H2A.Z onto unmethylated DNA.

Strengths:

The strength and originality of this work lie in its focused attempt to dissect the unexplained observation that H2A.Z is excluded from methylated genomic regions.

Weaknesses:

The study has two weaknesses. First, although the authors identify specific structural effects of DNA methylation on H2A.Z-containing nucleosomes, they do not provide evidence demonstrating that these structural differences lead to altered histone dynamics or nucleosome instability. Second, building on the elegant work of Berta and colleagues (cited in the manuscript), the authors implicate SRCAP-C in the selective deposition of H2A.Z at unmethylated regions. Yet the role of SRCAP-C appears only partial, and the study does not address how the structural or molecular consequences of DNA methylation prevent efficient H2A.Z deposition. Finally, additional plausible mechanisms beyond the two scenarios the authors considered are not investigated or discussed in the manuscript.

Although we acknowledge the limitations of our study and are willing to expand our discussion to more thoroughly discuss these points, we believe our manuscript provides several important mechanistic insights which this reviewer may not have fully appreciated.

Our first conclusion that H2A.Z nucleosomes on methylated DNA are more open and accessible compared to their unmethylated counterparts is supported by both our cryo-EM study and the restriction enzyme accessibility assay. Although the physical effect of DNA methylation is relatively subtle and is likely sequence dependent, as we clearly noted within the manuscript, the difference does exist and is valuable information for the chromatin field at large to consider.

The second major conclusion of our manuscript is that SRCAP-C exhibits preferential binding to unmethylated DNA over methylated DNA, and that SRCAP-C represents the major mechanism that can explain the biased deposition of H2A.Z to unmethylated DNA in *Xenopus* egg extracts. Furthermore, our experiments using *Xenopus* egg extract clearly demonstrated that H2A.Z is deposited by both DNAmethylation sensitive and insensitive mechanisms. Depletion of SRCAP-C almost completely eliminated the levels of DNA-methylation-sensitive H2A.Z deposition and reduced the total level of H2A.Z on chromatin to less than half of that seen in non-depleted extract. This result demonstrated that DNA methylation-sensitive H2A.Z loading is primarily regulated by SRCAP-C, at least in our experimental context where transcription, replication, and other epigenetic modifications are not involved. It is likely that additional mechanisms do further contribute, implicated by our sequencing experiments, particularly at regions with active transcription, and we have noted these possibilities and the rationale for their existence in the Discussion.

Our study also suggests that a SRCAP-independent, DNA methylation-insensitive mechanism of H2A.Z loading exists, which we suspect to be mediated by Tip60-C. In line with this possibility, our data suggest that Tip60-C binds DNA in a DNA methylation-insensitive manner in *Xenopus* egg extract. Since antibodies to deplete Tip60-C from *Xenopus* egg extract are currently unavailable, we were unable to directly test that hypothesis and decided not to include Tip60-C into our final model as we lacked experimental evidence for its role. However, whether or not Tip60-C is the complex responsible for the DNA methylation-insensitive pathway does not influence our final conclusion that SRCAP-C plays a major role in DNA methylation-sensitive H2A.Z loading. We are planning to edit our manuscript to more comprehensively discuss these points.

Please note that while Berta et al reported that DNA methylation increases at H2A.Z loci in tumors defective in SRCAP-C, they selected those regions based off where H2A.Z is typically enriched within normal tissues (Berta et al., 2021). They did not show data indicating whether H2A.Z is still retained specifically at those analyzed loci upon mutation of SRCAP-C subunits. Thus, although we greatly admire their work and are pleased that many of our findings align with theirs, their paper did not directly address whether SRCAP-C itself differentiates between DNA methylation status nor the impact that has on H2A.Z and DNA methylation colocalization. In contrast, our *Xenopus* egg extract system, where de novo methylation is undetectable (Nishiyama et al., 2013; Wassing et al., 2024) offers a unique opportunity to examine the direct impact of DNA methylation on H2A.Z deposition using controlled synthetic DNA substrates. Corroborated with our demonstration that DNA binding of SRCAP-C is suppressed by DNA methylation, we believe that our manuscript provides a specific mechanism that can explain the preferential deposition of H2A.Z at hypomethylated genomic regions.

**Reviewer #2 (Public review):**

This manuscript aims to elucidate the mechanistic basis for the long-standing observation that DNA methylation and the histone variant H2A.Z occupy mutually exclusive genomic regions. The authors test two hypotheses: (i) that DNA methylation intrinsically destabilizes H2A.Z nucleosomes, thereby preventing H2A.Z retention, and (ii) that DNA methylation suppresses H2A.Z deposition by ATPdependent chromatin-remodelling complexes. However, neither hypothesis is rigorously addressed. There are experimental caveats, issues with data interpretation, and conclusions that are not supported by the data. Substantial revision and additional experiments, including controls, would be required before mechanistic conclusions can be drawn. Major concerns are as follows:

We appreciate the critical assessment of our manuscript by this reviewer. Although we acknowledge the limitations of our study and will revise the manuscript to better describe them, we would like to respectfully argue against the statement that our "conclusions […] are not supported by the data".

(1) The cryo-EM structure of methylated H2A.Z nucleosomes is insufficiently resolved to address the central mechanistic question: where the methylated CpGs are located relative to DNA-histone contact points and how these modifications influence H2A.Z nucleosome structure. The structure provides no mechanistic insights into methylation-induced destabilization.

The fact that the DNA resolution in the methylated structure was not high enough to resolve the positions of methylated CpGs despite a high overall resolution of 2.78 Å implies that (1) the Sat2R-P DNA was not as stably registered as the 601L sequence, requiring us to create two alternative Sat2R-P atomic models to account for the variable positioning in our samples, and (2) that the presence of DNA methylation increases that positional variability. We understand that one may prefer to see highly resolved density around each methylation mark, but we do believe that our inability to accomplish that is actually a feature rather than a weakness and has important biological implications. The decrease in local DNA resolution on the methylated Sat2R-P structure compared to its unmethylated counterpart is meaningful and suggests to us that DNA methylation weakens overall DNA wrapping and positioning on the nucleosome, supported by the increased flexibility seen at the linker DNA ends as well as an increase in the population of highly shifted nucleosomes amongst the methylated particles. Additionally, one major view in the DNA methylation/nucleosome stability field is that the presence of DNA methylation can make DNA stiffer and harder to bend, causing opening and destabilization of nucleosomes (Ngo et al., 2016). The increased opening of linker DNA ends and accessibility of methylated H2A.Z nucleosomes in our hands also aligns with such an idea, again suggesting decreased histone-DNA contact stability on methylated DNA substrates. We plan to revise the writing in our manuscript to better reflect these ideas.

The experimental system also lacks physiological relevance. The template DNA sequence is artificial, despite the existence of well-characterised native genomic sequences for which DNA methylation is known to inhibit H2A.Z incorporation. Alternatively, there are a number of studies examining the effect of DNA methylation on nucleosome structure, stability, DNA unwrapping, and positioning. Choosing one of these DNA sequences would have at least allowed a direct comparison with a canonical nucleosome. Indeed, a major omission is the absence of a cryo-EM structure of a canonical nucleosome assembled on the same DNA template - this is essential to assess whether the observed effects are H2A.Z-specific.

The reviewer raises a fair question about whether canonical H2A would experience the same DNA methylation-dependent structural effects. We had considered solving the H2A structures, however, ultimately decided against it for a few reasons. First, there already exists crystal structures of canonical H2A nucleosomes using a DNA sequence highly similar to our Sat2R-P with and without the presence of DNA methylation (PDB: 5CPI and 5CPJ). The authors of this study did not see any physical differences present in their structures (Osakabe et al., 2015). Additionally, we had included canonical H2A conditions within our restriction enzyme accessibility assay and did not see a significant impact of DNA methylation on those samples (Fig 3). Because of the previous report and our own negative data, we expected that only limited additional insights would be obtained from the canonical H2A structures and decided not to pursue that analysis.

One of the primary reasons we chose the Sat2R-P sequence was, as noted above, that there already was a published study examining how DNA methylation affects nucleosome structure using a variant of this sequence which we could compare to our results, as the reviewer has suggested. We did have to modify the sequence, namely by making it palindromic, in order to increase the final achievable resolution. We viewed the Sat2R-P sequence as an attractive candidate because it is physiologically relevant; the initial sequence was taken directly from human satellite II. Several modifications were made for technical reasons, including making the sequence palindromic as described above and also ensuring that each CpG is recognizable by a methylation-sensitive restriction enzyme so that we could be certain about the degree of methylation on our substrates. These practical concerns outweighed the necessity of maintaining a strict physiological sequence to us. However, we still believe the final Sat2R-P more closely mimics physiological sequences than Widom 601. Additionally, human satellite II is a highly abundant sequence in the human genome that is known to undergo large methylation changes on the onset of many disorders, like cancer, as well as during aging. Thus, there are interesting biological questions surrounding how the methylation state of this particular sequence affects chromatin structure.

Furthermore, it has been reported that satellite II is devoid of H2A.Z (Capurso et al., 2012). Beyond those reasons, the satellite II sequence is generally interesting to our lab because we have been studying genes involved in ICF syndrome, where hypomethylation of satellite II sequences forms one of the hallmarks of this disorder (Funabiki et al., 2023; Jenness et al., 2018; Wassing et al., 2024). We understand that sequence context plays a large role in nucleosome wrapping and stability. This is why we strived to test multiple sequences in each of our assays. We do agree that it would be interesting to use DNA sequences where H2A.Z binding has already been described to be affected in a DNA methylation-dependent manner, forming an exciting future study to pursue.

Furthermore, the DNA template is methylated at numerous random CpG sites. The authors' argument that only the global methylation level is relevant is inconsistent with the literature, which clearly demonstrates that methylation effects on canonical nucleosomes are position-dependent. Not all CpG sites contribute equally to nucleosome stability or unwrapping, and this critical factor is not considered.

We did not argue that only the global methylation level is relevant. We also would appreciate it if the reviewer could provide specific references that "clearly demonstrates that methylation effects on canonical nucleosomes are position-dependent". We are aware of a series of studies conducted by Chongli Yuan's group, including one testing the effect of placing methylated CpGs at different positions along the Widom 601 sequence. In that study (Jimenez-Useche et al., 2013), they did find that positioning of mCpGs has differential impacts on the salt resistance of the nucleosomes, with 5 tandem mCpG copies at the dyad causing the most dramatic nucleosome opening whereas having mCpGs only at the DNA major grooves, but not elsewhere, increased nucleosome stability. However, they did also find that methylation of the original Widom 601 sequence also caused destabilization, albeit to a lesser degree, and another study by the same group (Jimenez-Useche et al., 2014) also found that CpG methylation decreased nucleosome-forming ability for all tested variants of the Widom 601 sequence, regardless of CpG density or positioning.

Other studies monitored how distribution of methylated CpGs correlates with nucleosome positioning (Collings et al., 2013; Davey et al., 1997; Davey et al., 2004). However, these studies assessed the sequence-dependent effects specifically on nucleosome assembly during in vitro salt dialysis, which is a different physical process than the one our manuscript focuses on, especially when considering the fact that H2A.Z is deposited onto preassembled H2A-nucleosome. Our cryo-EM analysis examines the structural changes induced by DNA methylation on already formed nucleosomes rather than the process of formation. Thus, probing accessibility changes using a restriction enzyme was the more appropriate biochemical assay to verify our structures.

We do very much agree that DNA context can influence nucleosome stability under different conditions. A study of molecular dynamics simulations concluded that the "combination of overall DNA geometrical and shape properties upon methylation" makes nucleosomes resistant to unwrapping (Li et al., 2022), while another modeling study suggests that DNA methylation impacts nucleosome stability in a manner dependent on DNA sequence, where "[s]trong binding is weakened and weak binding is strengthened" (Minary and Levitt, 2014). While G/C-dinucleotides are preferentially placed at major groove-inward positions in the nucleosomes in vivo (Chodavarapu et al., 2010; Segal et al., 2006) and G/C-rich segments are excluded from major groove-outward positions in Widom 601-like nucleosomes (Chua et al., 2012), methylated CpG dinucleotides are preferably, if not exclusively, located at major groove-outward positions in vivo. Mechanisms behind this biased mCpG positioning on the nucleosome remain speculative, likely caused by a combination of multiple factors, but the fact that we did not observe clear structural impacts using the Widom 601L sequence, where mCpGs are located at the major groove-outward and -inward positions ((Chua et al., 2012) and our structure), deserves a space for discussion. On the other hand, positioning of mCpG on satellite II-derived sequences that we used in this study was based on a physiological sequence, and thus it may not be appropriate to say that those CpGs are placed at multiple "random" positions. Although we decided not to discuss the position of 5mC on our Sat2R nucleosome structure due to ambiguous base assignments, neither of our two atomic models is consistent with an idea that DNA methylation repositions the CpG to the outward major grooves. As the potential contribution of how DNA methylation affects the nucleosome structure via modulating DNA stiffness has been extensively studied (Choy et al., 2010; Li et al., 2022; Ngo et al., 2016; Perez et al., 2012), we believe that it is appropriate to consider overall DNA properties along the whole DNA sequence, though we are willing to discuss potential positional effects in the revised manuscript.

Perhaps one of the most important points that we did not emphasize enough in our original manuscript was that in contrast to the subtle intrinsic effect of DNA methylation that was DNA sequence dependent, we observed SRCAP-dependent preferential H2A.Z deposition to unmethylated DNA over methylated DNA in both 601 and satellite II DNAs. In the revised manuscript, we will make the value of comparative studies on 601 and satellite II in two distinct mechanisms.

Finally, and most importantly, the reported increase in accessibility of the methylated H2A.Z nucleosome is negligible compared with the much larger intrinsic DNA accessibility of the unmethylated H2A.Z nucleosome. These data do not support the authors' hypothesis and contradict the manuscript's conclusions. Claims that methylated H2A.Z nucleosomes are "more open and accessible" must therefore be removed, and the title is misleading, given that no meaningful impact of DNA methylation on H2A.Z nucleosome stability is demonstrated.

We respectfully disagree with this reviewer's criticism. We investigated the potential impact of DNA methylation on nucleosome stability to the best of our abilities through complementary assays and reported our observations. The effect of DNA methylation is smaller than the difference between H2A.Z and H2A, but we were able to see an effect. It is also not uncommon for small differences to have functional impacts in biological systems. We agree that further testing is required to determine whether this subtle effect is functionally important, and it remains the subject of future research due to the many technical challenges associated with addressing said question. We would like to note that 18 years have passed since Daniel Zilberman first reported the antagonistic relationship between H2AZ and DNA methylation (Zilberman et al., 2008) but very few studies have since directly tested specific mechanistic hypotheses. We believe that our study lays the groundwork for exciting future investigation that better elucidates the pathways that contribute to this antagonism and will have meaningful impacts on the field in general. However, thanks to the reviewer's criticism, we realized that we did not clearly state in the Abstract the relatively subtle effect of DNA methylation on the intrinsic H2A.Z nucleosome stability. Therefore, we will accordingly revise the Abstract to make this point clearer.

(2) The cryo-EM structures of methylated and unmethylated 601L H2A.Z nucleosomes show no detectable differences. As presented, this negative result adds little value. If anything, it reinforces the point that the positional context of CpG methylation is critical, which the manuscript does not consider.

We believe the inclusion and factual reporting of negative data is important for the scientific community as one of the major issues currently in biology research is biased omission of negative data. We considered eLife as a venue to publish this work for this reason. We understand that the reviewer believes our 601L structures may detract from the overall message of our manuscript. We believe this data rather emphasizes the importance of DNA sequence context, something that the reviewer also rightfully notes. It is standard practice in the nucleosome field to use the Widom 601 sequence, along with its variants. Our experience has shown that use of an artificially strong positioning sequence may mask weaker physical effects that could play a physiological role. Thus, we were careful to validate all further assays with multiple DNA sequences and believed it important to report these sequence-dependent effects on nucleosome structure.

(3) Very little H3 signal coincides with H2A.Z at TSSs in sperm pronuclei, yet this is neither explained nor discussed (Supplementary Figure 10D). The authors need to clarify this.

Our H3 signal, which represents the global nucleosome population, is more broadly distributed across the genome than H2A.Z, which is known to localize at specific genomic sites. Since both histone types were sequenced to similar read depths, H3 peaks are generally shallower than H2A.Z and peak heights cannot be directly compared (i.e. they should be represented in separate appropriate data ranges).

(4) In my view, the most conceptually important finding is that H2A.Z-associated reads in sperm pronuclei show ~43% CpG methylation. This directly contradicts the model of strict mutual exclusivity and suggests that the antagonism is context-dependent. Similarly, the finding that the depletion of SRCAP reduces H2A.Z deposition only on unmethylated templates is also very intriguing. Collectively, these result warrants further investigation (see below).

(5) Given that H2A.Z is located at diverse genomic elements (e.g., enhancers, repressed gene bodies, promoters), the manuscript requires a more rigorous genomic annotation comparing H2A.Z occupancy in sperm pronuclei versus XTC-2 cells. The authors should stratify H2A.Z-DNA methylation relationships across promoters, 5′UTRs, exons, gene bodies, enhancers, etc., as described in Supplementary Figure 10A.

We agree that the substantial presence of co-localized H2A.Z and DNA methylation specifically in the sperm pronuclei samples and the changes in pattern between nuclear types are highly interesting and require further investigation. However, we faced technical challenges in our sequencing experiments that made us refrain from conducting a more detailed analysis for fear of over-interpreting potential artifacts. These challenges mainly stemmed from the difficulties in collecting enough material from *Xenopus* egg extracts and Tn5’s innate bias towards accessible regions of the genome. Because of this, open regions of the genome tend to be overrepresented in our data (as noted in our Discussion), making it challenging to rigorously compare methylation profiles and H2A.Z/H3 associated genomic elements.

While the degree of separation seems to be dependent on nuclei type, we still believe the antagonism exists in both the sperm pronuclei and XTC-2 samples when comparing H2A.Z methylation profiles to the corresponding H3 condition. Our study also demonstrates that H2A.Z is preferentially deposited to hypomethylated DNA in a manner dependent of SRCAP-C (the loss of SRCAP only reduces H2A.Z on unmethylated substrates) but an additional methylation-insensitive H2A.Z deposition mechanism also exists. We realized that this interesting point was not clearly highlighted in Abstract, so we will revise it accordingly.

(6) Although H2A.Z accumulates less efficiently on exogenous methylated substrates in egg extract, substantial deposition still occurs (~50%). This observation directly challenges the strong antagonistic model described in the manuscript, yet the authors do not acknowledge or discuss it. Moreover, differences between unmethylated and methylated 601 DNA raise further questions about the biological relevance of the cryo-EM 601 structures.

As depicted in Figure 6 and described in the Discussion, we clearly indicated that both methylation-sensitive and methylation-insensitive pathways exist to deposit H2A.Z within the genome. We also directly stated in our Discussion that a substantial proportion of H2A.Z colocalizes with DNA methylation both in our study as well as in previous reports, which is of major interest for future study. Additionally, we further discussed how the absence of transcription in *Xenopus* eggs is a likely reason for the more limited effect of DNA methylation restricting H2A.Z deposition in our egg extract system.

As noted in our response to (2), the lack of a clear impact on our 601L structures implies that this is due to the extraordinarily strong artificial nucleosome positioning capacity of the 601 sequence and its variants. Since 601 is heavily used in chromatin biology, including within DNA methylation research, such negative data are still useful to include and publish.

(7) The SRCAP depletion is insufficiently validated i.e., the antibody-mediated depletion of SRCAP lacks quantitative verification. A minimum of three biological replicates with quantification is required to substantiate the claims.

We are willing to address this concern. However, please note that our data showed that methylation-dependent H2A.Z deposition is almost completely erased upon SRCAP depletion, indicating functionally effective depletion. The specificity of the custom antibody against Xenopus SRCAP was verified by mass spectrometry. Additionally, we have obtained the same effect using another commercially available SRCAP antibody, though we did not include this preliminary result in our original manuscript. Due to its relatively low abundance and high molecular weight, SRCAP western blot signals are weak, making it challenging to quantify the degree of depletion. We also believe that the value of quantification in this context, with the points noted above, is rather limited. In the past, our lab has published papers on depleting the H3T3 kinase Haspin from *Xenopus* egg extracts (Ghenoiu et al., 2013; Kelly et al., 2010) but were never able to detect Haspin via western blot. This protein was only detected by mass spectrometry specifically on nucleosome array beads with H3K9me3 (Jenness et al., 2018). However, depletion of Haspin was readily monitored by erasure of H3T3ph, the enzymatic product of Haspin. In these experiments, it was impossible, and not critical, to quantitatively monitor the depletion of Haspin protein in order to investigate its molecular functions. Similarly, in this current study, the important fact is that depletion of SRCAP suppressed methylation-sensitive H2A.Z deposition and quantifying the degree of SRCAP depletion would not have a major impact on this conclusion.

(8) It appears that the role of p400-Tip60 has been completely overlooked. This complex is the second major H2A.Z deposition complex. Because p400 exhibits DNA methylation-insensitive binding (Supplementary Figure 14), it may account for the deposition of H2A.Z onto methylated DNA. This possibility is highly significant and must be addressed by repeating the key experiments in Figure 5 following p400-Tip60 depletion.

We are aware that the Tip60 complex is a very likely candidate for mediating DNA methylation insensitive H2A.Z deposition, which is why we tested whether DNA binding of p400 is methylation sensitive. Therefore, the reviewer's statement that we "completely overlooked" Tip60-C’s role does not fairly report on our efforts. We wished to test the potential contribution of Tip60-C, but, unfortunately, the antibodies we currently have available to us were not successful in depleting the complex from egg extract. Since we had no direct experimental evidence indicating the role Tip60-C plays, we decided to take a conservative approach to our model and leave the methylation-insensitive pathway as mediated by something still unidentified. While further investigating Tip60-C’s contribution to this pathway is of definite value, we do not believe that it impacts our major conclusion that SRCAP-C is the main mediator responsible for H2A.Z deposition on unmethylated DNA and thus remains a subject for future study.

(9) The manuscript repeatedly states that H2A.Z nucleosomes are intrinsically unstable; however, this is an oversimplification. Although some DNA unwrapping is observed, multiple studies show that H3/H4 tetramer-H2A.Z/H2B interactions are more stable (important recent studies include the following: DOI: 10.1038/s41594-021-00589-3; 10.1038/s41467-021-22688-x; and reviewed in 10.1038/s41576-02400759-1).

We understand that the H2A.Z stability field is highly controversial. We have introduced the many conflicting reports that have been published in the field but can further expand on the controversies if desired. We also understand that the term “nucleosome stability” is broad and encompasses many physical aspects. As noted in a prior response, we will better specify our use of the term within the manuscript. In our assays, we are most focused on the DNA wrapping stability of the nucleosome and have consistently seen in our hands that H2A.Z nucleosomes are much more open and accessible compared to canonical H2A on satellite II-derived sequences, regardless of methylation status. However, we do understand that many groups have observed the opposite findings while others have obtained results similar to us. We reported on our findings of the general H2A.Z stability with the hopes to help clarify some of the field’s controversies.

In summary, the current manuscript does not present a convincing mechanistic explanation for the antagonism between DNA methylation and H2A.Z. The observation that H2A.Z can substantially coexist with DNA methylation in sperm pronuclei, perhaps, should be the conceptual focus.

We appreciate this reviewer’s advice. However, please note that the first author who led this project has already successfully defended their PhD thesis primarily based on this project, making it impractical and unrealistic to completely change the focus of this manuscript to include an entirely new avenue of research. We believe that our data provide important insights into the mechanisms by which H2A.Z is excluded from methylated DNA, particularly via the DNA methylation-sensitive binding of SRCAP-C, which has never been described before. We agree that many questions are still left unanswered, including the exact molecular mechanism behind how DNA methylation prevents SRCAP-C binding. We have preliminary data that suggest none of the known DNA-binding modules of SRCAP-C, including ZNHIT1, by themselves can explain this sensitivity. This implies that domain dissection in the context of the holo-SRCAP complex is required to fully address this question. We believe this represents a very exciting future avenue of study; however, it does not negate our finding that SRCAP-C itself is important for maintaining the DNA methylation/H2A.Z antagonism. Therefore, we respectfully disagree with this reviewer's summary statement, which misleadingly undermines the impact of our work.

**Reviewer #3 (Public review):**

Summary:

Histone variant H2A.Z is evolutionarily conserved among various species. The selective incorporation and removal of histone variants on the genome play crucial roles in regulating nuclear events, including transcription. Shih et al. aimed to address antagonistic mechanisms between histone variant H2A.Z deposition and DNA methylation. To this end, the authors reconstituted H2A.Z nucleosomes in vitro using methylated or unmethylated human satellite II DNA sequence and examined how DNA methylation affects H2A.Z nucleosome structure and dynamics. The cryo-EM analysis revealed that DNA methylation induces a more open conformation in H2A.Z nucleosomes. Consistent with this, their biochemical assays showed that DNA methylation subtly increases restriction enzyme accessibility in H2A.Z nucleosomes compared with canonical H2A nucleosomes. The authors identified genome-wide profiles of H2A.Z and DNA methylation using genomic assays and found their unique distribution between Xenopus sperm pronuclei and fibroblast cells. Using Xenopus egg extract systems, the authors showed SRCAP complex, the chromatin remodelers for H2A.Z deposition, preferentially deposit H2A.Z on unmethylated DNA.

Strengths:

The study is solid, and most conclusions are well-supported. The experiments are rigorously performed, and interpretations are clear. The study presents a high-resolution cryo-EM structure of human H2A.Z nucleosome with methylated DNA. The discovery that the SRCAP complex senses DNA methylation is novel and provides important mechanistic insight into the antagonism between H2A.Z and DNA methylation.

We are grateful that this reviewer recognizes the importance of our study.

Weaknesses:

The study is already strong, and most conclusions are well supported. However, it can be further strengthened in several ways.

(1) It is difficult to interpret how DNA methylation alters the orientation of the H4 tail and leads to the additional density on the acidic patch. The data do not convincingly support whether DNA methylation enhances interactions with H2A.Z mono-nucleosomes, nor whether this effect is specific to methylated H2A.Z nucleosomes.

The altered H4 tail orientation and extra density seen on the acidic patch were incidental findings that we thought could be interesting for the field to be aware of but decided not to follow up on as there were other structural differences that were more directly related to our central question. We do believe that the above two differences are linked to each other because we used a highly purified and homogenous sample for cryo-EM analysis and the H4 tail/acidic patch interaction is a well characterized contact that mediates inter-nucleosome interactions. Additionally, other groups have reported that the presence of DNA methylation causes condensation of both chromatin and bare DNA (cited within our manuscript), though the mechanics behind this phenomenon remain to be elucidated. We believed that our structure data may also align with those findings. However, the reviewer is fair in pointing out that we do not provide further experimental evidence in verifying the existence of these increased interactions. We can revise our writing to clarify that these points are currently hypotheses rather than validated results.

(2) It remains unclear whether DNA methylation alters global H2A.Z nucleosome stability or primarily affects local DNA end flexibility. Moreover, while the authors showed locus-specific accessibility by HinfI digestion, an unbiased assay such as MNase digestion would strengthen the conclusions.

We would like to thank the reviewer for bringing up these issues. Although our current data cannot explicitly clarify these possibilities, we favor an idea that DNA methylation specifically alters histone to DNA contacts and that this effect is felt globally across the entire nucleosome rather than only at specific locations. The intrinsic flexibility of linker DNA ends means that that region tends to exhibit the greatest differences under different physical influences, hence the focus on characterizing that area; flexibility of a thread on a spool is most pronounced at the ends. However, we also found that the DNA backbone of H2A.Z on methylated DNA had a lower local resolution compared to its unmethylated counterpart, despite that structure having a higher global resolution, which suggested to us that DNA positioning along the nucleosome is overall weaker under the presence of DNA methylation. This is corroborated by the increased population of open/shifted structures in our classification analysis. The reviewer raises a fair point about the use of a specific restriction enzyme versus MNase. We agree that our accessibility assay is highly influenced by the position of the restriction site and have previously seen that moving the cut site too close to the linker DNA end will abolish any DNA methylation-dependent differences. We did initially attempt an MNase digestion-based assay, but the data were not as reproducible as with the use of a specific restriction enzyme. We do not know the reason behind this irreproducibility though we believe that the processivity of MNase could make it difficult to capture subtle effects like those induced by DNA methylation on already highly accessible H2A.Z nucleosomes. Overall, while we believe that DNA methylation does exert a physical effect, its subtlety may explain the many contradictory studies present within the DNA methylation and nucleosome stability field.

References

Berta, D.G., H. Kuisma, N. Valimaki, M. Raisanen, M. Jantti, A. Pasanen, A. Karhu, J. Kaukomaa, A. Taira, T. Cajuso, S. Nieminen, R.M. Penttinen, S. Ahonen, R. Lehtonen, M. Mehine, P. Vahteristo, J. Jalkanen, B. Sahu, J. Ravantti, N. Makinen, K. Rajamaki, K. Palin, J. Taipale, O. Heikinheimo, R. Butzow, E. Kaasinen, and L.A. Aaltonen. 2021. Deficient H2A.Z deposition is associated with genesis of uterine leiomyoma. Nature. 596:398–403.

Capurso, D., H. Xiong, and M.R. Segal. 2012. A histone arginine methylation localizes to nucleosomes in satellite II and III DNA sequences in the human genome. BMC Genomics. 13:630.

Chodavarapu, R.K., S. Feng, Y.V. Bernatavichute, P.Y. Chen, H. Stroud, Y. Yu, J.A. Hetzel, F. Kuo, J. Kim, S.J. Cokus, D. Casero, M. Bernal, P. Huijser, A.T. Clark, U.

Kramer, S.S. Merchant, X. Zhang, S.E. Jacobsen, and M. Pellegrini. 2010. Relationship between nucleosome positioning and DNA methylation. Nature. 466:388–392.

Choy, J.S., S. Wei, J.Y. Lee, S. Tan, S. Chu, and T.H. Lee. 2010. DNA methylation increases nucleosome compaction and rigidity. J Am Chem Soc. 132:1782–1783.

Chua, E.Y., D. Vasudevan, G.E. Davey, B. Wu, and C.A. Davey. 2012. The mechanics behind DNA sequence-dependent properties of the nucleosome. Nucleic Acids Res. 40:6338–6352.

Collings, C.K., P.J. Waddell, and J.N. Anderson. 2013. Effects of DNA methylation on nucleosome stability. Nucleic Acids Res. 41:2918–2931.

Davey, C., S. Pennings, and J. Allan. 1997. CpG methylation remodels chromatin structure in vitro. J Mol Biol. 267:276–288.

Davey, C.S., S. Pennings, C. Reilly, R.R. Meehan, and J. Allan. 2004. A determining influence for CpG dinucleotides on nucleosome positioning in vitro. Nucleic Acids Res. 32:4322–4331.

Funabiki, H., I.E. Wassing, Q. Jia, J.D. Luo, and T. Carroll. 2023. Coevolution of the CDCA7-HELLS ICF-related nucleosome remodeling complex and DNA methyltransferases. Elife. 12.

Ghenoiu, C., M.S. Wheelock, and H. Funabiki. 2013. Autoinhibition and polo-dependent multisite phosphorylation restrict activity of the histone h3 kinase haspin to mitosis. Mol Cell. 52:734–745.

Jenness, C., S. Giunta, M.M. Muller, H. Kimura, T.W. Muir, and H. Funabiki. 2018. HELLS and CDCA7 comprise a bipartite nucleosome remodeling complex defective in ICF syndrome. Proc Natl Acad Sci U S A. 115:E876–E885.

Jimenez-Useche, I., J. Ke, Y. Tian, D. Shim, S.C. Howell, X. Qiu, and C. Yuan. 2013. DNA methylation regulated nucleosome dynamics. Sci Rep. 3:2121.

Jimenez-Useche, I., D. Shim, J. Yu, and C. Yuan. 2014. Unmethylated and methylated CpG dinucleotides distinctively regulate the physical properties of DNA. Biopolymers. 101:517–524.

Kelly, A.E., C. Ghenoiu, J.Z. Xue, C. Zierhut, H. Kimura, and H. Funabiki. 2010. Survivin reads phosphorylated histone H3 threonine 3 to activate the mitotic kinase Aurora B. Science. 330:235– 239.

Li, S., Y. Peng, D. Landsman, and A.R. Panchenko. 2022. DNA methylation cues in nucleosome geometry, stability and unwrapping. Nucleic Acids Res. 50:1864–1874.

Minary, P., and M. Levitt. 2014. Training-free atomistic prediction of nucleosome occupancy. Proc Natl Acad Sci U S A. 111:6293–6298.

Ngo, T.T., J. Yoo, Q. Dai, Q. Zhang, C. He, A. Aksimentiev, and T. Ha. 2016. Effects of cytosine modifications on DNA flexibility and nucleosome mechanical stability. Nat Commun. 7:10813.

Nishiyama, A., L. Yamaguchi, J. Sharif, Y. Johmura, T. Kawamura, K. Nakanishi, S. Shimamura, K. Arita, T. Kodama, F. Ishikawa, H. Koseki, and M. Nakanishi. 2013. Uhrf1-dependent H3K23 ubiquitylation couples maintenance DNA methylation and replication. Nature. 502:249–253.

Osakabe, A., F. Adachi, Y. Arimura, K. Maehara, Y. Ohkawa, and H. Kurumizaka. 2015. Influence of DNA methylation on positioning and DNA flexibility of nucleosomes with pericentric satellite DNA. Open Biol. 5.

Perez, A., C.L. Castellazzi, F. Battistini, K. Collinet, O. Flores, O. Deniz, M.L. Ruiz, D. Torrents, R. Eritja, M. Soler-Lopez, and M. Orozco. 2012. Impact of methylation on the physical properties of DNA. Biophys J. 102:2140–2148.

Segal, E., Y. Fondufe-Mittendorf, L. Chen, A. Thastrom, Y. Field, I.K. Moore, J.P. Wang, and J. Widom. 2006. A genomic code for nucleosome positioning. Nature. 442:772–778.

Wassing, I.E., A. Nishiyama, R. Shikimachi, Q. Jia, A. Kikuchi, M. Hiruta, K. Sugimura, X. Hong, Y. Chiba, J. Peng, C. Jenness, M. Nakanishi, L. Zhao, K. Arita, and H. Funabiki. 2024. CDCA7 is an evolutionarily conserved hemimethylated DNA sensor in eukaryotes. Sci Adv. 10:eadp5753.

Zilberman, D., D. Coleman-Derr, T. Ballinger, and S. Henikoff. 2008. Histone H2A.Z and DNA methylation are mutually antagonistic chromatin marks. Nature. 456:125–129.

**Recommendations for the authors:**

**Reviewer #1 (Recommendations for the authors):**

The authors designed two sets of experiments to explore the molecular mechanisms underlying the mutually exclusive distribution of H2A.Z and DNA methylation previously reported by several groups.

First, they examined how DNA methylation affects the physical stability of H2A.Z-containing nucleosomes. Although their results point to subtle differences between nucleosomes assembled on methylated versus unmethylated DNA, the authors did not extend their analyses to directly test the stability of these H2A.Z-containing nucleosomes under more challenging conditions. Prior studies have demonstrated that certain nucleosomes, such as those containing H3.3-H2A.Z or H2A.Z-H3K56Q, exhibit specific instability, but such instability is only revealed under challenging conditions, for example, altered salt concentrations or the presence of additional factors like FACT (PMID: 17575053; PMID: 19633671; PMID: 19639024; PMID: 41303375). In light of this literature, the observable structural features noted here for nucleosomes containing H2A.Z and methylated DNA are suggestive of increased instability, yet the authors did not employ comparable approaches to rigorously test whether such instability might explain the absence of H2A.Z from methylated genomic regions.

As a result, at this stage of analysis, the idea that nucleosomes containing both H2A.Z and methylated DNA are intrinsically unstable, and that this instability accounts for the depletion of H2A.Z from methylated regions, remains unsubstantiated.

We thank the reviewer's constructive criticisms. Through our response to these points, we were able to significantly improve our manuscript, including major rewriting of the Abstract and Discussion as well as incorporation of new data.

We agree that combinations with other histone variants, modifications, and mutations could further affect our observed impact of DNA methylation on H2A.Z-nucleosome stability. What we observed based on satellite II-derived DNA was that DNA methylation made H2A.Znucleosomes (with H3.2) more open, although the effect of DNA methylation is relatively small (as compared to the general impact of H2A.Z incorporation). We readily admit that such a subtle physical effect is unlikely to be the main driver of the antagonistic distribution of H2A.Z and DNA methylation, though small physical changes have been known to influence larger biological functions, and sought to describe additional regulatory factors that could play major roles.

We also agree that H3.3 is of major interest when discussing H2A.Z. In our Xenopus egg extract experiments using DNA beads, the primary H3 variant deposited is H3.3 as no DNA replication occurs on the beads to allow for H3.1/.2 replication-coupled deposition. From those experiments, we demonstrated that preferential loading of H2A.Z can be primarily explained by SRCAP. In other words, in the absence of SRCAP, loading/retention of H2A.Z on H3.3nucleosomes was not noticeably affected by DNA methylation, indicating that DNA methylation’s physical effects on H2A.Z nucleosomes plays little, if any, role in the preferential accumulation of H2A.Z on unmethylated DNA at least in the context of synthetic DNA beads incubated in

Xenopus egg extract lacking active transcription. Our sequencing data hints at the interesting possibility that transcription, along with other factors missing in egg extract, may be involved in further pruning H2A.Z from methylated DNA which conceivably could take advantage of subtle physical alterations. However, we agree we lack firm supporting evidence for such a mechanism which led us to forgo including that in our final model figure and we instead only report on our observations with discussions on potential biological implications and limitations. Of note, it has been reported that the H2A.Z nucleosome is more accessible than the H2A nucleosome, while inclusion of H3.3 does not further enhance accessibility of the H2A.Z nucleosome (PMID 38920622). We have now noted these points in the Discussion of our revised manuscript.

We appreciate and agree with this reviewer’s point that nucleosome instability sometimes requires challenging conditions to be fully revealed. However, in our system, use of H2A.Z was the challenge provided as we find in our hands that H2A.Z by itself substantially destabilizes histone-DNA contacts compared to canonical H2A. And it is only with this already destabilized nucleosome that we see further enhancement of accessibility/openness in the presence of DNA methylation. This is similar to findings by [PMID: 23260052] that reported that only an intrinsically destabilized sub-population of canonical H2A nucleosomes on 601 DNA experienced detectable physical changes in the presence of DNA methylation.

In response to this reviewer's comment, we edited the Abstract and Discussion to clearly note the subtly of the impact of DNA methylation on H2A.Z nucleosome structure, and that the potential functional significance remains an open question.

Second, the authors investigated whether SRCAP-C contributes to preferential H2A.Z incorporation into unmethylated DNA. The absence of H2A.Z from methylated regions does not necessarily imply that it cannot be incorporated there; it may instead reflect the chromatin environment associated with DNA methylation, which could disfavor SRCAP-C activity, whereas open chromatin environments strongly promote SRCAP-dependent H2A.Z deposition.

This reviewer suggested an alternative model where SRCAP prefers to act on open chromatin and that the apparent preferential H2A.Z deposition to unmethylated DNA is due solely to the increased accessibility associated with unmethylated DNA. Following such a model, one would predict that SRCAP-C's preference to unmethylated DNA would be eliminated on nucleosome-free DNA in Xenopus egg extracts. To test this alternative model, we repeated the SRCAP-C binding experiment in egg extracts depleted of the HIRA complex, the H3.3-H4 chaperone responsible for de novo nucleosome assembly on exogenously added DNA in egg extracts. Contrary to this prediction, both SRCAP and ZNHIT1 still display preferential binding to unmethylated DNA substrates in HIRA-depleted extracts in which nucleosome assembly is suppressed (newly added Suppl Fig 16). The results argue that discrimination of SRCAP-C from methylated DNA is not due to a potential effect of chromatin compaction by DNA methylation. Furthermore, our new result is in line with an idea that SRCAP employs 1D diffusion on the linker DNA before engaging the H2A nucleosome (PMID 39131301), implying that discrimination of SRCAP-C from methylated linker DNA contributes to this process. This is now illustrated in the new model Figure 6.

Please note we also indicate in both our model and in text that there exists an additional methylation-insensitive mechanism that drives H2A.Z deposition on methylated DNA, leading to a substantial amount of colocalized H2A.Z and DNA methylation. Why two different deposition pathways for H2A.Z differing in their methylation sensitivities must exist is an interesting topic for future work and has not been described prior to our report.

This interpretation is consistent with the authors' own comparative mapping of H2A.Z and DNA methylation in sperm pronuclei incubated in egg extract versus a transcriptionally active Xenopus fibroblast line. They observed that about 40% of H2A.Z-associated genomic DNA is methylated in sperm pronuclei, but only 3% in fibroblasts. As they note, the major difference between these systems is the presence of transcription in fibroblasts, a process known to drive H2A.Z eviction/recycling, and which is absent in the egg-extract system. Thus, no specific inhibition of SRCAP-C by methylated DNA needs to be invoked: H2A.Z deposition on both methylated and unmethylated accessible regions, followed by preferential eviction from methylated sites in active nuclei, could fully account for the observed patterns.

As the reviewer correctly notes here, we proposed that transcription is likely to play an important role in pruning H2A.Z from methylated DNA. Our observations and proposed mechanism do not argue against the possible existence of a DNA methylation-insensitive, transcription-dependent mechanism that promotes dissociation of H2A.Z from methylated DNA, which we believe likely would be correlated to gene body methylation. In fact, we did propose in our Discussion that such a transcription-mediated mechanism may conceivably take advantage of the subtly destabilized DNA wrapping of H2A.Z nucleosomes on methylated DNA to further selectively prune H2A.Z at colocalized regions. However, such a mechanism would be an additional component to what we have already described and does not explain the observed preferential recruitment of SRCAP-C to unmethylated DNA in Xenopus egg extracts in the absence of active transcription.

In this respect, studies from the Felsenfeld laboratory showing that double-variant nucleosomes are highly unstable under physiological ionic conditions are particularly relevant (PMID: 19633671; PMID: 19639024). They demonstrated that such unstable nucleosomes are only evident under low ionic strength extraction conditions, emphasizing that the apparent absence of H2A.Z may reflect facilitated removal rather than failure of assembly.

The authors may also have been influenced by the study of Berta et al. (cited in the manuscript), which examined uterine leiomyomas harboring somatic or germline mutations in SRCAP-C subunits. In those tumors, the normal association of H2A.Z with accessible, active chromatin, and its exclusion from methylated regions, was lost. However, this observation does not demonstrate that SRCAP-C actively prevents H2A.Z incorporation into methylated DNA. Instead, it may simply reflect that in the absence of SRCAP-C, a default, less efficient deposition pathway operates regardless of whether the chromatin environment is normally permissive or restrictive for SRCAP-dependent activity.

Even if one accepts the more straightforward interpretation proposed by the present authors, that SRCAP-C is actively inhibited by methylated DNA, as suggested by their pull-down experiments from Xenopus egg extracts using unmethylated and methylated DNA, the hypothesis lacks mechanistic support.

Considering this reviewers' criticism, we have expanded our discussion to indicate a possibility that SRCAP-C may have an alternative mechanism to find open chromatin independent of DNA methylation status. However, our data show that SRCAP-C preferentially binds to unmethylated DNA in a manner independent of transcription or other epigenetic status in Xenopus egg extracts, and that SRCAP-C carries the major mechanism that explains preferential deposition of H2A.Z to unmethylated DNA. Therefore, we believe that our study for the first time offers a mechanistic explanation of how H2A.Z discrimination from methylated DNA is accomplished through SRCAP-dependent H2A.Z deposition.

The following points summarize the issues discussed above:

(1) The authors did not sufficiently test the hypothesis that H2A.Z-methylated DNA nucleosomes are inherently unstable and could explain the exclusion of H2A.Z from methylated genomic regions.

We stand by our conclusion that DNA methylation has an intrinsic capacity to make the H2A.Z nucleosome more open and accessible, even though the effect is subtle. We did not argue that this subtle effect can fully explain the exclusion of H2A.Z from methylated genomic regions. Rather, our Xenopus egg extract experiment suggested that in the transcriptionally inactive egg extract setting, such a mechanism plays little or no role and it is SRCAP-C instead that is the major driver. Whether this physical mechanism also contributes to their exclusion in cells with active transcription remains a future subject of study.

(2) The proposed active role of SRCAP-C in preventing H2A.Z assembly on methylated DNA is supported only by limited experimental data and lacks a mechanistic explanation. In particular, this hypothesis does not account for the significant H2A.Z assembly observed on methylated DNA regions in sperm nuclei after incubation in egg extract.

We respectfully disagree with this summary assessment. Our conclusions are well aligned with the substantial H2A.Z association with methylated DNA in sperm pronuclei assembled in Xenopus egg extracts seen. We demonstrated that:

(1) In transcriptionally-silent Xenopus egg extracts using synthetic DNA beads, DNAbinding of SRCAP-C is inhibited by DNA methylation.

(2) In this set up, H2A.Z is preferentially, if not exclusively, loaded to unmethylated DNA over methylated DNA.

(3) Depletion of SRCAP-C almost completely eliminated preferential association of H2A.Z to unmethylated DNA, while leaving some DNA methylation-insensitive H2A.Z loading.

(4) These data indicate the presence of a SRCAP-C-dependent, DNA methylationsensitive mechanism as well as a SRCAP-C-independent, DNA-methylation-insensitive mechanism to load H2A.Z to chromatin. This conclusion matches well with our genomic analysis showing that H2A.Z is preferentially but not exclusively loaded to hypomethylated genomic segments to sperm pronuclei in Xenopus egg extracts.

(5) As we clearly discussed, this SRCAP-C-dependent mechanism by itself is insufficient to explain the much clearer exclusion of H2A.Z in somatic cells. We discussed the possibility that transcription contributes to further pruning of H2A.Z from methylated DNA.

To deliver this overall message with nuances that we noted above, we have heavily revised the Abstract, the model Figure 6, and Discussion. Thanks to the criticisms raised by this reviewer, we believe that our revised manuscript has been significantly improved.

**Reviewer #2 (Recommendations for the authors):**

(1) A major omission is the absence of a cryo-EM structure of a canonical nucleosome assembled on the same DNA template - this is essential to assess whether the observed effects are H2A.Z-specific.

We had considered solving the H2A structures, however, ultimately decided against it for a few reasons. First, there already exists crystal structures of canonical H2A nucleosomes using a DNA sequence highly similar to our Sat2R-P with and without the presence of DNA methylation (PDB: 5CPI and 5CPJ). The authors of this study did not see any physical differences present in their structures (Osakabe et al., 2015). Additionally, we had included canonical H2A conditions within our restriction enzyme accessibility assay and did not see a significant impact of DNA methylation on those samples (Fig 3). Because of the previous report and our own negative data, we expected that only limited additional insights would be obtained from the canonical H2A structures and decided not to pursue that analysis, considering the cost and effort for this additional cryo-EM analysis.

(2) The reported increase in accessibility of the methylated H2A.Z nucleosome is negligible compared with the much larger intrinsic DNA accessibility of the unmethylated H2A.Z nucleosome. Claims that methylated H2A.Z nucleosomes are "more open and accessible" must therefore be removed, and the title is misleading, given that no meaningful impact of DNA methylation on H2A.Z nucleosome stability is demonstrated.

We respectfully disagree with this reviewer's criticism. We investigated the potential impact of DNA methylation on nucleosome stability to the best of our abilities through complementary assays and reported our observations. The effect of DNA methylation is smaller than the difference between H2A.Z and H2A, but we were able to see an effect. It is also not uncommon for small differences to have functional impacts in biological systems. We agree that further testing is required to determine whether this subtle effect is functionally important, and it remains the subject of future research due to the many technical challenges associated with addressing said question. We would like to note that 18 years have passed since Daniel Zilberman first reported the antagonistic relationship between H2AZ and DNA methylation (Zilberman et al., 2008) but very few studies have since directly tested specific mechanistic hypotheses. We believe that our study lays the groundwork for exciting future investigation that better elucidates the pathways that contribute to this antagonism and will have meaningful impacts on the field in general. However, thanks to the reviewer's criticism, we realized that we did not clearly state in the Abstract that the effect of DNA methylation on intrinsic H2A.Z nucleosome stability is relatively subtle. We will accordingly revise the Abstract, the model Figure 6, and Discussion to make this point clearer.

(3) The cryo-EM structures of methylated and unmethylated 601L H2A.Z nucleosomes show no detectable differences. As presented, this negative result adds little value and should be removed.

We believe the inclusion and factual reporting of negative data is important for the scientific community as one of the major issues currently in biology research is biased omission of negative data. We considered eLife as a venue to publish this work for this reason. We understand that the reviewer believes our 601L structures may detract from the overall message of our manuscript, however, we believe that this data rather emphasizes the importance of DNA sequence context, something that the reviewer also rightfully notes. It is standard practice in the nucleosome field to use the Widom 601 sequence, along with its variants. Our experience has shown that use of an artificially strong positioning sequence may mask weaker physical effects that could play a physiological role. Thus, we were careful to validate all further assays with multiple DNA sequences and believed it important to report these sequence-dependent effects on nucleosome structure.

(4) Very little H3 signal coincides with H2A.Z at TSSs in sperm pronuclei, yet this is neither explained nor discussed (Supplementary Figure 10D). The authors need to clarify this.

Our H3 signal, which represents the global nucleosome population, is more broadly distributed across the genome than H2A.Z, which is known to localize at specific genomic sites. Since both histone types were sequenced to similar read depths, H3 peaks are generally shallower than H2A.Z and peak heights cannot be directly compared (i.e. they should be represented in separate appropriate data ranges).

(5) In my view, the most conceptually important finding is that H2A.Z-associated reads in sperm pronuclei show ~43% CpG methylation. This directly contradicts the model of strict mutual exclusivity and suggests that the antagonism is context-dependent. Similarly, the finding that the depletion of SRCAP reduces H2A.Z deposition only on unmethylated templates is also very intriguing. Collectively, these result warrants further investigation (see below).

(6) Given that H2A.Z is located at diverse genomic elements (e.g., enhancers, repressed gene bodies, promoters), the manuscript requires a more rigorous genomic annotation comparing H2A.Z occupancy in sperm pronuclei versus XTC-2 cells. The authors should stratify H2A.ZDNA methylation relationships across promoters, 5′UTRs, exons, gene bodies, enhancers, etc., as described in Supplementary Figure 10A.

We appreciate recognition of the importance of our finding by this reviewer. We agree that the substantial presence of co-localized H2A.Z and DNA methylation specifically in the sperm pronuclei samples and the changes in pattern between nuclear types are highly interesting and require further investigation. However, we faced technical challenges in our sequencing experiments that made us refrain from conducting a more detailed analysis for fear of over-interpreting potential artifacts. These challenges mainly stemmed from the difficulties in collecting enough material from Xenopus egg extracts and Tn5’s innate bias towards accessible regions of the genome. Because of this, open regions of the genome tend to be overrepresented in our data (as noted in our Discussion), making it challenging to rigorously compare methylation profiles and H2A.Z/H3 associated genomic elements.

While the degree of separation seems to be dependent on nuclei type, we still believe the antagonism exists in both the sperm pronuclei and XTC-2 samples when comparing H2A.Z methylation profiles to the corresponding H3 condition. Our study also demonstrates that H2A.Z is preferentially deposited to hypomethylated DNA in a manner dependent of SRCAP-C (the loss of SRCAP only reduces H2A.Z on unmethylated substrates) but an additional methylationinsensitive H2A.Z deposition mechanism also exists. We realized that this interesting point was not clearly highlighted in Abstract, so we will revise it accordingly.

(7) Although H2A.Z accumulates less efficiently on exogenous methylated substrates in egg extract, substantial deposition still occurs (~50%). This observation directly challenges the strong antagonistic model described in the manuscript. The authors need to discuss this in more detail.

As depicted in Figure 6 and described in the Discussion, we indicated that both methylation-sensitive and methylation-insensitive pathways exist to deposit H2A.Z within the genome. We also directly stated in our Discussion that a substantial proportion of H2A.Z colocalizes with DNA methylation both in our study as well as in previous reports, which is of major interest for future study. Additionally, we further discussed how the absence of transcription in Xenopus eggs is a likely reason for the more limited effect of DNA methylation restricting H2A.Z deposition in our egg extract system. In the revised manuscript, we heavily edited the Discussion to better clarify these points.

(8) The SRCAP depletion is insufficiently validated, i.e., the antibody-mediated depletion of SRCAP lacks quantitative verification. A minimum of three biological replicates with quantification is required to substantiate the claims.

In response to this, quantification of the SRCAP depletion is now included as Supplementary Figure 13A and B. Since our anti-ZNHIT1 antibodies reproducibly detected ZNHIT1 on DNA beads isolated from egg extracts, we have conducted additional verification of the SRCAP depletion by probing for SRCAP and ZNHIT1 on DNA beads, confirming that these proteins were depleted on DNA beads upon immunodepletion with anti-SRCAP antibodies (Author response image 1). To further validate this conclusion, we added data showing that the effect of SRCAP depletion on methylation-sensitive H2A.Z deposition was reproduced through use of a different commercially available antibody raised against human SRCAP (newly added Suppl Fig 14).

**Author response image 1.** Verification of SRCAP depletion using DNA beads. DNA beads were incubated in interphase-cycled *Xenopus* egg extract that had been depleted with either our custom SRCAP antibody or an IgG negative control. SRCAP and ZNHIT1 association was then assessed via Western Blot.

(9) It appears that the role of p400-Tip60 has been completely overlooked. This complex is the second major H2A.Z deposition complex. Because p400 exhibits DNA methylation-insensitive binding (Supplementary Figure 14), it may account for the deposition of H2A.Z onto methylated DNA. This possibility is highly significant and must be addressed by repeating the key experiments in Figure 5 following p400-Tip60 depletion.

Thank you very much for raising this interesting point. We were aware that the TIP60 complex is a very likely candidate for mediating DNA methylation-insensitive H2A.Z deposition, which is why we tested whether DNA binding of p400 is methylation sensitive (shown in the revised Supplementary Figure 15). We wished to test the potential contribution of TIP60-C, but, unfortunately, the antibodies we currently have available to us were not successful in depleting the complex from egg extract. Since we had no direct experimental evidence indicating the role TIP60-C plays, we decided to take a conservative approach to our model and leave the methylation-insensitive pathway as mediated by something still unidentified. While further investigating TIP60-C’s contribution to this pathway is of definite value, we do not believe that it impacts our major conclusion that SRCAP-C is the main mediator responsible for H2A.Z deposition on unmethylated DNA and thus remains a subject for future study. However, we have now added descriptions to note that TIP60-C is a likely candidate to execute the SRCAPindependent and methylation-insensitive mechanism of H2A.Z loading in Xenopus egg extracts. In the model figure, we initially did not include Tip60-C, but we now infer TIP60-C is a likely candidate in the revised model (Figure 6) to facilitate the future research in the field.

(10) The manuscript repeatedly states that H2A.Z nucleosomes are intrinsically unstable; however, this is an oversimplification. Although some DNA unwrapping is observed, multiple studies show that H3/H4 tetramer-H2A.Z/H2B interactions are more stable (important recent studies include the following: DOI: 10.1038/s41594-021-00589-3; 10.1038/s41467-021-22688-x; and reviewed in 10.1038/s41576-024-00759-1). These references should be considered.

We appreciate that the reviewer points out this important issue. Although we had described that controversy exists regarding how H2A.Z and DNA methylation contributes to nucleosome stability, it was not clearly explained. We understand that this confusion was in part due to the term “nucleosome stability”, which is broad and encompasses many physical aspects. As noted in a prior response, we now better specify our use of the term within the manuscript, emphasizing the nucleosome openness and accessibility, particularly at the nucleosome core particle entry/exit sites. As noted by published studies (PMID 38920622), the impact on nucleosome stability may differ between the internal and external segments of nucleosomal DNA. In our assays, we are most focused on the DNA wrapping stability of the nucleosome and have consistently seen in our hands that H2A.Z nucleosomes are much more open and accessible at DNA ends compared to canonical H2A on satellite II-derived sequences, regardless of methylation status. However, we do understand that many groups have observed the opposite findings while others have obtained results similar to us. This may be caused by usage of different assays (for example, nucleosome assembly during salt dialysis or salt sensitivity vs openness/accessibility of preassembled nucleosome). In the Discussion of the revised manuscript, we now explain these factors, with the hope that our study will help clarify some of the field’s controversies.

**Reviewer #3 (Recommendations for the authors):**

(1) Since the cryo-EM structure determined by single-particle analysis represents only one major population, it would be important to determine the dyad axis position by complementary biochemical assays, such as MNase-seq or chemical digestion by the Fenton reaction (PMID: 22929776).

We would like to thank the reviewer for bringing up this important issue. We agree that the high-resolution structure represents only a subpopulation in which we specifically selected for the most stably wrapped nucleosomes in each sample. This issue is why we then supplemented our high-resolution structure with our *in-silico* classification analysis to survey the overall structure distribution of the full nucleosome particle population. The classification input contains all nucleosome-like particles picked from both unmethylated and methylated sample micrographs mixed together, ensuring that all particles are taken into consideration and that both samples have been analyzed in an identical manner. From our sorting analysis, we find an increased population of open and shifted nucleosome structures present in our methylated DNA sample, indicating destabilization of DNA-histone wrapping with DNA methylation. This is corroborated by the lower local resolution seen on the DNA backbone of our high-resolution H2A.Z on methylated DNA structure, despite it having a higher global resolution compared to its unmethylated counterpart. This suggested to us that DNA positioning along the nucleosome is overall weaker under the presence of DNA methylation.

The reviewer raises a fair point about the use of a specific restriction enzyme versus MNase. We agree that our accessibility assay is highly influenced by the position of the restriction site and have previously seen that moving the cut site too close to the linker DNA end will abolish any DNA methylation-dependent differences. We realized that we did not explain how we decided to place the HinfI site in the context of our solved cryo-EM structure. In the revised Figure 3B, we now illustrate that the HinfI site is located at a segment where H2A/H2A.Z directly contacts the DNA and explained that this segment belongs to the region that exhibited clear methylation-induced flexibility in our cryo-EM structures. Thus, our structure helped us design this experiment.

We did initially attempt an MNase digestion-based assay, but the data were not as reproducible as with the use of a specific restriction enzyme. We do not know the reason behind this irreproducibility though we believe that the processivity of MNase could make it difficult to capture subtle effects like those induced by DNA methylation on already highly accessible H2A.Z nucleosomes, as subtle technical errors in the MNase concentration can have significant effects. Overall, while we believe that DNA methylation does exert a physical effect, its subtlety may explain the many contradictory studies present within the DNA methylation and nucleosome stability field.

(2) I assume that the authors confirmed complete DNA methylation by restricted enzyme digestion. It would be helpful to include this validation in supplementary figures.

We would like to thank the reviewer for pointing out that this critical verification was missing from our initial manuscript. DNA methylation of Sat2R-P and Sat2R was verified via BstBI digestion (Suppl Fig 1B and 7D, respectively); 601L verified with HpaII digestion (Suppl Fig 6B); and 19x601 DNA verified via BstUI digestion (Suppl Fig 11A). All data has been added to the specified figures. Unfortunately, the 16xHSat2 DNA substrate we used in our assays does not contain appropriate cut-sites for methylation-sensitive restriction enzymes. Due to that, we always prepared the 16xHSat2 DNA in parallel with the 19x601 substrate under identical conditions then use digestion of the 19x601 substrate to verify quality of methylation for each batch. To more directly verify methylation of 16xHSat2 DNA, we used *Xenopus laevis* ZHX2 and ZHX3, which we recently identified as proteins that selectively associate with methylated DNA in Xenopus egg extracts. Although identification and characterization of Xenopus ZHX2/3 will be described elsewhere, previous published proteomic studies have also identified mammalian ZHXs as proteins that enrich on methylated DNA (PMID 21029866, 23434322). By incubating DNA beads in Xenopus egg extract and probing for endogenous ZHX2/3 (our antibody recognizes both ZHX2 and ZHX3), we verified that ZHXs selectively binds to methylated 16xHSat2 but not unmethylated DNA (Author response image 2). Although this does not necessarily verify that all CpGs in 16xHSat2 were methylated, we observed comparable methylation-induced inhibition of SRCAP binding between 16x601 and 16HSat2, supporting our conclusion.

**Author response image 2.** Verification of 16xHSat2 methylation status via ZHX2/3 protein binding. 16xHSat2 DNA beads were incubated in *Xenopus* egg extract and endogenous ZHX2/3 protein binding assessed via Western Blot with a custom generated antibody that recognizes both ZHX2 and ZHX3.

(3) Figure 1A: The dyad position is difficult to identify. Please indicate it clearly using a distinct color (not green).

We now directly indicate each sequence midpoint with a black triangle and also changed the font of DNA sequences to further clarify that the dyad resides at the palindromic center.
